# Genetic analysis of Upland cotton dynamic heterosis for boll number per plant at multiple developmental stages

**DOI:** 10.1038/srep35515

**Published:** 2016-10-17

**Authors:** Lianguang Shang, Yumei Wang, Shihu Cai, Lingling Ma, Fang Liu, Zhiwen Chen, Ying Su, Kunbo Wang, Jinping Hua

**Affiliations:** 1 Department of Plant Genetics and Breeding/Key Laboratory of Crop Heterosis and Utilization of Ministry of Education/Beijing Key Laboratory of Crop Genetic Improvement, China Agricultural University, Beijing 100193, China; 2 Research Institute of Cash Crops, Hubei Academy of Agricultural Sciences, Wuhan 430064, Hubei, China; 3 Institute of Cotton Research, Chinese Academy of Agricultural Sciences/State Key Laboratory of Cotton Biology, Anyang 455000, Henan, China

## Abstract

Yield is an important breeding target. As important yield components, boll number per plant (BNP) shows dynamic character and strong heterosis in Upland cotton. However, the genetic basis underlying the dynamic heterosis is poorly understood. In this study, we conducted dynamic quantitative trait loci (QTL) analysis for BNP and heterosis at multiple developmental stages and environments using two recombinant inbred lines (RILs) and two corresponding backcross populations. By the single-locus analysis, 23 QTLs were identified at final maturity, while 99 QTLs were identified across other three developmental stages. A total of 48 conditional QTLs for BNP were identified for the adjacent stages. QTLs detected at later stage mainly existed in the partial dominance to dominance range and QTLs identified at early stage mostly showed effects with the dominance to overdominance range during plant development. By two-locus analysis, we observe that epistasis played an important role not only in the variation of the performance of the RIL population but also in the expression of heterosis in backcross population. Taken together, the present study reveals that the genetic basis of heterosis is dynamic and complicated, and it is involved in dynamic dominance effect, epistasis and QTL by environmental interactions.

Heterosis is the phenomenon that a hybrid has the better performance than either of its parents. Heterosis is extensively exploited by plant breeders to the benefit of agriculture, however, its molecular genetic mechanism are not well understood. The development of molecular markers for genetic analyses promoted understanding the genetic basis underlying heterosis^1^,^2^. So far, three major models have been proposed to explain heterosis: the dominance, over-dominance and epistasis^3^. Creating heterozygotes by testcross or backcross populations from permanent populations (doubled haploid or recombinant inbred line population) could allow the repeated analysis of heterosis^4^. Studies of quantitative trait loci (QTL) mapping using testcross or backcross populations in crops, such as rice^5^,^6^,^7^,^8^, maize^9^, rape^10^,^11^ show that there is not a single explanation for hybrid vigor in various crops^3^. In addition, the genetic basis for heterosis in yield is quite different for maize and for rice. This distinction seems to be related to open or self-pollination of the respective species^12^. It is necessary to systemically study the genetic mechanism of heterosis in Upland cotton (cross pollination).

Upland cotton is widely cultivated and contributed most of the natural commercial textile fiber in the world. Mapping QTL for yield and yield components will facilitate the application of marker-assisted selection (MAS) to improve yield in Upland cotton. Seed cotton yield per plant was the product of its two direct components, boll number per plant (BNP) and boll weight. As yield components, BNP showed strong heterosis in previous reports^13^,^14^,^15^,^16^,^17^. The number of bolls per plant is the trait with the highest heterosis in traits of yield components^16^. In addition, Shang *et al.*^17^ studied heterosis of two Upland cotton hybrids and the result also suggested that the BNP trait was the biggest contributor to yield and yield heterosis, compared with other yield components. Some studies on QTLs analysis for fiber trait were conducted in cotton^18^,^19^,^20^. For yield and yield heterosis, Shen *et al.*^21^ detected five QTLs for bolls per plant using RILs population. Liu *et al.*^15^ developed the immortalized F_2_ (IF_2_) population based on RIL population and found that both dominance and over-dominance contributed to heterosis of ‘XZM 2’ hybrid, and dominance played more important role in cotton yield performance. Guo *et al.*^22^ conducted heterotic QTL (hQTL) mapping for yield traits using chromosome segment introgression lines and the results suggested that the over-dominant effect mainly contributed to yield heterosis. Ning *et al.*^23^ developed 180 RILs from a cross between Prema and Chinese cultivar ‘86–1’ and identified thirteen QTLs for seed-cotton yield. Wang *et al.*^24^ identified 70 QTLs for yield components using 178 RILs derived from a cross between DH962 and Jimian5. Liang *et al.*^16^ conducted study of yield heterosis in F_2:3_ and F_2:4_ populations derived from a hybrid ‘Xinza 1’ and indicated that partial dominance and overdominance effects at the single locus level and epistasis at two-locus level elucidated the genetic basis of heterosis in Upland cotton. Recently, Shang *et al.*^17^ constructed two backcross (BC) populations based on two corresponding recombinant inbred line (RIL) populations and suggested that partial dominance, overdominance, epistasis, and QTL by environment interaction all contribute to heterosis in Upland cotton. Sequences of the D_5_-genome (*Gossypium raimondii*), A_2_-genome (*G. arboreum*) and genome sequence of cultivated Upland cotton (*G. hirsutum* TM-1) available^25^,^26^,^27^,^28^,^29^ will facilitate development of new markers for high-resolution genetic map construction, and further dissect the genetic basis of complex quantitative traits as well as heterosis in Upland cotton.

The QTL analysis for yield and yield heterosis is usually studied using the final trait at maturation stage in cotton or other crops. These reports overlooked the distinct hQTL actions at different developmental stages. The phenotypic value of quantitative trait at maturity which was used for QTL mapping mainly reflected the cumulative effects of QTL. Recent studies confirmed that QTLs expressed selectively at different developmental stages and it was not fully representative to better explain the genetic basis of quantitative character only by QTL mapping at maturity in Upland cotton^30^,^31^,^32^. In addition, boll number per plant is a dynamic trait which shows strong phenotypic change during plant development in Upland cotton. Therefore, it is necessary to study dynamics of QTL and hQTL expression for boll number per plant at different developmental stages.

In present research, we used previously constructed two Upland cotton BC populations and two RIL populations, and conducted QTL and conditional QTL analysis for boll number per plant as well as heterosis performance under multiple developmental stages and environments. The QTL and hQTL were analyzed at single-locus and two-locus levels at multiple developmental stages. This research will provide new insights into understanding of the genetic basis of dynamic heterosis in Upland cotton.

## Results

### Performance of boll number per plant

The performance of boll number per plant for RIL(V), BC(V) and MPH data in three environments is shown in Table 1. Heterosis of the XZV hybrid is generally larger than that of the XZ hybrid. In XZ and XZV hybrids, the parent ‘GX1135’ possessed significant higher phenotypic value than ‘GX100-2’ and ‘VGX100-2’ for BNP at four developmental stages respectively. Significant heterosis for BNP was shown in two hybrids populations. Wide ranges of variation are observed in the RIL(V), BC(V) and MPH data. Moreover, the BNP showed different levels of heterosis at different developmental stages. The means of the BC(V) population were higher than the RIL(V)s for most of traits at three environments. The order of means of the MPH (%) for BNP is *t*1 > *t*2 > *t*3 > *t*4. All of extreme lines in the RIL(V) and BC(V) populations exceeded those of hybrids and CK at four stages at three locations, Meanwhile, many individuals showed higher MPH in the BC population than MPH of the two hybrids (Table 1). The means of populations expressed transgressive segregation at four stages. The analysis of variance was conducted and significant genotypic variances, environmental variances and genotype × environmental interaction variances for BNP were observed at four stages (Table S1). These results indicated that BNP of RIL(V) and BC(V) populations showed a big variation and it was conducive for QTL analysis.

### Relationships between the means of RILs, MPH, and BC performance

For XZ and XZV hybrids, the correlation coefficients among the mean values of BC(V)s, their mid-parent heterosis (MPH) and the means of RILs for the BNP trait at four developmental stages are shown in Table 2. Significant high positive correlations between MPH and BC(V)s performance were observed for all the developmental stages in three environments. Most of the trait values of the RIL(V)s and that of their BC(V)s showed significant positive correlation in two hybrids. Population performance of the BC(V) for most of traits was largely determined by performance of the parental RIL(V)s at four developmental stages. There were negative correlations between most of trait values of RIL(V)s and MPH in three environments.

### Relationship between heterozygosity and dynamic performance

Most of correlation coefficients are not significant between heterozygosity of whole-genome and the dynamic performance of BC(V) and MPH data in terms of BNP trait at four developmental stages in two hybrids (Table 3). The result suggested that overall genome heterozygosity alone possessed little effect on dynamic trait performance. Heterosis might derived from just a small of genome heterozygosity in the BC(V) data at not only early stage but also maturity of cotton plant. The low correlation coefficients may be the result of low density loci for the whole genome and only half of heterozygosity of whole-genome existed in backcross population. These results support the previous studies^4^,^33^,^34^.

### Single-locus QTL

Genetic maps for the two hybrid populations were previously constructed by the polymorphic loci (Figure S1). For the XZ hybrid, 623 loci were mapped to 32 linkage groups and the genetic map spanned 3,889.9 cM. For the XZV hybrid, 308 loci were mapped to 39 linkage groups and the genetic map spanned 3,048.4 cM^17^.

QTLs detected using composite interval mapping in XZ and XZV hybrids are shown in Table S2 at the single-locus level. Table 4 listed numbers for different effect of QTLs identified by composite interval mapping at four developmental stages at three environments in backcross population. A total of 38 and 22 QTLs were respectively detected for BNP at four developmental stages of XZ and XZV hybrids.

In the XZ hybrid, a total of 38 QTLs were detected in RIL, BC, and MPH data sets (Table S2). Twenty-one QTLs were identified in more than two developmental stages or environments or populations. Totally, 22, 22, 16, and 14 QTLs were respectively detected at stage *t*1, *t*2, *t*3, and *t*4 in three data sets. In backcross population, 19 QTLs with an additive effect, nine with partial dominant effect, and five with overdominant effect were observed. In the XZV hybrid, a total of 22 QTLs were detected in RILV, BCV, and MPH data sets (Table S2). Eleven QTLs were identified in more than two developmental stages or environments or populations. Interestingly, we observed one QTL *qBNP-Chr23-3* for BNP which were detected at all four time points. Totally, 12, 14, 13, and 9 QTLs, were respectively detected at stage *t*1, *t*2, *t*3, and *t*4 in three data sets. In backcross population, eleven QTLs with an additive effect, eight with partial dominant effect, and twelve with over dominant effect were observed.

Conditional QTL refers to the QTL expression at the specific stage from *t-1* to *t* at the locus^31^,^32^. QTL identified at *t* stage conditioned on *t-1* stage revealed net effects of QTL expression at the stage from *t-1* to *t* stage. A total of 34 and 14 conditional QTLs for BNP were identified for the adjacent stages in XZ and XZV hybrids respectively (Tables S3 and S4). In the XZ hybrid, nineteen, eight, and seven conditional QTLs were detected at Δ*t*1-2, Δ*t*2-3, and Δ*t*3-4 stages respectively; seven, five, and two conditional QTLs were respectively detected at Δ*t*1-2, Δ*t*2-3, and Δ*t*3-4 stages in the XZV hybrid. For MPH data set, eight, four, and three conditional QTLs were respectively found at Δ*t*1-2, Δ*t*2-3, and Δ*t*3-4 stages in two hybrids. These results indicated that QTL controlling BNP and heterosis might expressed more actively at the early stage. No conditional QTL and heterotic QTL (hQTL) was simultaneously identified during the entire stage of growth. Most of the conditional QTL detected at special stage showed that the QTL and hQTL expressed selectively at certain stage during reproductive growth.

### QTLs and QE interactions resolved by two-locus analyses

A total of 110 and 70 M-QTLs and QEs were respectively detected by inclusive composite interval mapping (ICIM) at four developmental stages of XZ and XZV hybrids (Table 5, S5, S6 and S7). In the XZ hybrid, a total of 57, 36 and 17 M-QTLs and QEs were detected in the RILs, BC hybrids, and MPH data at all of developmental stages, respectively. On average, the M-QTL explained 2.52%, 1.84%, and 1.08% of the phenotype variation (PV), and the QEs explained 0.51%, 0.73%, and 1.32% of the phenotype variation in the RILs, BC hybrids, and MPH data respectively. In the XZV hybrid, a total of 26, 21 and 23 M-QTLs and QEs were respectively detected in the RILVs, BCV hybrids, and MPH data at all of developmental stages. On average, the M-QTL explained 2.86%, 1.43%, and 1.87% of the phenotype variation, and the QEs explained 0.75%, 1.29%, and 0.80% of the phenotype variation in the RILVs, BCV hybrids, and MPH data, respectively.

Totally, at two-locus levels, 215 and 393 E-QTLs and QEs were detected by ICIM in three data sets of XZ and XZV hybrids at four developmental stages respectively (Table 5, S8, S9 and S10). In the XZ hybrid, a total of 145, 54, and 16 E-QTLs and QEs were detected in the RILs, BC hybrids, and MPH data, respectively. On average, the E-QTL explained 3.02%, 2.88%, and 1.78% of the phenotype variation, and the QEs explained 0.60%, 0.91%, and 1.56% of the phenotype variation in the RILs, BC hybrids, and MPH data respectively. In the XZV hybrid, a total of 288, 40 and 65 E-QTLs and QEs were detected in the RILVs, BCV hybrids, and MPH data, respectively. On average, the E-QTL explained 3.44%, 1.61%, and 2.40% of the phenotype variation, and the QEs explained 0.64%, 1.70%, and 1.19% of the phenotype variation in the RILVs, BCV hybrids, and MPH data respectively.

## Discussion

Yield is an important breeding target in Upland cotton. Biologically, plant height equals to all of internodes’ lengths above the ground, showing the rate of vegetative growth in Upland cotton^31^. However, boll number per plant equals to sum of all of boll numbers from the first boll development, reflecting the rate of reproductive growth in Upland cotton. Significant high positive correlations between boll number per plant and yield performance were observed for four developmental stages in three environments (Table S11). Early fruit number has indication of BNP at maturation. Meanwhile, BNP trait as yield components shows the best contribution to the yield (Table S11). Dynamic traits varied considerably among individuals in two hybrids. The incremental value of RIL and BC populations at early developmental stages were more than that at the final stage. The incremental values of the parents, RIL and BC populations displayed a dynamical developmental process during plant growth. The MPH (%) of backcross population gradually reduces with the plant growth, and heterosis performance showed time dependent and dynamic character. The BNP dynamic performance was conducive for studying the dynamics of heterosis in Upland cotton.

The genetic control underlying the dynamic heterosis is poorly understood in crop. The hQTL mapping provided information of cumulative effects at special stage in previous studies^5^,^35^. Based on final value of a quantitative trait, the genetic effects of QTLs at different developmental stages were overlooked^31^,^32^,^36^. In present research, a total of 14 and 9 QTLs for BNP were identified at maturity in XZ and XZV hybrids, while 60 and 39 QTLs were identified across other three development stages other than maturity respectively. It is obvious that lot of hQTLs identified at early stage were not identified at maturity stage (Table 5). Similar results were observed for the developmental behavior of tiller number in rice^37^ and plant height in Upland cotton^31^. Moreover, our results showed that the QTL and hQTL possessed temporal character^30^,^31^,^38^,^39^. At the single-locus level, the most number of QTLs was detected at *t*2 stage, and the *t*2 stage was the period of rapid reproductive stages. This phenomenon was supported by the result that the most incremental values and heterosis of BC population were observed at *t*1-*t*2 stages. In addition, the most number of conditional QTL and hQTL detected at *t*1-2 stage also verified this phenomenon. It is possible that more genes controlling heterosis of BNP trait largely expressed at the period of rapid reproductive stage in two hybrids. These results mean that the *t*1-*t*2 stage might be a more reasonable time for fine mapping of the hQTL.

Several QTLs at *t*1 and *t*4 stages in the MPH data set overlapped with QTLs in the RIL(V) and BC(V) populations (Table S12). At *t*2 and *t*3 stages, few QTLs were identified in the MPH data set that were also observed in the RIL(V) and BC(V) populations (Table S12). Previous studies have suggested that hQTLs of yield-related traits were independent and were different between QTLs and hQTL^22^,^40^,^41^. While, a recent study showed that many QTLs detected for grain yield in the MPH data set overlapped with QTL in the IF_2_ population and hQTLs were not independent^42^. In present study, we observe that hQTLs were not independent at the early and maturity stages, however, hQTLs were independent at rapid growth stage of *t*2 and *t*3. It suggested that phenotypes and heterotic traits might be controlled by different loci at different developmental stages in Upland cotton. In addition, we observed that the QTL *qBNP-Chr23-3* which showed high LOD value and phenotype variation at early stage can be repeatedly identified at four stages in multiple populations and environments in the XZV hybrid. Like the results from dynamic QTL for plant height at different developmental stages of rapeseed, we might predict this QTL at early developmental stages with no need for measurement of BNP trait at maturity stage^43^. These results added additional information and supplied important reference on QTL mapping for BNP and heterosis performance at different developmental stages.

Permanent genetic population such as RIL and permanent BC populations is required in order to detect convincing QTLs in more than one population or environment^4^. Using RIL and BC populations could mutually increase the power of detecting QTLs^34^. The RIL population appears to be able to detect more QTLs than BC population in the XZ hybrid (Table S2). Similar results were available in previous studies in rice^34^ and oilseed rape^10^. In the XZV hybrid, the opposite happened. In present study, the QTLs detected with the MPH data were relatively fewer than that of the QTLs identified with the RIL(V)s and BC(V) data. The reason may be that QTLs with an intermediate mode of inheritance and the QTL with lacking dominance effect could not be identified in the MPH data^10^. In previous study, three QTLs for BNP were identified in F_2:3_ and F_2:4_ populations derived from Upland cotton cross ‘GX1135’ × ‘GX100-2’. Present RIL population of F_9_ generation consisting 177 RILs along with corresponding backcross population were developed from F_2: 3_ population by single seed descent^17^. These three QTLs for BNP identified previously were once again detected simultaneously using RIL and backcross population. A large number of same QTLs between XZ and XZV hybrids were not detected. One possible reason is that the density of genetic map of the XZV hybrid is low, compared with the XZ hybrid. A total of 21 and 11 stable QTLs were identified in more than two developmental stages or environments or populations in XZ and XZV hybrids respectively. Specially, *qBNP-Chr23-3* could be detected across four developmental stages in the XZV hybrid, and the QTLs in different developmental stages showed same direction of genetic effect. These stable QTLs might be important for marker-assisted selection (MAS) for developing varieties with high BNP and yield in Upland cotton. Pyramiding the stable targeted loci is an efficient and feasible strategy for improving BNP and yield in Upland cotton.

The QTL identified simultaneously with RIL(V)s, BC(V) and MPH data allowed an assessment of the degree of dominance in two hybrids at the single-locus level^10^. In the XZV hybrid, a total of one (25.0%) QTLs for a PD effect and three (75.0%) QTLs for an OD effect were identified at *t*1 stage. At final *t*4 stage, a total of two (100.0%) QTLs for a PD effect and zero (0.00%) QTLs for an OD effect were identified. It is obvious that overdominance played a relatively more important role in controlling heterosis than partial dominance at early stage, while the result was opposite at final maturation stage. The study of maize heterosis showed that QTL for trait with low heterosis mainly existed in the additive to dominance range and QTL for trait with high heterosis had effects with the dominance to overdominance range^9^. Grain yield with the highest level of heterosis has the largest number of QTL exhibiting overdominance in all traits measured in rapeseed heterotic study^10^. In our study, BNP trait show the high level of heterosis in *t*1 and *t*2 stage, and the largest number of QTL exhibiting overdominance in all traits measured in rapeseed heterotic study. These results showed that dynamic dominance and overdominance played a role in controlling the expression of heterosis during growth in Upland cotton.

In XZ and XZV hybrids, a host of epistatic interactions and QEs were observed with the three data sets at four developmental stages. On average, the variation explained by E-QTL for most of stages was much greater than that by M-QTL in three data sets (Table 5). These results suggest that universal epistasis played an important role not only in the variation of the performance of the RIL(V) population but also in the expression of heterosis in BC(V) population^10^,^34^. Epistasis as the genetic basis of heterosis was supported by a series of previous studies^17^,^33^,^40^,^44^,^45^. In addition, QEs of M-QTL and QQEs of E-QTL in BC(V) population were much greater than that in RIL(V) population, showing that the backcross population was more susceptible to environment than RIL population. Recently, heterosis study of maize revealed that hybrids had a significant but moderate association between sensitivity to the environment and mean genotype value, whereas inbred lines did not show association^46^. That is why hybrid has superior performance than the parental lines in bad environment. Moreover, the result that QEs (QQEs) of M-QTL and E-QTL at early stages were much greater than that at final stage was found. It indicated that genotype environment interaction was dynamic at various developmental stages and heterosis performance was more susceptible to environment at early stage. Therefore, genotype by environment interaction was important for the stability of hQTL, and it should be taken into account in plant breeding programs^47^.

In present study, we find that the genetic effect of BNP heterosis is dynamic and complicated. It is involved in the different numbers of loci, dominance effect, epistatic interactions and QTL by environmental interactions at different developmental stages.

## Materials and Methods

### Plant materials and population construction

Two Upland cotton hybrids were employed in the present research. One is ‘Xinza 1’ (*G. hirsutum*)^31^,^48^,^49^ (hereinafter referred to as ‘XZ hybrid’) derived from a cross of ‘GX1135’ and ‘GX100-2’. The other one has common female parent with ‘Xinza 1’, derived from cross between ‘GX1135’ and ‘VGX100-2’^17^,^50^ (hereinafter referred to as ‘XZV’ hybrid). ‘VGX100-2’ was selected from ‘GX100-2’ and has significantly different performance compared with ‘GX100-2’.

Totally, four populations were used based on experimental design. The first population was an RIL population. It was developed from the F_1_ of ‘Xinza 1’^31^,^49^. The second population was an RILV population from the XZV hybrid. One hundred and eighty RILs were developed through 10 consecutive selfing generations^17^,^50^. The third population was a backcross developed from RIL population of the XZ hybrid. One hundred and seventy-seven BC hybrids, each hybrid was obtained from a cross where one RIL was used as the female parent and the common parent, ‘GX1135’, was used as male parent, respectively^51^. The fourth population was another backcross population (XZV). One hundred and eighty BC hybrids were developed from crosses between RILs from the RILV population used as the female parent and the common parent, GX1135, was used as male parent respectively^17^,^50^.

Two commercial hybrids of Upland cotton (*G. hirsutum*) were used as control respectively^17^. The F_1_ hybrid ‘Ruiza 816’ was used as control (CK_1_) at Handan (E1) and Cangzhou (E2), Hebei Province. The F_1_ hybrid “Ezamian 10” was used as control (CK_2_) at location Xiangyang (E3), Hubei Province. Additionally, two special plots, each consisting of two rows of the XZ hybrid, and its parents ‘GX1135’ and ‘GX100-2’ respectively, were used as controls in the experiments of population 1 and population 3. Similar controls were set for the experiments of population 2 and population 4; each plot consisting of the XZV hybrid F_1_, and its parents ‘GX1135’ and ‘VGX100-2’.

### Field arrangement and trait evaluation

The four populations and controls were planted at three locations (E1: Handan, E2: Cangzhou, Hebei Province; E3: Xiangyang, Hubei Province) in 2012. In experiment of population 1 and population 2, two-row plots with each plot were used. However in experiment of population 3 and population 4, six-row plots with each plot consisting of two rows of BC hybrid [RIL(V) × GX1135] in the middle, and two rows in both sides for the corresponding parents: one side two rows for the corresponding RIL(V)′, and the other two rows for ‘GX1135’. Each line in RIL(V)′ population was used as the female parent in BC(V) population and was the same one in RIL(V) population. For ease of description, we will refer to the RIL(V)s in BC(V) population as RIL(V)′ population, respectively. So in both population 3 and 4, the female were marked as RIL(V)′, respectively.

The field planting followed a randomized complete block design with two replications at each location. Field management followed the local conventional standard field practices. Boll samples of eight typical plants were measured for boll number per plant (BNP) at four time points, respectively (*t*1: August 1th, *t*2: August 15th, *t*3: August 30th, and *t*4: September 15th). The data for different stages of *t*1, *t*2, *t3* and *t4* were used for QTL mapping. The incremental values in interval time Δ*t*1-2, Δ*t*2-3 and Δ*t*3-4 were used to map conditional QTL.

### DNA isolation, genotype analysis and linkage map construction

Young leaves were collected from labeled parents and two RIL individuals. Extraction of individual genomic DNA and population genotype analysis were carried out following the methods of Liang *et al.*^48^. A total of 48,836 pairs of SSR primer were used to screen polymorphic loci between three parents. Totally, 653 polymorphic loci for the XZ hybrid and 382 polymorphic loci for the XZV hybrid were acquired and used to conduct genotype analysis of population. MAPMAKER 3.0 was used to construct genetic linkage map using the *Kosambi* mapping function^52^.

### Data analysis

For the RIL(V) and RIL(V)′ populations, the mean trait values from two replications were used as raw data at each location. For each of the BC(V) populations, the mean trait values and mid-parental heterosis (MPH) of the BC(V) were used independently as raw data^4^ at three locations. Mid-parent heterosis (MPH) of each BC(V) was calculated as follows: MPH = F_1_ **−** MP, where F_1_ was the mean trait value of the BC(V), and MP = [RIL(V)′ + GX1135]/2 were the mid-parental trait values of the corresponding female RIL(V)′ and the recurrent parent. The genotype for each BC(V) was deduced according to the RIL(V)′s genotype used as the parent for cross. Single-locus QTL and conditional QTL were conducted using composite interval mapping^53^ by QTL Cartographer V2.5 software (http://statgen.ncsu.edu/qtlcart/WQTLCart.htm) in RIL(V)′, RIL(V), BC(V) and MPH data. A stringent LOD threshold of 3.0 was used to declare suggestive QTL^17^,^48^. The graphic representation of the linkage group and QTL marked were created by Map Chart 2.2^54^. QTL nomenclature used in rice was employed^55^. At the single-locus level, the genetic effects in BC were defined as follows: a = (P1P1 − P2P2)/2; MPH = d = (BC − (P1P1 + P2P2)/2) and BC = (a + d) (P1 is the recurrent parent). QTL detected only in RIL or BC and not for MPH were referred to as additive. QTLs with d/a ≤ 1 were considered as being complete or partial dominant loci. QTLs with d/a > 1 or only detectable for MPH data were considered as over-dominant loci^10^. Two-locus analysis that tests the main-effect QTL (M-QTL), and digenic epistatic QTL (E-QTL) and their environmental interactions (QTL × environment, QE), was conducted using three data sets by the software ICIMapping 4.0 (http://www.isbreeding.net/). LOD thresholds were set at 2.5 and 5.0 for declaring the presence of M-QTLs and E-QTLs mapping, respectively, same as Shang *et al.*^17^. Basic statistical analysis was conducted by the software SPSS version 19.0 (SPSS, Chicago, USA).

## Additional Information

**How to cite this article**: Shang, L. *et al.* Genetic analysis of Upland cotton dynamic heterosis for boll number per plant at multiple developmental stages. *Sci. Rep.* **6**, 35515; doi: 10.1038/srep35515 (2016).

## Supplementary Material

Supplementary Information

## Figures and Tables

**Table 1:** Summary statistics on boll number per plant in two hybrids.

Stage	E^a^	Mean				Min			Max			Parents		F_1_	CK^b^
		RIL	BC	MPH	MPH(%)	RIL	BC	MPH	RIL	BC	MPH	♀	♂		
XZ hybrid															
* t*1	E1	2.05	3.40	0.35	11.48	0.31	1.13	−2.09	5.00	6.13	2.72	3.50	2.19	3.84*^c^	4.03
	E2	2.35	2.78	0.28	11.20	0.38	1.44	−1.28	6.69	5.56	2.00	2.78	2.42	3.39*	3.38
	E3	0.81	1.43	0.40	38.83	0.00	0.25	−0.75	4.38	3.06	2.13	1.38	0.38	—d	0.38
* t*2	E1	13.67	16.53	0.76	4.82	5.63	10.13	−5.31	20.38	22.91	5.18	16.81	13.81	17.04	13.53
	E2	13.76	15.49	0.83	5.66	4.88	10.88	−3.88	20.69	20.75	4.72	17.02	13.53	16.51*	15.91
	E3	4.76	6.29	0.92	17.13	1.75	3.19	−1.63	9.38	9.06	3.78	6.16	3.47	7.53**	7.50
* t*3	E1	17.22	18.99	0.72	3.94	7.44	12.69	−4.75	25.56	24.50	7.91	17.33	15.13	20.13*	15.31
	E2	19.85	22.18	1.21	5.77	10.94	16.50	−5.72	28.13	28.63	7.75	23.10	20.58	24.69**	18.97
	E3	15.72	18.50	0.92	5.23	9.44	12.81	−3.78	23.69	25.63	4.66	17.53	14.75	20.45*	25.50
* t*4	E1	16.50	18.96	0.80	4.41	7.25	12.60	−3.81	28.06	25.00	8.03	17.83	16.56	20.88*	16.28
	E2	20.63	23.90	1.25	5.52	11.63	19.00	−4.94	27.06	29.31	6.16	23.58	20.48	25.36*	20.34
	E3	21.16	21.25	0.61	2.96	14.06	15.38	−7.03	30.75	29.50	7.14	20.38	17.66	21.83*	22.25
XZV hybrid															
* t*1	E1	1.91	3.44	0.68	24.64	0.19	1.25	−1.41	4.63	5.44	2.91	2.63	1.22	3.34**	3.94
	E2	3.13	4.27	0.94	28.23	0.54	2.38	−1.37	7.06	6.50	3.22	3.86	2.53	4.69**	5.16
	E3	0.63	0.68	0.18	36.00	0.00	0.00	−0.84	2.38	2.44	1.69	1.19	0.81	d	1.38
* t*2	E1	11.30	14.26	1.98	16.12	4.38	9.13	−2.16	19.63	19.81	7.16	13.28	9.67	15.67**	13.81
	E2	14.56	18.38	2.72	17.37	5.00	12.50	−3.91	26.56	24.00	8.69	17.70	13.08	20.82**	16.69
	E3	3.63	5.03	1.48	41.69	0.44	1.88	−2.63	9.19	10.13	6.75	3.03	1.86	4.94**	7.56
* t*3	E1	14.14	17.79	2.39	15.52	6.84	12.69	−1.75	25.00	23.19	6.72	15.44	13.65	19.33**	14.44
	E2	18.75	22.54	3.30	17.15	5.43	17.75	−3.66	28.69	29.44	11.63	22.98	16.82	24.96**	19.22
	E3	12.67	16.99	3.83	29.10	3.75	11.25	−3.50	25.50	22.94	12.38	10.80	8.95	16.70**	21.19
* t*4	E1	15.08	17.68	2.19	14.14	8.50	12.13	−2.53	21.56	23.69	7.97	15.54	14.39	19.47**	15.75
	E2	20.32	24.71	3.49	16.45	7.18	18.69	−3.38	32.63	31.69	12.47	25.17	17.81	26.14**	21.34
	E3	15.81	20.97	4.27	25.57	4.89	15.13	−5.31	29.13	29.56	13.61	13.22	11.47	20.61**	20.66

^a^E, E1, Handan; E2, Cangzhou; E3, Xiangyang.

^b^CK, the CK1 is ‘Ruiza 816’ at E1 and E2; The CK2 is’ Ezamian 10’ at E3.

^c^Comparison between the mean of parent and F_1_; *, **indicate that the correlation is significant at 0.05 and 0.01 probability levels, respectively.

^d^missing data.

**Table 2:** Correlations between RIL, BC, and MPH data in two hybrids.

Stage	Env.	Between RILs and BC		Between RILs and MPH		Between BC and MPH	
		XZ	XZV	XZ	XZV	XZ	XZV
*t*1	E1	0.34**	0.26**	−0.14	−0.27**	0.70**	0.69**
	E2	0.33**	0.45**	0.01	−0.09	0.68**	0.71**
	E3	0.52**	0.36**	−0.10	0.15*	0.51**	0.85**
*t*2	E1	0.07	0.40**	−0.24**	−0.32**	0.63**	0.52**
	E2	0.16*	0.52**	−0.15	−0.24**	0.72**	0.52**
	E3	0.30**	0.31**	−0.09	−0.25**	0.73**	0.68**
*t*3	E1	0.16*	0.38**	−0.17*	−0.27**	0.62**	0.49**
	E2	0.33**	0.29**	0.01	−0.39**	0.68**	0.55**
	E3	0.52**	0.21**	−0.10	−0.71**	0.51**	0.40**
*t*4	E1	0.07	0.36**	−0.24**	−0.22**	0.63**	0.52**
	E2	0.16*	0.22**	−0.15	−0.44**	0.72**	0.57**
	E3	0.30**	0.25**	−0.09	−0.74**	0.73**	0.32**

*,**indicate that the correlation is significant at 0.05 and 0.01 probability levels, respectively See Table 1 for other abbreviations.

**Table 3:** Correlation coefficients between genotypic heterozygosity and dynamic trait performance in BC and MPH data sets.

Stage	BC						MPH					
	E1		E2		E3		E1		E2		E3	
	XZ	XZV	XZ	XZV	XZ	XZV	XZ	XZV	XZ	XZV	XZ	XZV
*t*1	0.15*	0.00	0.13	0.03	0.09	−0.09	0.11	0.11	0.09	0.03	0.15*	−0.02
*t*2	0.03	0.06	0.05	−0.05	0.00	0.07	0.01	0.16*	0.00	−0.02	0.12	0.15*
*t*3	0.00	0.14	0.08	−0.01	−0.03	0.06	0.02	0.21**	0.07	0.00	−0.03	0.13
*t*4	0.00	0.11	0.11	0.00	0.02	−0.03	0.00	0.19*	0.04	0.00	−0.03	0.07

See Table 1 for footnotes.

**Table 4:** Effects of QTLs identified for boll number per plant by composite interval mapping in two backcross populations.

Trait	XZ hybrid				XZV hybrid			
	A	PD	OD	Sum	A	PD	OD	Sum
*t*1	3	2	4	9	2	1	3	6
*t*2	10	0	3	13	3	2	5	10
*t*3	3	2	2	7	2	3	4	9
*t*4	5	1	2	8	4	2	0	6
Total	21	5	11	37	11	8	12	31

A, additive effect; PD, partial dominant effect; OD, over-dominant effect.

**Table 5:** Summary of M-QTLs and E-QTLs detected by inclusive composite interval mapping.

Stage	RIL			BC			MPH			RILV			BCV			MPH		
M-QTL	n	P(A)	P(AE)	n	P(A)	P(AE)	n	P(A)	P(AE)	n	P(A)	P(AE)	n	P(A)	P(AE)	n	P(A)	P(AE)
*t*1	12	2.91	0.45	8	1.96	0.38	5	1.41	1.34	8	2.53	0.82	7	1.97	0.83	7	1.05	0.57
*t*2	15	2.36	0.66	12	1.63	0.88	5	0.97	1.53	5	3.86	0.87	9	1.64	1.12	6	1.46	1.33
*t*3	17	2.60	0.43	8	1.64	1.10	3	0.25	1.78	6	2.90	0.64	2	1.64	1.38	4	2.46	0.74
*t*4	13	2.22	0.49	8	2.11	0.56	4	1.69	0.63	7	2.17	0.67	3	0.47	1.84	6	2.51	0.58
Mean	14.3	2.52	0.51	9.0	1.84	0.73	4.3	1.08	1.32	6.5	2.86	0.75	5.3	1.43	1.29	5.8	1.87	0.80
E-QTL	n	P(AA)	P(AAE)	n	P(AA)	P(AAE)	n	P(AA)	P(AAE)	n	P(AA)	P(AAE)	n	P(AA)	P(AAE)	n	P(AA)	P(AAE)
*t*1	42	2.32	0.72	19	2.55	1.16	1	0.03	2.20	89	2.74	0.82	17	2.17	1.24	12	2.24	1.28
*t*2	63	2.59	0.79	22	2.73	0.95	8	1.43	1.89	62	3.33	0.54	20	2.36	1.03	8	2.01	1.82
*t*3	25	3.98	0.24	6	3.11	0.58	4	2.34	1.43	70	4.15	0.43	1	1.31	2.16	21	2.84	0.75
*t*4	15	3.18	0.64	7	3.12	0.96	3	3.33	0.73	67	3.53	0.78	2	0.60	2.39	24	2.51	0.92
Mean	36.3	3.02	0.60	13.5	2.88	0.91	4.0	1.78	1.56	72.0	3.44	0.64	10.0	1.61	1.70	16.3	2.40	1.19

n: the number of QTLs identified; P: (in %) was the mean of trait phenotypic variances explained by a single M-QTL or E-QTL.
