# Supplementary material for: Genetic analysis of Upland cotton dynamic heterosis for boll number per plant at multiple developmental stages
Source: Sci Rep. 2016 Oct 17;6:35515. doi: 10.1038/srep35515 (PMC5066282; doi:10.1038/srep35515)
Supplement: Supplementary Information [file srep35515-s1.pdf]

Genetic analysis of Upland cotton dynamic heterosis for boll number per plant at multiple developmental stages

Lianguang Shang[1], Yumei Wang[2], Shihu Cai[1], Lingling Ma[1], Fang Liu[3], Zhiwen Chen[1], Ying Su[1], Kunbo Wang[3], Jinping Hua[1]*

Table S1 The results of ANOVA of boll number per plant

Stage	Source of variation	XZ hybrid			XZV hybrid		
		RIL	BCF_1	MPH	RIL	BC	MPH
		MS	MS	MS	MS	MS	MS
*t*1	G	3.52***	1.76***	1.01***	3.98***	1.56***	1.44***
	E	236.96***	358.67***	1.32	561.81***	1246.92***	53.25***
	G × E	0.93***	0.87**	0.84**	0.98***	0.83	0.92***
	error	0.40	0.66	0.64	0.42	0.78	0.68
t2	G	20.86***	9.40***	5.40	47.55***	11.62***	9.91***
	E	9461.90***	11178.20***	2.25	11268.96***	16515.56***	138.08***
	G × E	5.15	4.68	4.77	8.03***	5.03**	5.50*
	error	4.80	4.47	4.59	3.90	3.93	4.49
*t*3	G	36.56***	14.96***	9.25**	98.26***	11.47***	22.76***
	E	1548.03***	1411.14***	21.43*	3601.04***	3194.40***	191.38***
	G × E	9.06***	8.06	7.69	14.79***	8.46*	11.10***
	error	6.90	8.34	6.99	9.60	7.05	8.12
*t*4	G	33.35***	16.11***	11.74*	95.00***	11.96***	25.53***
	E	2303.14***	2152.98***	37.36*	2884.48***	4371.52***	394.27***
	G × E	12.05***	9.11	10.10	20.72***	9.81*	12.52***
	error	8.38	10.04	9.22	8.86	7.86	9.00

G: Genotype, E: Environment, G × E: Genotype × Environment, Significance is shown at *P=0.05, **P=0.01,***P=0.001, respectively.

Table S2 QTLs identified for boll number per plant by composite interval mapping in two hybrids

QTL	Stage	Env	Marker interval		RIL			BC			MPH			d/a	Type
					LOD	A	Var%	LOD	A+D	Var%	LOD	D	Var%		
XZ hybrid															
qBNP-Chr1-1	*t*2	E3	SWU10930	ICR03295				3.53	-0.34	7.13					A
qBNP-Chr1-2	*t*2	E3	CGR6129	DPL0790				4.68	-0.39	9.75					A
qBNP-Chr1-3	*t*1	E1	NAU3177	ICR03724	3.00	-0.27	6.04								
	*t*2	E2	NAU3177	ICR03724	3.01	-0.70	7.04								
	*t*2	E1	ICR03724	ICR03725	2.65	-0.60	5.46								
	*t*3	E2	ICR03724	ICR03725	3.46	-0.85	7.33								
qBNP-Chr1-4	*t*4	E2	SWU10986	NAU2218	3.25	-0.80	7.31								
	*t*3	E2	SWU10986	NAU2218	4.81	-1.11	12.80								
	*t*3	E3	SWU10986	NAU2218				3.01	-0.72	8.62		(±0.39)		0.35	PD
qBNP-Chr1-5	*t*2	E2	CGR5663	NAU2343				3.53	-0.57	7.52					A
	*t*3	E2	CGR5663	NAU2343				4.96	-0.81	11.82					A
	*t*4	E2	CGR5663	NAU2343				2.77	-0.59	6.12					A
qBNP-Chr1-6	*t*4	E1	SWU10994	HAU1001	3.02	-0.79	6.27								
	*t*2	E2	SWU10994	HAU1001	2.94	-0.69	7.04								
	*t*3	E2	SWU10994	HAU1001	3.45	-0.86	7.88								
qBNP-Chr1-7	*t*4	E3	ICR11883	CGR6356				2.88	-0.69	6.36					A
	*t*4	E1	CGR6356	SWU11632				2.80	-0.53	6.04					A
qBNP-Chr2-1	*t*2	E3	SWU11889	SWU11887	13.58	0.79	23.07								
	*t*1	E3	SWU11889	SWU11887	8.91	0.30	17.87								
qBNP-Chr2-2	*t*1	E2	SWU11887	SWU11976	4.65	0.30	8.12								
	*t*1	E3	SWU11887	SWU11976				3.82	0.16	7.85		(±0.14)		0.47	PD
	*t*3	E3	SWU11887	SWU11976	6.92	1.02	13.10								
	*t*1	E1	SWU11887	SWU11976	6.07	0.40	14.63								
	*t*1	E1	SWU11887	SWU11976							4.31	-0.29	11.46	0.73	PD
qBNP-Chr6-1	*t*2	E3	ICR00143	CGR5108							6.28	0.60	31.83		OD
	*t*1	E3	ICR00143	CGR5108				2.55	0.25	17.08					A
qBNP-Chr6-2	*t*1	E3	NAU896	BNL3650		(±0.09)		3.77	-0.22	11.92					
	*t*1	E3	BNL3650	ICR10602							2.59	-0.13	5.28	1.44	OD
qBNP-Chr7-1	*t*2	E2	SWU10064	NAU3181							3.23	0.52	9.19		OD
qBNP-Chr7-2	*t*3	E1	HAU1483b	Gh145	3.14	0.81	6.21								
qBNP-Chr11-1	*t*1	E3	NAU5428	Gh256							3.16	-0.18	11.01		OD
qBNP-Chr11-2	*t*3	E2	CER0098	CGR5421							2.98	-0.94	18.88		OD
	*t*4	E2	CER0098	CGR5421							2.59	-0.83	14.11		OD
	*t*4	E3	CER0098	CGR5421							2.52	-0.81	9.64		OD
	*t*4	E3	CGR5421	ICR08245		(±0.01)		2.88	-0.82	9.30					
qBNP-Chr11-3	*t*4	E2	SWU15972	TMB0628							3.08	-0.75	11.55	0.72	PD

QTL	Stage	Env	Marker interval		RIL			BC			MPH			d/a	Type
					LOD	A	Var%	LOD	A+D	Var%	LOD	D	Var%		
	*t*4	E3	SWU15972	TMB0628	2.52	-1.04	10.06								
	*t*1	E3	SWU15972	TMB0628	2.70	-0.15	4.93								
qBNP-Chr11-4	*t*2	E3	SWU12282	Gh316	5.69	-0.51	8.99								
qBNP-Chr12-1	*t*4	E2	HAU1316	NAU3519	4.02	-0.97	10.81								
qBNP-Chr14-1	*t*3	E1	Gh120	PGML1884				3.11	0.53	6.51					A
	*t*4	E1	Gh120	PGML1884				3.12	0.55	6.55					A
qBNP-Chr16-1	*t*2	E3	PGML1309	Gh137				3.12	-0.30	5.91					A
qBNP-Chr16-2	*t*1	E2	DPL0897	BNL1026							3.44	0.17	7.72		OD
qBNP-Chr16-3	*t*1	E2	SWU10038	ICR00016	3.37	-0.26	5.82								
qBNP-Chr21-1	*t*2	E1	SWU16487	SWU16488				2.75	-0.48	6.00					A
	*t*3	E1	SWU16487	SWU16488							2.59	-0.52	5.62		OD
qBNP-Chr21-2	*t*1	E1	JESPR154	SWU14431a	4.09	-0.30	7.56								
	*t*1	E2	JESPR154	SWU14431a	3.90	-0.29	7.26								
qBNP-Chr21-3	*t*1	E1	SWU15915	SWU0189	5.65	-0.34	10.38								
	*t*1	E2	SWU15915	SWU0189	6.72	-0.38	12.27								
	*t*2	E3	SWU15915	SWU0189	3.35	-0.37	5.02								
	*t*3	E3	SWU15915	SWU0189	5.18	-1.39	9.96								
qBNP-Chr21-4	*t*1	E3	SWU0189	DPL0050a	3.16	-0.17	6.02								
	*t*1	E1	SWU0189	DPL0050a	4.21	-0.34	10.06								
qBNP-Chr21-5	*t*2	E1	CGR5808	HAU0423	4.23	-0.89	12.05								
	*t*2	E2	HAU0423	CGR5806	3.30	-0.78	9.18								
	*t*3	E1	CGR5808	HAU0423	3.12	-0.93	8.25								
	*t*3	E1	HAU0423	CGR5806				2.78	-0.65	9.02		(±0.28)		0.3	PD
qBNP-Chr21-6	*t*2	E2	CGR5806	DPL0777				5.95	-0.94	19.69					A
	*t*1	E2	CGR5806	DPL0777				3.18	-0.32	16.91					A
qBNP-Chr21-7	*t*3	E1	CGR5217	BNL3442a				4.44	0.67	9.45					A
qBNP-Chr22-1	*t*2	E2	SWU21635	SWU21646	2.84	-0.61	5.54								
	*t*3	E2	SWU21646	SWU21585	3.15	-0.76	6.08								
qBNP-Chr24-1	*t*1	E3	Gh298	SWU13133							3.75	0.15	7.69		OD
qBNP-Chr24-2	*t*2	E3	SWU13150	PGML4657				3.62	0.37	8.13					A
	*t*2	E2	PGML4657	Gh454				2.97	0.51	5.62					A
qBNP-Chr24-3	*t*4	E3	Gh268	SWU13268				3.68	0.74	7.47					A
	*t*2	E3	PGML3120	SWU13256				6.33	0.45	12.41					A
qBNP-Chr24-4	*t*1	E2	SWU13267	BNL1521				3.63	0.25	10.94					A
qBNP-Chr24-5	*t*4	E2	SWU13758	CGR5423	4.57	0.87	8.97								
qBNP-Chr26-1	*t*3	E1	HAU1571	BNL598	3.82	0.91	7.79								
qBNP-Chr26-2	*t*2	E2	SWU17233	SWU17251							4.55	0.55	9.79		
qBNP-Chr26-3	*t*2	E2	C2_0135	PGML2321				3.96	-0.61	8.15					OD

QTL	Stage	Env	Marker interval		RIL			BC			MPH			d/a	Type
					LOD	A	Var%	LOD	A+D	Var%	LOD	D	Var%		
							XZV hybrid								
qBNP-Chr1-1	*t*2	E1	ICR03725	ICR03724							3.84	-0.63	11.08		OD
qBNP-Chr2-1	*t*2	E3	CGR6695	SWU11013							3.65	-0.37	7.30		OD
	*t*3	E3	CGR6695	SWU11013							4.07	-1.01	8.39		OD
qBNP-Chr2-2	*t*4	E2	SWU11976	SWU12001				3.00	-0.68	7.59					A
qBNP-Chr3-1	*t*2	E1	SWU12840	NAU2742							5.98	-0.92	16.56		OD
qBNP-Chr10-1	*t*4	E3	NAU2139	TMB1152				3.72	-1.00	16.52					A
qBNP-Chr12-1	*t*1	E2	Gh631	COT107				2.67	-0.22	5.87					
	*t*1	E2	Gh631	COT107		(±0.03)					2.85	-0.25	7.74	8.30	OD
qBNP-Chr16-1	*t*1	E3	HAU3081	NAU747				4.64	0.16	13.45					
	*t*1	E3	NAU747	HAU1129							3.53	0.16	13.69		OD
qBNP-Chr16-2	*t*1	E3	NAU747	HAU1129				5.61	0.20	20.74					A
qBNP-Chr16-3	*t*1	E2	SWU18366	SWU18579	3.83	-0.37	7.79								
	*t*2	E1	SWU10266	SWU18366				2.91	-0.52	7.79		(±0.53)		0.50	PD
	*t*2	E2	SWU18366	SWU18579	2.85	-1.05	5.72								
qBNP-Chr21-1	*t*2	E2	JESPR154	BNL5602							3.43	-0.68	8.02		OD
qBNP-Chr23-1	*t*2	E3	HAU1758	SHIN1076				3.30	-0.42	11.13					A
	*t*3	E1	SHIN1076	BNL3482							2.91	0.63	9.68		OD
qBNP-Chr23-2	*t*1	E2	MUSB994	NAU2238	2.69	-0.37	8.04								
	*t*3	E1	MUSB994	NAU2238				2.96	-0.61	9.16		(±0.64)		0.51	PD
	*t*3	E1	MUSB994	NAU2238	4.91	-1.25	16.00								
	*t*4	E1	MUSB994	NAU2238				3.35	-0.68	10.59					A
qBNP-Chr23-3	*t*1	E2	NAU2238	NAU3588				5.74	-0.32	12.76		(±0.12)		0.27	PD
	*t*1	E2	NAU3588	NAU5373a	5.80	-0.44	11.45								
	*t*2	E1	NAU3588	NAU5373a	5.01	-1.00	9.71								
	*t*2	E1	NAU3588	NAU5373a				3.59	-0.50	7.34		(±0.50)		0.50	PD
	*t*2	E2	NAU3588	NAU5373a	5.93	-1.51	11.93								
	*t*3	E1	NAU3588	NAU5373a				5.80	-0.71	12.16		(±0.48)		0.40	PD
	*t*3	E1	NAU3588	NAU5373a	7.00	-1.19	14.52								
	*t*3	E2	NAU3588	NAU5373a	2.68	-1.17	5.56								
	*t*4	E1	NAU2238	NAU3588	4.30	-0.83	10.05								
	*t*4	E1	NAU3588	NAU5373a				4.52	-0.64	9.63		(±0.19)		0.23	PD

QTL	Stage	Env	Marker interval		RIL			BC			MPH			d/a	Type
					LOD	A	Var%	LOD	A+D	Var%	LOD	D	Var%		
	*t*4	E2	NAU3588	NAU5373a	2.81	-1.19	5.94								
qBNP-Chr23-4	*t*3	E1	NAU5373b	HAU2648				5.01	-0.82	16.83					A
qBNP-Chr24-1	*t*1	E3	PGML1207	Gh54				4.00	0.16	13.88					A
qBNP-Chr24-2	*t*3	E2	Gh54	Gh454							3.92	0.92	9.13		OD
	*t*2	E3	Gh454	HAU3076				4.02	0.38	8.39					A
qBNP-Chr24-3	*t*3	E2	HAU3076	SWU13121							3.60	-0.87	8.02		OD
qBNP-Chr25-1	*t*1	E3	SWU19676	NAU2968	3.07	0.11	6.29								
qBNP-Chr25-2	*t*2	E2	NAU2968	DPL0377		(±0.40)					3.70	0.68	8.20	1.70	OD
	*t*2	E2	NAU2968	DPL0377				6.64	1.08	18.65					
qBNP-Chr25-3	*t*2	E2	SWU19413	SWU19431				7.96	1.01	16.19					A
qBNP-Chr26-1	*t*4	E1	DPL0057	NAU3109				2.78	-0.49	5.97					A
	*t*1	E2	NAU3109	CGR6772							2.65	0.28	9.60		OD
qBNP-Chr26-2	*t*3	E1	HAU0355	SWU16777				3.74	-0.61	8.95					A
	*t*3	E3	HAU0355	SWU16777							4.81	-1.26	11.27	0.69	PD
	*t*3	E3	SWU16777	SWU16780	4.14	1.82	8.74								
	*t*4	E3	SWU16777	SWU16780							2.87	-1.03	6.29	0.53	PD
	*t*4	E3	SWU16777	SWU16780	3.81	1.94	8.31								

Bold figures indicate the QTL was detected in more than one environments, stages or populations simultaneously

Env., environment, E1: Handan; E2: Cangzhou; E3: Xiangyang

Effect, the genetic expectation of a QTL effect obtained is the additive effect (A) when estimated from the RILs and RIL′s, the additive and dominance effects (A+D) from the BCF_1 mean values, and the dominance effect (D) from the MPH values

Var%, phenotypic variation explained by a single QTL

d/a, dominance ratio

Type, The QTLs which were identified in backcross population were grouped into three types: (A) additive effect, (PD) partial dominance effect, (OD) overdominance effect.

Table S3 Conditional QTL identified for boll number per plant of RIL(V) and BC(V) populations by composite interval mapping

QTL	Env	Stage	Marker interval		RIL			BC			MPH		
					LOD	A	Var%	LOD	A+D	Var%	LOD	D	Var%
			XZ hybrid										
qBNP-Chr1-1	E2	△ *t*1-2	SWU10912	DPL0090	3.22	-0.61	9.47						
	E2	△ *t*3-4	DPL0090	Gh398							3.85	0.51	8.51
qBNP-Chr1-2	E3	△ *t*1-2	CGR6129	DPL0790				4.72	-0.31	10.47			
qBNP-Chr1-4	E2	△ *t*1-2	CGR5663	NAU2343				3.54	-0.47	7.25			
	E2	△ *t*3-4	SWU0077	HAU1417	2.83	0.33	5.92						
qBNP-Chr1-5	E1	△ *t*2-3	ICR11883	CGR6356	3.14	0.40	6.48						
qBNP-Chr2-1	E3	△ *t*1-2	SWU11889	SWU11887	10.42	0.53	18.89						
qBNP-Chr2-2	E2	△ *t*2-3	Gh64	SWU17257	3.11	-0.65	13.15						
	E2	△ *t*1-2	SWU17233	SWU17251							4.75	0.53	10.25
qBNP-Chr5-1	E3	△ *t*2-3	SWU20917	NAU6240	3.27	-0.52	6.97						
	E3	△ *t*1-2	NAU6240	PGML1671	2.63	0.34	7.83						
qBNP-Chr5-2	E2	△ *t*3-4	CGR5025	NBRI0694	2.53	0.31	5.27						
	E2	△ *t*2-3	NBRI0694	DPL0022	3.13	-0.46	6.38						
qBNP-Chr5-3	E1	△ *t*1-2	SWU17787	SWU13378				3.08	-0.43	6.79			
	E1	△ *t*1-2	SWU17787	SWU13378	2.56	-0.46	5.00						
qBNP-Chr7-1	E2	△ *t*1-2	SWU10064	NAU3181							3.95	0.55	11.42
qBNP-Chr7-2	E1	△ *t*1-2	SWU10205	HAU1483a	3.51	0.62	9.21						
qBNP-Chr9-1	E2	△ *t*3-4	CGR5867	SWU15407				2.99	-0.38	6.45			
	E2	△ *t*3-4	NAU3052	NAU5474				4.05	-0.44	8.74			
qBNP-Chr11-1	E3	△ *t*1-2	SWU12282	Gh316	4.33	-0.35	7.64						
qBNP-Chr13-1	E3	△ *t*2-3	NAU2893	Gh157							4.07	0.46	8.85
qBNP-Chr13-2	E1	△ *t*1-2	Gh157	BNL1495							3.70	0.55	7.91
qBNP-Chr13-3	E1	△ *t*1-2	BNL1495	CGR5390							3.37	0.55	10.27
qBNP-Chr14-1	E3	△ *t*2-3	SWU14543	ICR12037	3.28	0.51	6.87						
qBNP-Chr14-2	E3	△ *t*1-2	ICR12037	CGR5675				5.00	0.38	14.98			
qBNP-Chr14-3	E3	△ *t*1-2	PGML1368	PGML1568				4.14	0.29	8.42			
qBNP-Chr14-4	E3	△ *t*3-4	CIR228	BNL2485							4.35	0.68	9.05
qBNP-Chr16-1	E3	△ *t*3-4	SWU10214	Gh56	4.62	-0.74	11.35						
qBNP-Chr16-2	E3	△ *t*2-3	SWU10037	ICR00010				4.00	0.58	8.09			
qBNP-Chr21-1	E1	△ *t*1-2	SWU16487	SWU16488							2.98	-0.38	6.34
	E2	△ *t*3-4	SWU16487	SWU16488				2.53	-0.41	7.28			
qBNP-Chr21-2	E3	△ *t*2-3	CGR5602	JESPR154				4.53	-0.67	9.19			
qBNP-Chr21-3	E3	△ *t*2-3	SWU14431a	SWU15915				4.18	-0.73	10.12			
qBNP-Chr21-4	E2	△ *t*1-2	CGR5806	DPL0777				3.85	-0.63	12.77			
qBNP-Chr22-1	E2	△ *t*1-2	SWU21646	SWU21585	4.48	-0.60	8.97						

qBNP-Chr24-1	E3	△ *t*3-4	CGR5202	Gh298							3.10	-0.57	6.30
qBNP-Chr24-2	E3	△ *t*1-2	PGML3120	SWU13256				4.78	0.31	9.70			
qBNP-Chr24-3	E3	△ *t*1-2	SWU13267	BNL1521				4.67	0.39	16.58			
qBNP-Chr25-1	E1	△ *t*1-2	SWU19763	SWU19129	3.32	0.62	9.05						
qBNP-Chr26-1	E1	△ *t*3-4	HAU1571	BNL598				3.34	-0.29	7.58			
qBNP-Chr26-2	E1	△ *t*3-4	SWU17467	SWU17419				4.28	-0.32	9.48			
qBNP-Chr26-3	E2	△ *t*1-2	PGML1833	SWU0514				3.20	-0.43	6.15			
				XZV hybrid									
qBNP-Chr1-1	E1	△ *t*1-2	ICR03725	ICR03724							2.64	-0.44	7.83
	E1	△ *t*1-2	ICR03725	ICR03724				2.83	-0.49	9.95			
qBNP-Chr2-1	E3	△ *t*1-2	DPL0217	CGR6695							3.09	-0.29	6.77
	E3	△ *t*2-3	CGR6695	SWU11013							2.58	-0.65	5.74
qBNP-Chr10-1	E2	△ *t*2-3	NAU4967	SWU19932							3.18	-0.93	19.67
qBNP-Chr12-1	E3	△ *t*1-2	HAU3373	CGR6847							3.33	-0.29	6.73
qBNP-Chr23-1	E1	△ *t*2-3	CGR5158	HAU1758				4.17	0.42	9.17			
	E2	△ *t*3-4	CGR5158	HAU1758	2.56	0.32	4.57						
qBNP-Chr23-2	E3	△ *t*3-4	Gh327	ICR06429	4.12	0.73	12.26						
qBNP-Chr23-3	E2	△ *t*3-4	NAU2140	DC40286	11.46	0.80	30.85						
qBNP-Chr23-4	E1	△ *t*1-2	NAU3588	NAU5373a	3.93	-0.71	7.55						
	E2	△ *t*1-2	NAU3588	NAU5373a	5.22	-1.12	10.90						
qBNP-Chr25-1	E2	△ *t*2-3	SWU19676	NAU2968				4.14	-0.73	11.54			
qBNP-Chr25-2	E2	△ *t*1-2	NAU2968	DPL0377				5.12	0.83	16.06			
	E2	△ *t*3-4	DPL0377	SWU19413				3.21	-0.39	8.02			
qBNP-Chr25-3	E1	△ *t*1-2	DPL0377	SWU19413				3.77	0.46	8.70			
	E2	△ *t*1-2	SWU19413	SWU19431				6.68	0.79	14.25			
	E1	△ *t*3-4	SWU19413	SWU19431	5.15	-0.51	11.12						
qBNP-Chr25-4	E2	△ *t*1-2	SWU19412	NAU3112				5.04	0.86	16.97			
qBNP-Chr25-5	E2	△ *t*2-3	DPL0270	SWU16437	3.94	0.74	13.89						
qBNP-Chr26-1	E3	△ *t*2-3	SWU16777	SWU16780	3.41	1.21	7.24						
	E3	△ *t*2-3	SWU16777	SWU16780							5.00	-0.93	10.72

Bold figures indicate the conditional QTL was detected in more than one environments, stages or populations simultaneously

Env., Environment, E1: Handan; E2: Cangzhou; E3: Xiangyang

Effect, the genetic expectation of a QTL effect obtained is the additive effect (A) when estimated from the RILs and RIL′s, the additive and dominance effects (A+D) from the BCF_1 mean values, and the dominance effect (D) from the MPH values

Var%, phenotypic variation explained by a single QTL

Table S4 Summary of QTL and conditional QTL detected at different stages

	XZ hybrid			XZV hybrid		
	RIL	BC	MPH	RIL	BC	MPH
*t*1	12	5	5	4	5	3
*t*2	9	10	3	3	6	5
*t*3	9	5	2	4	4	5
*t*4	5	6	3	3	5	1
△ *t*1-2	8	9	5	2	5	3
△ *t*2-3	5	3	1	2	2	3
△ *t*3-4	3	5	3	4	1	0

Table S5 Main effects and environmental interactions detected for boll number per plant in RIL and RILV populations by inclusive composite interval mapping

Stage	Chi.	Position	Flanking markers		LOD	V(A)	V(AE)	A	AE1	AE2	AE3
RIL population											
*t*1	1	24	SWU10930	ICR03295	3.82	2.16	0.09	-0.14	0.01	-0.04	0.02
	1	35	NAU3177	ICR03724	4.24	2.51	0.33	-0.15	-0.07	0.00	0.07
	2	41	SWU11889	SWU11887	15.60	10.19	0.20	0.30	0.05	-0.01	-0.05
	6	46	CGR5108	ICR03206	2.62	1.48	0.25	0.12	-0.01	0.06	-0.05
	9	197	SWU15157	SWU14934	2.50	1.55	0.06	-0.12	-0.01	-0.02	0.03
	11	201	NAU1014	ICR10344	3.84	1.60	0.20	-0.12	0.06	-0.01	-0.04
	12	59	Gh631	HAU1321	3.48	1.63	0.99	-0.12	-0.01	-0.11	0.12
	14	51	BNL3661	PGML2498	2.57	1.50	0.42	0.12	0.06	0.02	-0.08
	16	163	SWU10038	ICR00016	3.40	0.98	1.54	-0.09	0.03	-0.16	0.12
	18	112	NAU748	SWU22192	2.59	1.46	0.18	-0.11	-0.01	-0.05	0.05
	21	157	SWU15915	SWU0189	12.39	8.04	1.01	-0.27	-0.06	-0.08	0.14
	24	34	SWU13267	BNL1521	2.95	1.85	0.18	0.13	0.04	0.02	-0.05
*t*2	1	29	CGR6129	DPL0790	2.78	1.81	0.29	-0.31	-0.17	0.05	0.12
	1	48	SWU10986	NAU2218	5.73	4.12	1.42	-0.46	-0.09	-0.28	0.37
	1	165	SWU0077	HAU1417	3.19	1.58	0.04	-0.29	0.05	-0.06	0.01
	2	40	SWU11889	SWU11887	13.90	4.94	0.69	0.51	0.01	-0.24	0.23
	5	35	NAU6240	PGML1671	2.74	0.62	0.22	0.18	0.00	-0.13	0.13
	11	197	NAU1014	ICR10344	2.58	1.66	0.84	-0.29	-0.17	-0.12	0.29
	12	60	HAU1316	NAU3519	2.76	1.59	1.02	-0.29	-0.13	-0.19	0.32
	18	118	SWU22192	DPL0864	3.39	2.47	0.56	-0.36	-0.10	-0.14	0.24
	21	156	SWU14431a	SWU15915	5.01	2.11	0.58	-0.34	-0.20	0.23	-0.03
	21	185	CGR5808	HAU0423	6.19	4.13	1.67	-0.47	-0.15	-0.27	0.41
	22	1	SWU21635	SWU21646	3.72	2.42	0.40	-0.35	0.01	-0.18	0.17
	24	35	SWU13267	BNL1521	3.12	1.68	0.08	0.29	-0.05	0.09	-0.04
	25	19	CGR6864	SWU19815	2.51	1.36	0.66	-0.27	0.12	-0.26	0.14
	26	28	NAU2175	SWU17336	2.62	1.86	0.29	0.31	0.14	0.01	-0.15
	27	4	SWU10994	HAU1001	4.70	3.04	1.11	-0.40	-0.18	-0.15	0.34
*t*3	1	25	CGR6129	DPL0790	2.80	2.06	0.02	-0.43	-0.06	0.03	0.03
	1	49	SWU10986	NAU2218	5.32	3.67	0.41	-0.58	-0.04	-0.21	0.25
	2	42	SWU11887	SWU11976	7.18	3.88	1.16	0.59	0.05	-0.42	0.37
	7	88	Gh474	SWU10785	2.52	1.56	0.68	0.38	0.35	-0.18	-0.17
	11	199	NAU1014	ICR10344	6.15	3.45	0.59	-0.56	0.13	0.20	-0.33
	12	77	HAU1316	NAU3519	2.54	1.25	1.06	-0.34	-0.43	0.16	0.28
	14	53	CGR6784	NAU874	3.83	2.76	0.03	0.50	0.01	0.06	-0.07
	14	77	SWU14543	ICR12037	3.06	1.97	0.04	0.42	-0.08	0.02	0.06

Stage	Chi.	Position	Flanking markers		LOD	V(A)	V(AE)	A	AE1	AE2	AE3
	19	120	SWU14431b	SWU17782	3.43	2.50	0.20	-0.48	-0.19	0.11	0.08
	20	138	DPL0319	HAU1378	3.28	2.43	0.22	-0.47	-0.20	0.07	0.13
	21	157	SWU15915	SWU0189	3.92	2.60	0.18	-0.49	-0.08	0.18	-0.10
	21	184	CGR5808	HAU0423	3.85	2.72	0.35	-0.50	-0.25	0.11	0.15
	22	1	SWU21635	SWU21646	3.59	2.14	0.40	-0.44	0.16	-0.27	0.10
	23	8	Gh327	SWU14770	4.34	2.85	0.58	-0.51	-0.21	-0.11	0.32
	25	46	BNL3594	DPL0282	2.71	1.85	0.12	-0.41	-0.08	-0.07	0.15
	26	2	HAU1571	BNL598	3.63	2.36	0.68	0.46	0.29	0.03	-0.32
	27	0	SWU10994	HAU1001	6.01	4.19	0.56	-0.62	-0.10	-0.21	0.31
*t*4	1	48	SWU10986	NAU2218	6.56	4.67	0.15	-0.67	0.16	-0.12	-0.04
	2	41	SWU11889	SWU11887	3.56	1.19	1.72	0.34	0.08	-0.53	0.45
	3	119	SWU12765	NAU3839	2.51	1.73	0.17	0.41	0.17	-0.13	-0.04
	5	115	TMB1296	HAU1603	2.56	1.22	1.08	-0.34	0.03	0.38	-0.41
	5	136	SWU17787	SWU13378	2.87	2.04	0.27	-0.44	-0.13	0.23	-0.10
	11	199	NAU1014	ICR10344	3.19	2.03	0.58	-0.44	0.19	0.14	-0.33
	12	59	Gh631	HAU1321	3.29	1.52	0.57	-0.38	0.13	-0.33	0.19
	19	116	PGML4342	SWU14431b	2.93	2.02	0.00	-0.44	-0.02	-0.01	0.03
	20	138	DPL0319	HAU1378	2.90	1.80	0.32	-0.41	-0.25	0.12	0.13
	22	2	SWU21646	SWU21585	4.71	3.49	0.11	-0.58	0.10	0.04	-0.14
	24	86	SWU13745	Gh273	3.98	1.80	0.86	0.41	-0.35	0.35	-0.01
	25	55	BNL3594	DPL0282	2.60	1.84	0.01	-0.42	0.04	-0.03	-0.02
	27	0	SWU10994	HAU1001	5.15	3.53	0.51	-0.58	-0.31	0.16	0.15
RILV population											
*t*1	15	5	NAU3736	SWU11691	2.88	1.83	0.83	0.13	0.04	0.08	-0.13
	16	39	NAU747	HAU1129	2.65	1.18	0.02	0.11	0.01	0.01	-0.02
	16	99	SWU10266	SWU18366	5.10	3.71	1.25	-0.19	0.00	-0.14	0.14
	21	57	CGR5148	SHIN0337	2.95	2.07	0.55	0.14	0.04	0.06	-0.10
	23	278	NAU2238	NAU3588	8.37	6.39	2.67	-0.25	-0.02	-0.19	0.21
	25	28	SWU19676	NAU2968	2.56	1.01	0.03	0.10	-0.02	0.02	-0.01
	26	52	DC30107	DPL0070	3.52	2.23	0.31	0.15	0.01	0.06	-0.07
	30	2	TMB1638	CGR6812	2.68	1.80	0.87	0.13	0.00	0.11	-0.11
*t*2	16	39	NAU747	HAU1129	2.79	1.72	0.20	0.43	-0.07	0.20	-0.13
	16	100	SWU18366	SWU18579	4.64	3.26	0.94	-0.59	0.06	-0.41	0.36
	23	278	NAU2238	NAU3588	12.54	8.69	2.90	-0.96	-0.22	-0.54	0.76
	25	94	NAU3112	NAU4964	2.68	1.61	0.09	0.41	-0.01	0.13	-0.12
	26	52	DC30107	DPL0070	6.56	4.01	0.23	0.65	0.07	0.14	-0.22
*t*3	15	0	NAU3736	SWU11691	4.33	2.86	0.12	0.78	-0.20	0.19	0.01
	16	100	SWU18366	SWU18579	4.05	2.89	0.20	-0.79	0.29	-0.17	-0.12

Stage	Chi.	Position	Flanking markers		LOD	V(A)	V(AE)	A	AE1	AE2	AE3
	23	278	NAU2238	NAU3588	9.82	4.05	1.25	-0.93	-0.27	-0.45	0.72
	26	52	DC30107	DPL0070	4.87	3.51	0.47	0.86	-0.28	-0.17	0.44
	31	67	HAU0355	SWU16777	3.30	2.08	1.68	0.66	-0.68	-0.09	0.77
	31	87	SWU16735	SWU16755	2.75	1.98	0.14	-0.65	0.20	0.02	-0.22
*t*4	15	0	NAU3736	SWU11691	4.39	2.52	0.10	0.76	-0.19	0.18	0.01
	16	100	SWU18366	SWU18579	3.22	2.31	0.41	-0.73	0.43	-0.25	-0.18
	23	91	BNL3482	HAU0244	2.95	1.23	0.23	-0.55	0.02	-0.30	0.28
	23	278	NAU2238	NAU3588	6.40	2.07	0.93	-0.69	-0.12	-0.49	0.61
	26	52	DC30107	DPL0070	3.96	2.97	0.48	0.82	-0.42	0.03	0.39
	31	67	HAU0355	SWU16777	3.63	2.15	2.10	0.70	-0.85	0.02	0.84
	31	87	SWU16735	SWU16755	2.53	1.94	0.45	-0.66	0.39	0.00	-0.39

Chi represents the linkage group number of the loci detected

Position: The scanning position in cM on the linkage group.

V(A): Phenotypic variation explained by additive and dominance effect at the current scanning position.

V(AE): Phenotypic variation explained by additive and dominance by environment effect at the current scanning position.

AE effect, the additive and dominance effect.

AE effect, the additive and dominance × environment effect.

AE1 to AE3 represent AE effects detected in Handan, Cangzhou and Xiangyang, respectively.

Table S6 Main effects and environmental interactions detected for boll number per plant in BCF_1 and $BCVF_1$ populations by inclusive composite interval mapping

Stage	Chi	Position	Flanking markers		LOD	V(A)	V(AE)	A	AE1	AE2	AE3
BCF_1 population											
*t*1	1	33	NAU4045	SWU10959	3.45	2.49	0.12	-0.12	-0.04	0.02	0.01
	2	41	SWU11889	SWU11887	3.55	1.55	0.34	0.10	-0.06	0.00	0.05
	15	2	DC40175	SWU11630	2.61	0.00	1.30	0.00	-0.07	-0.05	0.12
	20	118	SWU20035	DPL0319	3.14	2.16	0.66	-0.11	-0.08	0.00	0.08
	21	157	SWU15915	SWU0189	2.66	1.78	0.07	-0.10	0.01	-0.03	0.02
	21	192	HAU0423	CGR5806	2.77	1.96	0.18	-0.11	-0.03	-0.01	0.05
	24	33	Gh268	SWU13268	5.21	3.67	0.00	0.15	0.00	0.00	-0.01
	25	17	SWU19848	CGR6864	2.81	2.06	0.38	-0.11	-0.06	0.00	0.06
*t*2	1	30	CGR6129	DPL0790	4.50	0.90	0.60	-0.17	0.09	0.10	-0.19
	1	160	CGR5663	NAU2343	4.76	1.79	2.41	-0.24	0.01	-0.34	0.33
	9	59	SWU15194	HAU190	3.61	1.78	0.60	0.24	-0.15	0.18	-0.04
	11	153	PGML2202	CGR6580	3.60	0.37	1.95	0.11	-0.02	0.31	-0.29
	14	75	SWU14545	SWU14543	3.29	0.90	0.32	0.17	-0.03	-0.11	0.13
	16	26	SWU10627	PGML1309	3.09	0.72	0.53	-0.15	-0.03	0.17	-0.14
	20	116	SWU20035	DPL0319	2.52	1.54	0.54	-0.22	0.05	-0.18	0.12
	21	52	SWU16487	SWU16488	3.10	0.85	1.09	-0.16	-0.20	0.25	-0.05
	21	212	HAU0423	CGR5806	4.47	2.77	1.42	-0.31	0.06	-0.29	0.23
	24	32	PGML4657	Gh454	7.91	3.41	0.31	0.33	-0.13	0.01	0.11
	26	149	SWU0514	SWU18488	3.04	2.13	0.57	-0.26	-0.01	-0.16	0.17
	27	12	SWU11038	SWU11384	3.96	2.39	0.17	-0.27	0.02	-0.10	0.07
*t*3	1	35	NAU3177	ICR03724	3.04	1.88	0.68	-0.32	0.27	-0.12	-0.15
	1	48	SWU10986	NAU2218	2.75	1.93	0.52	-0.32	0.16	0.07	-0.23
	1	159	CGR5663	NAU2343	4.69	1.67	1.92	-0.29	0.10	-0.43	0.33
	4	1	SWU18881	NAU2701	2.75	1.66	0.31	-0.29	-0.05	-0.13	0.17
	14	53	CGR6784	NAU874	4.30	3.41	0.11	0.42	-0.07	-0.04	0.10
	14	72	Gh120	PGML1884	3.90	2.56	0.46	0.36	0.10	-0.22	0.12
	16	158	SWU10056	SWU10037	2.52	0.01	2.06	-0.02	-0.22	-0.25	0.47
	21	254	DPL0777	CGR5217	3.74	0.01	2.74	-0.03	0.52	-0.15	-0.36
*t*4	1	159	CGR5663	NAU2343	3.25	0.77	1.67	-0.21	0.06	-0.41	0.34
	9	63	HAU1618	NAU2873	3.22	2.21	0.13	0.36	0.03	0.08	-0.12
	11	72	CGR5421	ICR08245	4.35	3.16	0.51	-0.43	0.24	-0.06	-0.17
	12	59	Gh631	HAU1321	3.00	2.26	0.17	-0.36	0.00	0.12	-0.12
	14	54	NAU874	SWU13824	2.96	2.05	0.14	0.34	-0.01	0.11	-0.10
	24	30	PGML4657	Gh454	3.59	1.86	1.50	0.33	-0.08	-0.31	0.40
	26	156	SWU18488	SWU18672	3.24	2.02	0.17	-0.34	-0.10	-0.04	0.14

Stage	Chi	Position	Flanking markers		LOD	V(A)	V(AE)	A	AE1	AE2	AE3
	27	58	CGR6356	SWU11632	3.60	2.57	0.18	-0.39	-0.08	0.14	-0.07
			BCVF$_1$ population								
*t*1	4	55	SWU16783	SWU18876	2.91	2.11	0.40	0.11	0.04	0.03	-0.06
	12	128	Gh631	COT107	3.33	1.81	1.33	-0.10	0.00	-0.11	0.10
	21	58	SHIN0337	SWU16370	3.94	2.85	0.56	0.12	0.06	0.02	-0.07
	23	62	SHIN1076	BNL3482	2.64	0.15	0.61	-0.03	-0.01	0.08	-0.06
	23	148	ICR06429	SWU0506	2.59	1.76	0.59	0.11	0.08	0.01	-0.08
	23	277	NAU2238	NAU3588	6.55	4.18	1.86	-0.15	0.01	-0.13	0.12
	24	3	PGML1207	Gh54	4.05	0.93	0.45	0.07	0.02	-0.07	0.05
*t*2	2	9	DPL0217	CGR6695	3.06	1.61	0.11	-0.24	0.08	-0.08	0.00
	16	100	SWU18366	SWU18579	4.36	2.47	0.45	-0.30	-0.17	0.03	0.14
	19	47	DC40130	SWU17897	2.54	0.36	1.28	0.12	0.09	0.21	-0.30
	21	58	SHIN0337	SWU16370	3.29	2.03	0.14	0.27	-0.04	0.10	-0.06
	23	58	SHIN1076	BNL3482	3.68	0.05	1.18	-0.04	0.18	0.12	-0.30
	23	279	NAU3588	NAU5373a	3.92	1.62	1.08	-0.24	-0.26	0.04	0.22
	24	61	Gh54	Gh454	3.12	0.88	0.39	0.19	-0.16	0.03	0.13
	25	62	DPL0377	SWU19413	8.10	4.00	5.06	0.38	-0.29	0.61	-0.32
	34	5	JESPR297	ICR00647	2.92	1.72	0.35	-0.25	0.10	-0.16	0.06
*t*3	2	114	SWU11976	SWU12001	2.74	1.27	1.23	-0.25	0.17	-0.34	0.18
	23	279	NAU3588	NAU5373a	5.08	2.00	1.53	-0.31	-0.28	-0.09	0.37
*t*4	2	115	SWU11976	SWU12001	2.60	0.80	1.32	-0.21	0.09	-0.36	0.27
	23	0	CGR5158	HAU1758	2.98	0.00	1.92	0.01	0.44	-0.37	-0.07
	23	278	NAU2238	NAU3588	4.37	0.62	2.29	-0.18	-0.34	-0.14	0.48

See footnotes of additional table S5 for explanations

Table S7 Main effects and environmental interactions detected for boll number per plant in two MPH data by inclusive composite interval mapping

Stage	Chi	Position	Flanking markers		LOD	V(A)	V(AE)	A	AE1	AE2	AE3
MPH (XZ hybrid)											
*t*1	2	57	SWU11887	SWU11976	3.95	3.44	0.51	-0.11	-0.12	0.05	0.07
	8	31	Gh197	DC20094	3.26	2.97	0.29	0.10	0.10	-0.03	-0.07
	11	28	NAU5428	Gh256	2.79	2.15	0.65	-0.08	-0.02	0.04	-0.03
	16	30	SWU20341	DPL0897	3.52	0.04	3.48	0.01	-0.10	0.15	-0.05
	24	2	Gh298	SWU13133	3.73	0.98	2.75	0.06	-0.09	0.00	0.09
*t*2	2	92	PGML0700	SWU12016	3.04	2.63	0.41	-0.23	-0.21	-0.01	0.21
	11	148	CAU0003	DC40250	3.43	1.95	1.48	0.20	0.16	0.13	-0.29
	15	16	DPL0182	SWU11691	2.62	0.00	2.62	-0.01	0.02	-0.23	0.21
	26	202	SWU18697	PGML1289	3.22	0.15	3.07	-0.06	0.31	-0.13	-0.18
	28	85	SWU12343	SWU14060	2.87	1.33	1.54	0.16	-0.12	0.27	-0.15
*t*3	1	165	SWU0077	HAU1417	3.13	0.06	3.06	-0.05	-0.07	-0.34	0.41
	19	35	NAU3437	NAU2894	2.73	0.12	2.62	-0.06	0.41	-0.15	-0.26
	26	26	SWU17395	DC30107	2.57	0.74	1.83	-0.16	0.00	0.26	-0.25
*t*4	9	63	HAU1618	NAU2873	3.47	1.66	1.81	0.27	0.28	0.04	-0.31
	11	73	CGR5421	ICR08245	3.51	3.10	0.41	-0.36	0.23	-0.08	-0.15
	21	52	SWU16487	SWU16488	2.73	1.63	1.10	-0.27	-0.14	-0.10	0.25
	27	57	CGR6356	SWU11632	2.52	2.08	0.43	-0.30	-0.09	0.18	-0.09
MPH (XZV hybrid)											
*t*1	2	9	DPL0217	CGR6695	2.97	2.07	0.15	-0.11	-0.03	0.00	0.04
	12	110	CGR6847	SWU17197	2.70	1.57	0.80	-0.09	-0.05	-0.05	0.09
	16	15	NAU747	HAU1129	2.99	0.70	0.18	0.06	-0.03	-0.01	0.04
	19	0	NAU2816	PGML4342	2.54	0.40	1.54	0.05	0.00	0.11	-0.11
	23	50	HAU1758	SHIN1076	2.89	0.01	1.04	0.01	0.05	0.06	-0.11
	24	2	PGML1207	Gh54	2.56	0.82	0.09	0.07	0.00	-0.03	0.03
	39	96	SWU16437	SWU16432	2.69	1.80	0.15	0.10	0.02	0.02	-0.04
*t*2	1	193	DPL0790	ICR03725	3.01	0.02	2.47	-0.03	-0.38	0.36	0.02
	2	9	DPL0217	CGR6695	7.40	4.48	0.28	-0.40	0.13	-0.11	-0.03
	23	57	SHIN1076	BNL3482	3.64	0.03	1.79	0.03	0.21	0.15	-0.36
	25	46	NAU2968	DPL0377	2.62	0.29	2.46	0.10	-0.18	0.42	-0.23
	26	53	DC30107	DPL0070	2.55	1.91	0.14	-0.26	-0.05	-0.05	0.10
	37	36	JESPR154	BNL5602	2.68	2.02	0.82	-0.27	0.04	-0.23	0.18
*t*3	2	6	DPL0217	CGR6695	4.71	3.44	1.35	-0.51	0.34	0.09	-0.43
	23	75	BNL3482	HAU0244	3.26	0.93	0.50	0.28	0.28	-0.20	-0.08
	26	53	DC30107	DPL0070	4.56	3.34	0.21	-0.50	0.17	-0.06	-0.11
	31	67	HAU0355	SWU16777	3.10	2.15	0.90	-0.40	0.17	0.20	-0.37

Stage	Chi	Position	Flanking markers		LOD	V(A)	V(AE)	A	AE1	AE2	AE3
*t*4	2	16	SWU11013	DPL0041	2.80	2.07	0.94	-0.42	0.33	0.04	-0.36
	3	14	SWU12840	NAU2742	3.18	2.03	0.07	-0.42	0.10	-0.09	0.00
	21	231	BNL3171	HAU2937	4.05	3.06	0.70	0.51	-0.34	0.09	0.25
	26	53	DC30107	DPL0070	4.14	3.22	0.38	-0.52	0.20	0.04	-0.24
	31	67	HAU0355	SWU16777	3.11	2.45	1.22	-0.46	0.26	0.19	-0.45
	31	117	SWU16730	SWU16721	3.24	2.24	0.18	0.44	-0.17	0.05	0.12

See footnotes of additional table S5 for explanations

Table S8 Epistatic effects and environmental interactions detected for boll number per plant in RIL and RILV populations using two-locus analysis by inclusive composite interval mapping

Stage	Chi	Flanking markers		Chj	Flanking markers		LOD	V(AA)	V(AAE)	AA	AAE1	AAE2	AAE3
RIL population													
*t*1	1	ICR03724	ICR03725	2	SWU11887	SWU11976	11.0	4.63	0.90	-0.20	-0.05	-0.08	0.13
	3	SWU12732	SWU12783	5	PGML1917	SWU17715	5.52	2.48	0.53	-0.15	-0.02	-0.08	0.09
	4	SWU16783	NAU3868	9	HAU190	HAU1618	5.71	1.86	1.10	-0.13	-0.04	-0.10	0.14
	6	ICR10602	SWU19656	9	SWU15511	SWU15413	5.97	2.69	0.51	0.16	0.05	0.05	-0.10
	5	PGML1917	SWU17715	9	CGR6876	CGR5758	5.73	1.91	0.98	0.13	0.04	0.09	-0.13
	3	SWU12819	SWU12765	9	NAU3966	SWU15157	5.57	0.91	1.55	-0.09	-0.06	-0.10	0.17
	6	ICR03206	NAU896	14	**BNL3661**	**PGML2498**	5.09	1.20	1.15	-0.10	-0.06	-0.09	0.14
	1	NAU3384	CGR5663	14	**BNL3661**	**PGML2498**	5.93	2.77	0.51	0.16	0.08	0.01	-0.09
	7	CGR5372	SWU10205	14	NAU874	SWU13824	5.87	2.36	0.82	-0.15	-0.07	-0.06	0.12
	11	CGR6580	SWU15972	14	PGML1368	PGML1568	5.08	2.11	0.43	-0.15	-0.02	-0.07	0.08
	3	SWU12732	SWU12783	16	PGML1709	SWU10627	5.27	2.14	0.84	-0.14	-0.01	-0.10	0.11
	10	CGR5873	ICR00093	16	Gh56	NAU5120	5.31	2.26	0.55	0.14	0.06	0.04	-0.10
	16	SWU10056	SWU10037	17	SWU12818	CGR5576	5.11	1.66	0.44	0.13	0.05	0.05	-0.09
	12	DPL0303	COT107	17	CGR5576	NAU3765	6.25	3.08	0.49	-0.17	-0.01	-0.07	0.09
	2	SWU12126	SWU12147	18	DC40150	ICR02849	5.29	2.49	0.47	-0.15	-0.04	-0.05	0.09
	9	SWU15194	HAU190	19	NAU5330	Gh72	6.35	2.35	0.87	-0.15	-0.10	-0.02	0.12
	2	SWU11887	SWU11976	19	SWU17897	CGR5539	5.09	1.99	0.60	0.13	0.03	0.07	-0.10
	16	SWU10062	SWU10094	20	SWU20246	SWU20501a	7.48	3.71	0.28	0.18	0.04	0.03	-0.07
	7	NAU1357	SWU10067	20	SWU20035	DPL0319	5.30	2.83	0.08	0.16	0.04	0.00	-0.03
	15	CGR6889	DPL0182	21	SWU16649	BNL1552	5.44	1.73	0.75	0.12	0.09	0.02	-0.11
	2	SWU12393	SWU11013	21	SWU16138	BNL1053	5.38	1.49	1.24	-0.12	-0.08	-0.07	0.15
	21	SWU0830	HAU2004	21	SWU14431a	SWU15915	5.20	1.99	0.51	0.15	0.07	0.04	-0.11
	5	DPL0022	SWU17787	21	SWU0189	DPL0050a	5.14	1.76	0.75	-0.13	-0.06	-0.06	0.12
	16	SWU10214	Gh56	21	CGR5808	HAU0423	7.13	2.98	0.76	0.16	0.04	0.08	-0.12
	11	CGR5421	ICR08245	21	CGR5806	DPL0777	5.09	2.47	0.31	0.15	0.03	0.04	-0.08
	14	SWU14224	DPL0565	22	CAU0161	NAU2026	5.25	1.36	1.33	0.12	0.05	0.11	-0.16
	13	NAU2893	Gh157	23	PGML4186	NAU3100	5.30	2.36	0.61	-0.15	-0.07	-0.03	0.11
	14	ICR12037	CGR5675	23	PGML4186	NAU3100	5.60	1.44	1.31	0.12	0.04	0.11	-0.15
	2	SWU12393	SWU11013	24	BNL1521	HAU2504	7.26	2.26	1.07	-0.14	-0.09	-0.05	0.14
	11	CAU0003	DC40250	25	CGR6864	SWU19815	6.43	2.69	0.67	0.16	0.08	0.02	-0.11
	14	NAU874	SWU13824	25	DPL0282	SWU19763	6.97	2.65	0.74	0.16	0.09	0.02	-0.11
	13	SHIN1462	SWU22374	25	DPL0282	SWU19763	5.93	1.96	0.79	0.13	-0.03	0.11	-0.09
	4	SWU12672	HAU1332	26	SWU17467	SWU17419	5.68	2.79	0.31	0.16	0.03	0.05	-0.08
	2	SWU11887	SWU11976	26	SWU17432	SWU17395	7.19	3.29	0.57	-0.17	-0.06	-0.04	0.10
	1	BNL2827a	NAU6367	26	SWU18681	SWU0598	5.18	2.24	0.50	-0.14	-0.04	-0.06	0.10
	20	SWU20649	SWU20636	27	SWU10994	HAU1001	5.13	1.89	0.74	0.13	0.03	0.08	-0.11
	19	NAU3437	NAU2894	27	SWU11384	ICR11885	5.84	3.15	0.22	0.17	0.05	0.02	-0.06
	20	CER0167	SWU20064	28	CGR5534	SHIN0219	8.67	2.88	1.29	0.16	0.08	0.08	-0.15
	21	CGR5806	DPL0777	28	NBRI0014	SWU12107	5.17	1.97	0.74	0.13	0.08	0.03	-0.11
	7	SWU10205	HAU1483a	29	DC20127	DPL0252	5.39	2.53	0.44	-0.15	-0.03	-0.05	0.09
	6	**CGR5108**	**ICR03206**	29	BNL3261	CGR5111	5.68	2.23	0.61	0.14	0.03	0.08	-0.10
	9	NAU1282	CGR6771	30	BNL243	CER0168	5.06	1.89	0.80	0.13	0.10	0.00	-0.10
*t*2	1	**SWU10986**	**NAU2218**	1	CGR5663	NAU2343	5.01	2.45	0.36	-0.35	-0.13	-0.06	0.18
	1	**SWU10986**	**NAU2218**	2	SWU11887	SWU11976	6.31	3.12	0.21	-0.40	-0.08	-0.07	0.15

Stage	Chi	Flanking markers		Chj	Flanking markers		LOD	V(AA)	V(AAE)	AA	AAE1	AAE2	AAE3
	1	HAU1417	NAU2437	5	PGML1917	SWU17715	5.06	1.15	1.14	0.25	0.24	0.10	-0.34
	1	HAU1417	NAU2437	6	ICR00143	CGR5108	5.17	1.99	0.86	-0.33	-0.21	-0.08	0.29
	6	ICR03206	NAU896	6	NAU896	BNL3650	6.36	2.75	0.78	0.50	0.33	0.01	-0.34
	6	ICR03206	NAU896	7	Gh474	SWU10785	6.09	2.22	0.84	-0.35	-0.19	-0.11	0.30
	3	SWU12819	SWU12765	8	CGR6508	Gh197	5.33	2.02	0.86	0.33	-0.05	0.28	-0.23
	2	SWU12393	SWU11013	8	HAU1470a	SHIN1341	5.39	2.33	0.83	0.35	0.08	0.21	-0.29
	5	PGML1917	SWU17715	9	NAU1282	CGR6771	5.16	1.52	1.51	-0.28	-0.39	0.10	0.29
	3	SWU12840	NAU2742	11	PGML2202	CGR6580	6.10	2.18	1.11	0.34	0.27	0.05	-0.32
	3	HAU2424	CER0028	13	SHIN1462	SWU22374	6.24	2.20	1.25	0.34	0.27	0.08	-0.35
	13	SWU22374	HAU2857	13	DPL0894	SWU10800	5.63	2.57	0.83	0.40	0.29	-0.04	-0.25
	7	NAU1357	SWU10067	14	SWU13909	TMB0071	9.25	4.48	0.92	0.49	0.28	-0.02	-0.26
	1	NAU6367	MUSS422	14	SWU14224	DPL0565	5.84	2.21	0.89	0.34	0.17	0.13	-0.31
	14	SWU13909	TMB0071	14	ICR12130	DPL0502	5.64	2.84	0.44	0.38	0.05	0.15	-0.20
	7	CGR5001	CGR6586	15	DC40183	DC40175	8.04	4.30	0.51	0.47	0.15	0.07	-0.23
	1	NAU3384	CGR5663	16	SWU10266	DC40065	9.14	4.05	1.14	-0.47	-0.09	-0.25	0.33
	14	NAU3820	NAU2960	16	NAU862	CGR6802	5.49	2.94	0.39	0.39	0.16	0.02	-0.19
	9	Gh158	DC40407	16	CGR6802	HAU1129	7.11	3.70	0.52	0.44	0.20	0.01	-0.21
	12	DPL0303	COT107	16	SWU10062	SWU10094	7.95	3.58	0.97	0.44	0.23	0.08	-0.31
	11	CGR6580	SWU15972	16	SWU10038	ICR00016	7.53	3.31	0.85	0.42	0.24	0.04	-0.28
	11	NAU3390	NAU2460	17	NAU3765	SWU14627	6.33	2.80	0.97	-0.39	-0.24	-0.08	0.32
	5	PGML4350	SWU17781	18	DC40150	ICR02849	5.59	2.33	1.23	0.37	0.36	-0.08	-0.28
	1	SWU11191	BNL2827b	19	NAU5330	Gh72	5.31	2.34	0.77	0.35	0.08	0.19	-0.28
	10	SWU20501b	CGR5873	19	NAU3437	NAU2894	5.30	2.37	0.52	0.36	0.13	0.12	-0.25
	6	ICR03206	NAU896	19	SWU14431b	SWU17782	6.49	2.18	1.05	0.34	0.10	0.22	-0.32
	10	SWU20501b	CGR5873	20	SWU20700	CGR5548	6.52	3.62	0.47	-0.45	-0.18	-0.04	0.22
	19	PGML4342	SWU14431	20	SWU20636	CGR6154	6.12	2.75	0.70	-0.38	-0.18	-0.08	0.26
	16	SWU10062	SWU10094	20	SWU20246	SWU20501a	6.85	3.79	0.24	0.45	0.00	0.13	-0.14
	2	PGML0700	SWU12016	21	Gh451	SWU16489	8.19	3.74	0.64	-0.44	-0.14	-0.11	0.26
	3	Gh663	CGR6528	21	SWU16488	SWU16138	5.26	2.18	0.70	-0.34	-0.20	-0.07	0.26
	21	SWU0830	HAU2004	21	**SWU14431**	**SWU15915**	5.97	1.89	0.60	0.39	0.28	-0.01	-0.27
	10	CGR5873	ICR00093	21	BNL3171	CGR5808	5.05	1.55	1.39	-0.29	-0.37	0.09	0.27
	16	Gh56	NAU5120	21	BNL3171	CGR5808	5.47	2.04	1.17	0.33	0.31	-0.02	-0.28
	17	CGR5576	NAU3765	21	**CGR5808**	**HAU0423**	5.63	2.33	1.27	0.35	0.31	0.01	-0.31
	1	**SWU10986**	**NAU2218**	21	HAU0423	CGR5806	5.81	3.31	0.35	-0.43	-0.03	-0.15	0.18
	13	SWU22309	SWU22324	21	HAU0423	CGR5806	5.78	2.28	0.90	0.36	0.18	0.14	-0.32
	20	SWU20246	SWU20501	22	SWU21646	SWU21585	5.21	2.24	0.64	0.34	0.19	0.06	-0.24
	1	SWU10912	DPL0090	22	SWU21586	PGML1712	5.01	1.81	0.91	0.32	0.25	0.04	-0.29
	8	CGR6508	Gh197	23	SWU14807	PGML4185	5.07	2.78	0.52	0.38	0.21	-0.02	-0.19
	11	CER0098	CGR5421	24	CGR5202	Gh298	5.26	2.13	0.77	0.34	0.10	0.19	-0.29
	9	PGML2830	CGR6876	24	Gh298	SWU13133	5.07	1.73	1.01	0.30	0.18	0.14	-0.33
	11	NAU3390	NAU2460	25	HAU1382	SWU19848	7.59	3.88	0.76	-0.45	-0.20	-0.07	0.27
	16	SWU10056	SWU10037	25	HAU1382	SWU19848	5.50	1.76	1.03	-0.31	-0.06	-0.25	0.31
	10	BNL2960	SWU20511	25	DPL0282	SWU19763	5.55	2.26	0.61	-0.34	-0.19	-0.05	0.24
	9	Gh27	SWU15194	25	SWU19430	PGML1219	5.11	1.80	0.69	-0.30	0.02	-0.24	0.22
	13	DPL0572	HAU2558	25	SWU19430	PGML1219	5.37	2.17	0.83	-0.34	-0.22	-0.06	0.27
	24	BNL1521	HAU2504	25	SWU19430	PGML1219	5.04	1.73	0.80	0.30	0.02	0.24	-0.26
	25	SWU19848	CGR6864	26	BNL598	PGML1637	5.45	1.72	1.12	0.30	0.16	0.17	-0.33
	20	SWU20246	SWU20501	26	BNL598	PGML1637	5.22	1.99	0.82	-0.32	-0.14	-0.15	0.30
	7	SWU10064	NAU3181	26	SWU17467	SWU17419	5.33	2.93	0.26	0.39	0.05	0.11	-0.16

Stage	Chi	Flanking markers		Chj	Flanking markers		LOD	V(AA)	V(AAE)	AA	AAE1	AAE2	AAE3
	13	SWU22413	CGR5331	26	SWU17251	C2_0135	5.12	3.01	0.20	-0.40	-0.06	-0.09	0.14
	14	CIR228	BNL2485	26	C2 0135	PGML2321	5.44	2.40	0.58	0.35	0.14	0.10	-0.24
	2	PGML0700	SWU12016	26	C2 0135	PGML2321	7.50	2.93	0.71	-0.39	-0.17	-0.10	0.27
	21	SWU15915	SWU0189	27	**SWU10994**	**HAU1001**	5.69	2.52	0.72	-0.36	-0.14	-0.13	0.27
	5	HAU1603	PGML4457	27	CGR6857	ICR11205	8.91	4.39	0.64	0.49	0.04	0.21	-0.24
	13	DPL0894	SWU10800	28	HAU3071	CGR5534	5.16	1.62	0.98	-0.29	-0.20	-0.11	0.31
	21	SWU0189	DPL0050a	29	DC20127	DPL0252	6.75	2.61	1.35	-0.37	-0.33	0.04	0.28
	17	ICR03391	SWU12838	29	C2 0115	ICR03107	5.21	2.22	0.92	0.34	0.27	0.00	-0.27
	13	SWU22374	HAU2857	29	C2_0115	ICR03107	5.44	3.06	0.48	0.41	0.22	-0.05	-0.17
	16	Gh56	NAU5120	30	BNL243	CER0168	5.82	2.34	0.84	0.35	0.21	0.07	-0.28
	28	SHIN0219	TMB2386	30	CER0168	SWU21718	5.80	3.20	0.61	0.41	0.24	-0.05	-0.19
	24	SWU13758	CGR5423	32	NAU2140	NAU2957	6.73	2.64	1.04	-0.37	0.08	-0.32	0.24
*t*3	1	**SWU10986**	**NAU2218**	1	CGR5663	NAU2343	5.78	4.12	0.00	-0.61	-0.01	0.04	-0.04
	1	**SWU10986**	**NAU2218**	2	SWU11950	TMB1268	6.19	4.28	0.11	-0.63	0.09	-0.14	0.04
	4	SWU12672	HAU1332	4	SWU21617	SWU11855	5.57	3.61	0.30	-0.57	-0.10	-0.13	0.23
	6	ICR03206	NAU896	6	ICR03206	NAU896	6.06	3.40	0.29	0.69	0.22	0.10	-0.32
	6	ICR03206	NAU896	7	NAU3181	SHIN0376	5.41	3.51	0.15	0.57	0.04	-0.16	0.12
	4	SWU12672	HAU1332	9	HAU190	HAU1618	5.07	3.17	0.32	0.53	0.21	0.03	-0.24
	7	SWU10067	SWU10064	14	SWU13909	TMB0071	6.56	4.67	0.19	0.66	0.19	-0.08	-0.11
	7	CGR5001	CGR6586	15	DC40183	DC40175	5.52	4.03	0.01	0.61	0.04	0.00	-0.04
	9	Gh158	DC40407	16	CGR6802	HAU1129	6.35	4.32	0.21	0.63	0.17	-0.16	-0.01
	12	DPL0303	COT107	16	SWU10062	SWU10094	5.54	3.63	0.26	0.58	0.13	0.09	-0.22
	13	PGML0014	CGR6732	17	SWU12818	CGR5576	5.53	3.72	0.29	-0.60	-0.21	0.23	-0.02
	11	NAU3390	NAU2460	17	NAU3765	SWU14627	6.32	4.37	0.26	-0.65	-0.16	-0.03	0.19
	7	**Gh474**	**SWU10785**	20	SWU20675	SWU20649	6.01	3.96	0.26	0.61	0.04	0.17	-0.21
	19	PGML4342	SWU14431	20	SWU20636	CGR6154	5.43	3.79	0.10	-0.59	-0.10	-0.02	0.13
	2	PGML0700	SWU12016	21	Gh451	SWU16489	8.92	6.08	0.11	-0.75	-0.15	0.04	0.11
	16	Gh56	NAU5120	21	BNL3171	CGR5808	8.99	5.95	0.40	0.74	0.27	-0.05	-0.23
	12	**HAU1316**	**NAU3519**	22	**SWU21635**	**SWU21646**	5.47	3.61	0.12	-0.58	-0.12	0.10	0.02
	19	DC40122	NAU833a	22	PGML1712	SWU21538	5.79	3.40	0.76	0.58	0.21	0.18	-0.38
	13	SWU22413	CGR5331	23	PGML4186	NAU3100	5.11	3.78	0.08	-0.59	0.12	-0.06	-0.06
	11	NAU3390	NAU2460	25	HAU1382	SWU19848	6.89	4.91	0.00	-0.68	0.01	0.02	-0.03
	16	SWU10214	Gh56	25	SWU19815	BNL3594	6.48	3.81	0.70	-0.59	-0.23	-0.13	0.36
	22	PGML0695	SWU20813	26	MGHES31	HAU1571	5.42	3.29	0.26	-0.59	-0.22	0.17	0.05
	13	SWU22374	HAU2857	29	C2 0115	ICR03107	5.30	3.58	0.19	0.59	0.12	-0.19	0.07
	28	TMB2386	SWU12343	30	BNL243	CER0168	5.28	3.36	0.20	0.56	0.16	-0.02	-0.14
	26	SWU0598	SWU18698	30	CER0168	SWU21718	5.15	3.25	0.29	0.56	0.25	-0.12	-0.12
*t*4	1	ICR03724	ICR03725	2	PGML0700	SWU12016	5.16	3.27	0.01	-0.56	0.02	0.04	-0.06
	1	NAU3384	CGR5663	8	DC20094	HAU1470b	5.02	2.59	0.52	-0.50	-0.26	0.29	-0.03
	1	HAU1417	NAU2437	9	Gh27	SWU15194	5.43	3.08	0.36	0.54	0.14	-0.26	0.13
	7	SWU10067	SWU10064	14	SWU13909	TMB0071	5.40	3.86	0.19	0.61	0.06	-0.19	0.13
	7	SWU10064	NAU3181	16	SWU10038	ICR00016	5.72	2.92	0.57	-0.61	0.10	0.26	-0.36
	1	SWU0077	HAU1417	19	NAU5330	Gh72	5.34	2.70	0.88	0.52	0.00	-0.36	0.36
	10	SWU20501b	CGR5873	20	SWU20700	CGR5548	5.42	2.43	1.28	-0.49	-0.12	0.49	-0.37
	2	PGML0700	SWU12016	21	Gh451	SWU16489	7.99	4.99	0.10	-0.70	0.09	0.04	-0.13
	1	NAU3384	CGR5663	21	SWU14431a	SWU15915	5.02	3.23	0.34	-0.57	0.19	0.06	-0.25
	16	Gh56	NAU5120	21	BNL3171	CGR5808	5.39	2.94	0.55	0.55	0.34	-0.22	-0.12
	11	NAU3390	NAU2460	25	HAU1382	SWU19848	5.83	3.99	0.26	-0.62	0.13	0.09	-0.22
	10	ICR00093	ICR07050	26	MGHES31	HAU1571	5.59	2.78	1.03	-0.52	0.05	0.36	-0.41

Stage	Chi	Flanking markers		Chj	Flanking markers		LOD	V(AA)	V(AAE)	AA	AAE1	AAE2	AAE3
	22	SWU21533	DPL0562	30	BNL243	CER0168	7.74	1.70	3.09	-0.42	-0.12	0.74	-0.62
	28	SWU12343	SWU14060	30	BNL243	CER0168	5.45	3.99	0.07	0.63	0.12	-0.07	-0.05
	16	Gh56	NAU5120	30	BNL243	CER0168	5.37	3.21	0.42	0.56	0.27	-0.19	-0.07
RILV population													
*t*1	1	SWU14514	Gh120	1	ICR03725	ICR03724	6.81	3.44	0.52	0.18	0.05	0.05	-0.10
	1	SWU11632	SWU21958	3	NAU2742	SWU12841	5.14	2.38	0.60	0.16	0.03	0.08	-0.11
	1	ICR03724	SWU11632	4	JESPR234	BNL530	5.84	2.09	0.98	-0.15	-0.02	-0.11	0.13
	5	Gh260	PGML0120	5	Gh260	PGML0120	8.83	3.81	1.26	0.21	0.00	0.15	-0.15
	5	Gh260	PGML0120	6	CIR291	CGR5883	6.10	2.82	0.67	0.17	0.01	0.10	-0.11
	4	BNL1167	JESPR234	6	SWU19656	CGR5124	5.56	2.36	0.73	0.15	0.04	0.08	-0.12
	6	MUSB1144	BNL3650	7	HAU1367	NAU3181	5.37	3.10	0.35	-0.18	-0.04	-0.04	0.08
	6	MUSB1144	BNL3650	8	DC20094	Gh197	5.12	1.62	1.07	-0.13	-0.04	-0.10	0.14
	1	ICR03725	ICR03724	8	HAU0810	TMB2904	5.55	2.31	0.60	0.15	0.01	0.09	-0.10
	1	ICR03724	SWU11632	10	SWU19932	HAU0635	6.27	2.24	0.83	0.15	0.04	0.09	-0.13
	9	CGR6876	BNL1317	12	C2 0115	SWU16858	6.40	2.84	0.72	-0.17	-0.09	-0.03	0.12
	6	BNL3650	TMB2940	12	C2 0115	SWU16858	7.91	3.33	0.93	-0.18	-0.08	-0.06	0.13
	2	CGR6695	SWU11013	12	ICR03107	HAU3373	5.52	2.71	0.58	0.17	0.02	0.08	-0.10
	12	BNL3261	Gh568	12	CGR6847	SWU17197	5.91	2.22	0.87	-0.15	-0.05	-0.08	0.13
	7	C2 0046	HAU1367	12	COT107	Gh243	5.23	1.69	0.92	0.13	0.08	0.06	-0.13
	1	DPL0790	ICR03725	12	DPL0400	HAU2173	5.88	2.52	0.72	-0.16	-0.08	-0.03	0.12
	8	HAU0810	TMB2904	12	DPL0400	HAU2173	6.42	3.06	0.66	-0.18	-0.04	-0.07	0.12
	12	C2 0115	SWU16858	13	CER0165	SWU13032	7.16	2.11	1.37	0.15	0.02	0.13	-0.15
	1	SWU14514	Gh120	13	SWU13032	HAU2850	5.34	2.44	0.63	0.16	0.04	0.07	-0.11
	6	MUSB1144	BNL3650	13	CGR5331	SHIN1462	7.07	2.97	1.12	0.17	0.01	0.12	-0.13
	12	Gh243	DPL0400	14	HAU0883	CIR228	7.63	3.17	0.82	0.18	0.05	0.08	-0.13
	1	ICR03725	ICR03724	14	DPL0502	ICR00401	6.69	2.22	1.04	0.15	0.06	0.09	-0.14
	2	TMB1268	SWU11976	14	DPL0502	ICR00401	5.79	2.69	0.65	0.17	0.05	0.07	-0.11
	14	HAU0883	CIR228	14	ICR01124	HAU2482	6.13	3.00	0.41	0.22	0.07	0.05	-0.12
	13	SWU13032	HAU2850	14	HAU2482	NAU4045	6.10	1.91	0.93	0.17	0.07	0.10	-0.17
	12	DPL0400	HAU2173	16	HAU3081	NAU747	5.66	1.92	1.15	0.14	0.02	0.12	-0.14
	1	SWU11632	SWU21958	17	HAU1413	CGR5576	5.51	2.38	0.73	0.16	0.02	0.09	-0.12
	4	SWU16783	SWU18876	18	SWU0738	ICR02849	7.98	3.88	0.84	0.20	0.04	0.09	-0.13
	15	NAU3736	SWU11691	18	SWU0738	ICR02849	5.65	1.55	1.48	0.13	-0.01	0.15	-0.14
	6	CGR5124	SWU19249	18	SWU0738	ICR02849	6.08	3.36	0.46	-0.18	-0.09	0.01	0.08
	2	DPL0217	CGR6695	19	NAU2816	PGML4342	6.02	2.44	1.15	0.16	0.01	0.13	-0.13
	17	HAU1413	CGR5576	19	NAU2816	PGML4342	8.21	3.34	0.91	0.18	0.03	0.10	-0.13
	14	ICR03105	ICR01124	19	PGML4342	NAU2894	8.15	3.66	0.91	0.24	0.04	0.12	-0.16
	15	NAU3736	SWU11691	19	SWU17789	SWU17882	6.09	2.86	0.60	-0.17	-0.03	-0.07	0.11
	14	DPL0502	ICR00401	20	SWU20246	Gh451	5.82	1.78	0.74	-0.17	-0.06	-0.10	0.16
	13	CER0165	SWU13032	21	BNL1552	CGR5148	6.72	3.12	0.84	0.18	0.06	0.07	-0.13
	4	SWU16783	SWU18876	21	CGR5748	PGML2500	5.65	2.48	0.77	0.16	0.07	0.06	-0.12
	10	NAU3395	CAU0234	21	CGR5748	PGML2500	5.56	2.94	0.68	-0.17	0.02	-0.11	0.09
	8	DC20094	Gh197	21	SWU16489	SWU16360	6.63	3.09	0.59	-0.18	-0.07	-0.04	0.11
	5	Gh260	PGML0120	21	SWU16408	BNL3171	5.24	2.11	0.54	0.14	0.05	0.06	-0.10
	6	BNL3650	TMB2940	21	BNL3171	HAU2937	5.26	2.09	0.66	0.14	0.03	0.08	-0.11
	21	SWU16489	SWU16360	23	CGR5158	HAU1758	11.6	5.28	1.25	-0.24	-0.06	-0.10	0.17
	1	Gh398	CGR6129	23	HAU0244	Gh327	8.54	3.43	1.14	-0.20	-0.10	-0.07	0.16
	12	Gh631	COT107	23	DC40286	PGML1434	5.78	2.13	0.73	-0.15	-0.09	-0.03	0.12
	17	HAU1413	CGR5576	23	NAU5373b	HAU2648	6.55	2.80	0.74	-0.17	-0.03	-0.09	0.12

Stage	Chi	Flanking markers		Chj	Flanking markers		LOD	V(AA)	V(AAE)	AA	AAE1	AAE2	AAE3
	1	DPL0790	ICR03725	24	Gh54	Gh454	5.37	2.51	0.55	-0.16	-0.08	-0.02	0.10
	6	MUSB1144	BNL3650	24	Gh54	Gh454	6.46	3.01	0.78	0.17	0.03	0.09	-0.12
	14	ICR03105	ICR01124	24	Gh454	HAU3076	5.69	2.53	0.69	0.20	0.03	0.11	-0.14
	23	HAU2648	SWU20501	25	HAU3012	SWU19676	10.0	4.21	1.71	0.21	0.05	0.13	-0.18
	6	BNL3650	TMB2940	25	DPL0377	SWU19413	6.94	3.16	0.67	-0.18	-0.05	-0.07	0.12
	2	DPL0217	CGR6695	25	SWU19412	NAU3112	5.46	2.57	0.57	0.16	0.05	0.05	-0.11
	17	SWU12838a	HAU1413	25	NAU4964	HAU1355	7.57	3.18	0.73	0.18	0.07	0.05	-0.12
	4	BNL530	SWU16781	25	BNL3098	HAU1224	5.72	2.34	0.71	0.15	0.05	0.07	-0.12
	6	CGR5124	SWU19249	26	CGR6477	PGML2562	5.23	1.58	0.80	-0.13	-0.06	-0.07	0.13
	12	BNL3261	Gh568	27	Gh247	CGR5867	5.58	2.27	1.03	0.15	0.02	0.11	-0.13
	10	HAU0635	PGML4154	28	BNL3545	PGML3983	7.33	3.13	0.97	0.18	0.06	0.08	-0.14
	16	HAU1129	C2_0011B	29	DPL0171	Gh499	5.25	2.49	0.61	0.16	0.08	0.02	-0.10
	2	CGR6695	SWU11013	29	DPL0171	Gh499	6.32	3.20	0.64	-0.18	-0.05	-0.07	0.11
	26	CGR6477	PGML2562	29	DPL0171	Gh499	7.12	1.97	1.20	0.14	0.01	0.13	-0.14
	4	SWU16783	SWU18876	30	TMB1638	CGR6812	5.82	2.81	0.59	-0.17	-0.03	-0.08	0.11
	16	HAU3081	NAU747	31	DPL0057	NAU3109	6.45	3.08	0.66	-0.18	-0.03	-0.08	0.11
	2	SWU12490	DPL0200	31	CGR6772	HAU0355	5.30	2.04	0.86	-0.14	-0.06	-0.07	0.13
	9	CGR6876	BNL1317	31	SWU16780	SWU16735	5.59	2.41	0.88	0.15	-0.02	0.12	-0.10
	23	PGML1434	MUSB994	31	SWU16780	SWU16735	5.08	2.63	0.39	0.16	0.05	0.04	-0.09
	30	TMB1638	CGR6812	31	SWU16730	SWU16721	5.87	2.27	0.82	-0.15	-0.04	-0.09	0.13
	13	CGR5331	SHIN1462	32	HAU1000	TMB1931	7.78	3.27	0.96	0.18	0.04	0.09	-0.14
	12	HAU3373	CGR6847	33	BNL3661	PGML4891	6.03	2.63	0.56	0.16	0.07	0.04	-0.11
	31	DPL0057	NAU3109	33	BNL3661	PGML4891	5.47	2.88	0.47	0.17	0.03	0.07	-0.10
	12	HAU3373	CGR6847	34	JESPR297	ICR00647	5.81	3.21	0.44	-0.18	-0.04	-0.06	0.09
	25	BNL3098	HAU1224	34	JESPR297	ICR00647	8.04	3.64	0.95	-0.19	-0.05	-0.08	0.14
	1	Gh398	CGR6129	35	NAU2139	TMB1152	9.93	4.80	1.00	-0.22	-0.06	-0.08	0.14
	9	CGR5009	CGR6771	36	CGR5548	SWU20700	5.45	2.52	0.67	0.16	0.05	0.07	-0.12
	26	CGR6477	PGML2562	36	CGR5548	SWU20700	8.35	3.59	0.94	-0.19	-0.11	-0.03	0.13
	1	DPL0790	ICR03725	36	CGR5548	SWU20700	5.09	1.95	0.74	0.14	0.09	0.04	-0.12
	23	NAU5373b	HAU2648	37	DPL0131	DPL0777	8.31	3.55	1.09	-0.19	-0.03	-0.11	0.14
	25	SWU19676	NAU2968	37	DPL0131	DPL0777	6.78	3.12	0.84	0.18	0.04	0.09	-0.13
	19	NAU2816	PGML4342	37	DPL0131	DPL0777	5.69	2.52	0.65	0.16	0.07	0.04	-0.11
	9	SWU15413	NAU0483	37	DPL0777	HAU0423	7.19	3.65	0.53	0.19	0.05	0.06	-0.11
	37	DPL0131	DPL0777	38	NAU2450	PGML1942	7.27	2.76	1.06	0.17	0.07	0.08	-0.15
	10	SWU19932	HAU0635	38	NAU2450	PGML1942	5.20	1.55	0.85	-0.13	-0.06	-0.07	0.13
	23	SHIN0272	NAU2140	39	HAU2022	BNL0827	7.19	2.01	1.65	-0.16	-0.05	-0.14	0.19
	33	BNL3661	PGML4891	39	HAU2022	BNL0827	5.12	2.11	0.86	0.15	0.04	0.09	-0.13
	36	SWU20658	CGR6154	39	HAU2022	BNL0827	6.21	2.92	0.59	0.17	0.05	0.06	-0.11
	5	Gh260	PGML0120	39	NAU5480	DPL0270	6.30	2.35	1.28	-0.16	0.00	-0.14	0.14
	26	HAU1571	CGR6477	39	NAU5480	DPL0270	6.33	2.59	0.81	0.16	0.03	0.09	-0.12
	1	Gh120	Gh398	39	NAU5480	DPL0270	5.20	2.07	0.69	-0.14	-0.03	-0.08	0.11
	21	SWU16493	SWU16489	39	DPL0270	SWU16437	8.00	2.95	1.03	-0.17	-0.06	-0.08	0.14
	29	DPL0171	Gh499	39	DPL0270	SWU16437	7.52	2.46	1.15	-0.16	-0.08	-0.07	0.15
	3	NAU2742	SWU12841	39	DPL0270	SWU16437	8.50	3.80	0.74	0.20	0.03	0.09	-0.12
t2	1	SWU14514	Gh120	1	ICR03295	SWU10912	6.58	3.53	0.42	0.63	-0.01	0.25	-0.25
	1	DPL0790	ICR03725	2	DPL0217	CGR6695	5.82	2.24	0.82	0.50	0.15	0.27	-0.42
	5	Gh260	PGML0120	5	Gh260	PGML0120	10.4	5.53	0.49	0.83	0.12	0.23	-0.35
	4	BNL1167	JESPR234	6	MUSB1144	BNL3650	5.02	2.90	0.29	0.55	0.02	0.20	-0.22
	5	Gh260	PGML0120	6	CIR291	CGR5883	6.34	3.74	0.37	0.63	0.00	0.24	-0.25

Stage	Chi	Flanking markers		Chj	Flanking markers		LOD	V(AA)	V(AAE)	AA	AAE1	AAE2	AAE3
	6	MUSB1144	BNL3650	8	DC20094	Gh197	6.88	3.43	0.57	-0.60	-0.03	-0.28	0.32
	1	ICR03725	ICR03724	10	SWU19932	HAU0635	5.00	2.51	0.34	0.52	0.14	0.13	-0.27
	12	BNL3261	Gh568	12	CGR6847	SWU17197	5.38	2.68	0.43	-0.55	0.01	-0.27	0.26
	1	DPL0790	ICR03725	12	DPL0400	HAU2173	6.03	3.49	0.16	-0.62	-0.06	-0.12	0.18
	8	HAU0810	TMB2904	12	DPL0400	HAU2173	7.71	4.27	0.35	-0.68	-0.08	-0.20	0.28
	6	MUSB1144	BNL3650	13	CGR5331	SHIN1462	6.70	3.33	0.50	0.60	0.08	0.24	-0.31
	12	Gh243	DPL0400	14	HAU0883	CIR228	6.62	3.70	0.25	0.63	0.12	0.12	-0.23
	1	ICR03725	ICR03724	14	DPL0502	ICR00401	7.35	3.16	0.78	0.58	0.16	0.24	-0.41
	10	NAU3395	CAU0234	14	ICR03943	ICR12281	5.54	2.92	0.35	-0.58	0.00	-0.24	0.24
	14	ICR03943	ICR12281	14	ICR03943	ICR12281	8.25	3.72	0.60	0.76	0.21	0.20	-0.41
	12	DPL0400	HAU2173	16	HAU3081	NAU747	6.52	3.17	0.75	0.58	-0.03	0.36	-0.33
	2	TMB1268	SWU11976	16	NAU747	HAU1129	5.71	2.95	0.47	0.56	-0.02	0.28	-0.26
	12	ICR03107	HAU3373	17	NAU3995	SWU12838a	6.77	3.30	1.08	0.59	-0.21	0.48	-0.27
	4	SWU16783	SWU18876	18	SWU0738	ICR02849	9.63	4.89	0.64	0.72	0.10	0.26	-0.36
	15	NAU3736	SWU11691	18	SWU0738	ICR02849	5.55	2.98	0.24	0.57	0.08	0.14	-0.22
	17	HAU1413	CGR5576	19	NAU2816	PGML4342	9.01	4.84	0.36	0.71	0.09	0.18	-0.27
	10	SWU19932	HAU0635	20	HAU1314	SWU20035	5.44	2.64	0.37	-0.53	-0.16	-0.12	0.28
	13	CGR5331	SHIN1462	20	SWU20027	Gh187	6.34	3.20	0.52	0.59	-0.01	0.29	-0.28
	6	SWU19656	CGR5124	21	CGR5148	SHIN0337	5.48	3.31	0.15	0.59	0.07	0.11	-0.17
	14	ICR00401	ICR03105	21	CGR5148	SHIN0337	5.82	2.61	0.45	0.63	0.12	0.24	-0.36
	4	SWU16783	SWU18876	21	CGR5748	PGML2500	6.17	3.06	0.49	0.58	0.12	0.19	-0.31
	8	DC20094	Gh197	21	SWU16489	SWU16360	10.0	4.59	0.90	-0.71	-0.11	-0.31	0.43
	13	CER0165	SWU13032	21	SWU16489	SWU16360	6.43	3.03	0.87	0.58	-0.06	0.40	-0.35
	12	Gh631	COT107	23	DC40286	PGML1434	6.51	3.33	0.46	-0.61	-0.02	-0.27	0.29
	21	PGML2500	CGR6521	23	PGML1434	MUSB994	5.99	2.83	0.60	0.55	0.05	0.28	-0.33
	1	ICR03724	SWU11632	23	HAU2648	SWU20501	5.82	3.03	0.32	-0.58	-0.18	-0.08	0.26
	1	DPL0790	ICR03725	24	PGML1207	Gh54	7.12	3.98	0.42	-0.67	0.02	-0.28	0.25
	24	Gh54	Gh454	24	CGR6079	SWU13100	5.56	2.65	0.53	0.60	0.08	0.27	-0.35
	15	NAU3736	SWU11691	25	SWU19412	NAU3112	5.86	2.46	0.61	0.52	0.08	0.27	-0.35
	17	SWU12838a	HAU1413	25	NAU4964	HAU1355	12.6	6.13	0.83	0.81	0.09	0.31	-0.40
	12	HAU3373	CGR6847	26	HAU1571	CGR6477	5.17	2.79	0.47	0.55	-0.03	0.29	-0.25
	10	NAU3395	CAU0234	26	CGR6477	PGML2562	5.95	2.86	0.54	0.55	0.05	0.27	-0.32
	24	Gh54	Gh454	27	Gh247	CGR5867	6.38	3.02	0.61	0.57	0.12	0.24	-0.36
	16	HAU1129	C2 0011B	29	DPL0171	Gh499	5.48	2.61	0.44	0.52	0.13	0.18	-0.31
	1	SWU21958	NAU0748	29	DPL0171	Gh499	6.87	3.26	0.81	-0.59	0.05	-0.38	0.33
	12	CGR6847	SWU17197	30	TMB1638	CGR6812	5.70	2.67	0.34	-0.54	-0.22	-0.03	0.25
	25	NAU4964	HAU1355	30	TMB1638	CGR6812	6.68	3.22	0.42	-0.59	-0.24	-0.05	0.29
	18	SWU21718	SWU0738	31	SWU16735	SWU16755	5.20	2.34	0.60	0.50	0.04	0.29	-0.33
	30	TMB1638	CGR6812	31	SWU16730	SWU16721	7.93	3.23	1.18	-0.59	0.00	-0.44	0.44
	21	SWU16493	SWU16489	33	BNL3661	PGML4891	6.36	3.27	0.50	0.60	-0.03	0.30	-0.27
	25	NAU4964	HAU1355	34	JESPR297	ICR00647	7.43	3.16	0.88	-0.57	-0.11	-0.31	0.42
	1	Gh398	CGR6129	35	NAU2139	TMB1152	8.58	4.79	0.36	-0.72	-0.03	-0.22	0.26
	4	JESPR234	BNL530	36	CGR5548	SWU20700	5.17	2.51	0.56	0.52	0.00	0.30	-0.30
	26	CGR6477	PGML2562	36	CGR5548	SWU20700	7.97	4.40	0.40	-0.70	-0.07	-0.21	0.28
	25	SWU19676	NAU2968	36	CGR5548	SWU20700	6.38	3.39	0.41	0.61	0.06	0.22	-0.28
	18	SWU0738	ICR02849	36	SWU20658	CGR6154	6.44	3.08	0.73	-0.58	0.07	-0.37	0.30
	32	TMB0071	HAU1000	36	SWU20658	CGR6154	5.37	2.88	0.39	-0.56	0.02	-0.26	0.24
	23	NAU5373b	HAU2648	37	DPL0131	DPL0777	11.4	5.47	1.52	-0.77	0.07	-0.53	0.46
	32	HAU1000	TMB1931	37	DPL0131	DPL0777	5.25	1.67	1.23	0.43	-0.09	0.49	-0.40

Stage	Chi	Flanking markers		Chj	Flanking markers		LOD	V(AA)	V(AAE)	AA	AAE1	AAE2	AAE3
	19	NAU2816	PGML4342	37	DPL0777	HAU0423	5.83	3.02	0.37	0.56	0.08	0.20	-0.27
	37	DPL0131	DPL0777	38	NAU2450	PGML1942	5.83	2.98	0.49	0.57	-0.04	0.30	-0.26
	36	SWU20658	CGR6154	39	HAU2022	BNL0827	5.12	3.25	0.14	0.59	0.09	0.09	-0.18
	1	Gh120	Gh398	39	NAU5480	DPL0270	6.00	3.28	0.32	-0.59	-0.07	-0.18	0.25
	21	SWU16493	SWU16489	39	DPL0270	SWU16437	7.15	3.78	0.50	-0.64	-0.03	-0.27	0.30
	3	NAU2742	SWU12841	39	DPL0270	SWU16437	5.98	3.24	0.30	0.59	0.08	0.17	-0.25
	29	DPL0171	Gh499	39	DPL0270	SWU16437	7.01	3.35	0.68	-0.60	-0.01	-0.32	0.34
	31	SWU16755	SWU16732	39	SWU16437	SWU16432	5.44	2.60	0.37	0.53	0.08	0.19	-0.27
*t*3	1	SWU14514	Gh120	1	ICR03295	SWU10912	5.33	3.70	0.23	0.91	-0.26	-0.04	0.30
	5	Gh260	PGML0120	5	Gh260	PGML0120	7.84	5.45	0.35	1.16	-0.20	-0.20	0.40
	5	Gh260	PGML0120	6	CIR291	CGR5883	5.15	4.12	0.22	0.95	-0.27	0.06	0.21
	1	HAU2489	DPL0790	6	SWU19656	CGR5124	5.73	3.20	0.72	-0.85	0.37	0.20	-0.58
	2	TMB1268	SWU11976	6	SWU19656	CGR5124	9.03	5.35	1.22	1.07	-0.52	-0.17	0.69
	6	MUSB1144	BNL3650	8	DC20094	Gh197	8.80	5.77	0.45	-1.11	0.40	-0.03	-0.36
	3	NAU2742	SWU12841	8	HAU0810	TMB2904	5.96	3.43	0.68	-0.86	0.46	0.01	-0.47
	2	CGR6695	SWU11013	12	CGR6847	SWU17197	5.12	3.37	0.49	0.85	-0.28	-0.16	0.44
	12	BNL3261	Gh568	12	Gh243	DPL0400	5.39	3.14	0.69	-0.83	0.45	0.05	-0.50
	1	DPL0790	ICR03725	12	DPL0400	HAU2173	6.15	4.29	0.32	-0.98	0.30	0.04	-0.34
	10	SWU13030	NAU4967	12	DPL0400	HAU2173	6.01	4.06	0.54	0.94	-0.45	0.08	0.37
	8	HAU0810	TMB2904	12	DPL0400	HAU2173	8.50	6.00	0.28	-1.14	0.35	-0.17	-0.17
	6	MUSB1144	BNL3650	13	CGR5331	SHIN1462	5.35	3.95	0.10	0.92	-0.21	0.09	0.13
	6	SWU19656	CGR5124	14	HAU0883	CIR228	6.14	4.34	0.25	0.96	-0.32	0.11	0.21
	4	BNL1167	JESPR234	14	DPL0502	ICR00401	5.22	3.73	0.13	-0.90	0.21	-0.03	-0.19
	1	Gh529	SWU17434	14	HAU2482	NAU4045	5.60	3.82	0.24	-1.08	0.37	-0.19	-0.18
	12	CGR6847	SWU17197	14	NAU4045	ICR03943	5.42	3.89	0.29	0.98	-0.32	-0.01	0.33
	8	HAU0810	TMB2904	14	ICR03943	ICR12281	5.14	3.12	0.58	-0.88	0.41	0.09	-0.50
	14	ICR03943	ICR12281	14	ICR03943	ICR12281	6.43	4.21	0.03	1.17	-0.17	0.01	0.16
	12	DPL0400	HAU2173	16	HAU3081	NAU747	8.85	5.85	0.59	1.12	-0.50	0.19	0.30
	2	TMB1268	SWU11976	16	NAU747	HAU1129	8.25	5.26	0.77	1.06	-0.46	-0.07	0.53
	5	Gh260	PGML0120	17	SWU12838a	HAU1413	5.14	3.78	0.12	-0.90	0.21	-0.04	-0.18
	4	SWU16783	SWU18876	18	SWU0738	ICR02849	6.50	4.59	0.10	1.00	-0.22	0.07	0.15
	15	NAU3736	SWU11691	18	SWU0738	ICR02849	6.62	3.95	0.64	0.92	-0.39	-0.10	0.50
	17	HAU1413	CGR5576	19	NAU2816	PGML4342	5.50	4.12	0.09	0.93	-0.18	0.10	0.07
	10	SWU19932	HAU0635	20	HAU1314	SWU20035	6.37	4.29	0.41	-0.96	0.30	0.10	-0.40
	12	HAU3373	CGR6847	20	SWU20027	Gh187	5.99	3.94	0.46	-0.92	0.38	0.00	-0.38
	13	CGR5331	SHIN1462	20	SWU20027	Gh187	5.58	3.81	0.19	0.91	-0.28	0.12	0.16
	6	SWU19656	CGR5124	21	CGR5148	SHIN0337	6.55	4.68	0.35	1.00	-0.32	-0.02	0.34
	8	DC20094	Gh197	21	SWU16489	SWU16360	7.43	5.36	0.16	-1.08	0.25	-0.04	-0.20
	16	HAU3081	NAU747	21	SWU16361	SWU16408	5.14	2.61	0.63	0.75	-0.52	0.22	0.30
	2	SWU11976	SWU12001	21	BNL3171	HAU2937	5.02	3.37	0.33	0.85	-0.35	0.05	0.30
	12	DPL0400	HAU2173	22	BNL4030	NAU2026	5.76	2.93	0.85	0.80	-0.56	0.07	0.49
	12	SWU17197	Gh631	23	DC40286	PGML1434	5.43	4.23	0.12	-0.96	0.17	0.06	-0.23
	2	DPL0217	CGR6695	23	PGML1434	MUSB994	6.93	3.85	0.87	0.91	-0.54	0.01	0.53
	1	SWU14616	SWU14077	23	HAU2648	SWU20501	5.93	4.54	0.23	0.99	-0.23	-0.08	0.30
	23	MUSB994	NAU2238	23	HAU2648	SWU20501	5.92	3.60	0.73	-0.88	0.37	0.18	-0.55
	1	DPL0790	ICR03725	24	PGML1207	Gh54	6.05	4.22	0.43	-0.98	0.22	0.22	-0.44
	24	Gh54	Gh454	24	HAU3076	SWU13121	6.19	4.16	0.42	1.12	-0.39	0.01	0.37
	17	SWU12838a	HAU1413	25	NAU4964	HAU1355	13.6	8.31	0.81	1.34	-0.54	0.03	0.50
	12	HAU3373	CGR6847	26	MGHES31	HAU1571	5.06	2.92	0.50	0.80	-0.45	0.14	0.31

Stage	Chi	Flanking markers		Chj	Flanking markers		LOD	V(AA)	V(AAE)	AA	AAE1	AAE2	AAE3
	10	HAU0635	PGML4154	28	BNL3545	PGML3983	5.49	3.59	0.34	0.88	-0.32	-0.03	0.34
	26	CGR6477	PGML2562	29	DPL0171	Gh499	6.70	3.85	0.65	0.91	-0.45	-0.01	0.46
	1	NAU0748	NAU2697	29	Gh111	Gh27	5.37	3.63	0.30	-0.88	0.34	-0.06	-0.28
	12	CGR6847	SWU17197	30	TMB1638	CGR6812	5.62	4.15	0.19	-0.95	0.14	0.15	-0.29
	6	SWU19656	CGR5124	30	TMB1638	CGR6812	6.19	3.81	0.43	0.90	-0.32	-0.08	0.40
	21	BNL3171	HAU2937	30	TMB1638	CGR6812	5.02	3.14	0.27	-0.83	0.31	0.01	-0.32
	6	CGR5355	SWU19656	31	CGR6772	HAU0355	5.33	3.15	0.67	0.82	-0.36	-0.16	0.52
	1	CGR6129	ICR03295	31	SWU16780	SWU16735	5.86	4.27	0.45	0.95	-0.26	-0.17	0.43
	30	TMB1638	CGR6812	31	SWU16730	SWU16721	6.44	4.29	0.10	-0.97	0.21	-0.02	-0.20
	21	SWU16493	SWU16489	33	BNL3661	PGML4891	7.77	4.42	0.65	0.98	-0.52	0.18	0.34
	12	HAU3373	CGR6847	34	JESPR297	ICR00647	5.40	3.26	0.40	-0.85	0.34	0.05	-0.39
	25	BNL3098	HAU1224	34	JESPR297	ICR00647	6.22	4.20	0.30	-0.93	0.33	-0.24	-0.09
	1	Gh120	Gh398	35	NAU2139	TMB1152	8.07	5.59	0.41	-1.10	0.29	0.12	-0.41
	7	CGR5372	C2 0046	36	CGR5548	SWU20700	5.14	2.85	0.73	0.80	-0.42	-0.15	0.57
	8	HAU0810	TMB2904	36	CGR5548	SWU20700	5.10	3.60	0.27	-0.89	0.12	0.21	-0.33
	32	TMB0071	HAU1000	36	CER0167	SWU20658	5.67	3.70	0.29	-0.89	0.32	-0.04	-0.29
	18	SWU0738	ICR02849	36	SWU20658	CGR6154	5.22	3.59	0.28	-0.88	0.33	-0.06	-0.27
	33	BNL3661	PGML4891	36	SWU20658	CGR6154	6.16	3.47	0.73	0.87	-0.50	0.02	0.48
	10	NAU3395	CAU0234	36	SWU20658	CGR6154	5.29	3.44	0.29	-0.86	0.33	-0.06	-0.27
	23	NAU5373b	HAU2648	37	DPL0131	DPL0777	10.3	6.97	0.61	-1.23	0.52	-0.23	-0.29
	25	NAU2968	DPL0377	37	DPL0131	DPL0777	5.49	3.07	0.90	0.82	-0.08	-0.50	0.58
	2	SWU11976	SWU12001	37	BNL5602	JESPR251	5.37	3.84	0.27	-0.91	0.29	0.01	-0.30
	24	PGML1207	Gh54	37	BNL5602	JESPR251	5.33	3.30	0.66	-0.85	0.34	0.20	-0.54
	10	HAU0635	PGML4154	38	NAU2450	PGML1942	8.28	5.16	0.65	1.05	-0.48	0.05	0.43
	37	DPL0131	DPL0777	38	NAU2450	PGML1942	5.59	3.83	0.36	0.92	-0.34	-0.01	0.35
	1	SWU14514	Gh120	39	NAU5480	DPL0270	6.08	3.81	0.47	-0.90	0.32	0.11	-0.43
	21	CGR6521	Gh450	39	NAU5480	DPL0270	7.64	5.61	0.13	-1.10	0.24	-0.10	-0.14
	29	DPL0171	Gh499	39	DPL0270	SWU16437	9.24	5.94	0.78	-1.13	0.53	-0.06	-0.47
	3	NAU2742	SWU12841	39	DPL0270	SWU16437	5.50	3.91	0.11	0.92	-0.20	0.03	0.17
*t*4	1	SWU14514	Gh120	1	ICR03295	SWU10912	5.02	2.99	0.59	0.84	-0.43	-0.05	0.49
	1	DPL0790	ICR03725	2	DPL0217	CGR6695	5.01	3.28	0.20	0.88	-0.30	0.21	0.10
	5	Gh260	PGML0120	5	Gh260	PGML0120	7.71	4.76	0.71	1.13	-0.47	-0.08	0.55
	1	DPL0790	ICR03725	6	CGR5355	SWU19656	5.58	3.21	1.02	-0.88	0.48	0.21	-0.69
	2	TMB1268	SWU11976	6	SWU19656	CGR5124	8.68	4.94	1.19	1.06	-0.57	-0.12	0.69
	6	MUSB1144	BNL3650	8	DC20094	Gh197	8.27	4.92	0.72	-1.06	0.55	-0.13	-0.42
	3	NAU2742	SWU12841	8	HAU0810	TMB2904	5.74	2.41	0.89	-0.74	0.62	-0.19	-0.44
	3	SWU12840	NAU2742	12	ICR03107	HAU3373	5.55	2.89	0.93	0.82	-0.57	0.00	0.58
	6	CGR6749	NAU3186	12	HAU3373	CGR6847	5.14	2.59	0.99	0.77	-0.48	-0.16	0.64
	1	NAU2697	SWU0320	12	CGR6847	SWU17197	5.28	3.25	0.33	-0.86	0.34	-0.01	-0.33
	10	SWU13030	NAU4967	12	DPL0400	HAU2173	8.98	4.09	1.39	0.97	-0.74	0.10	0.63
	8	HAU0810	TMB2904	12	DPL0400	HAU2173	7.48	4.87	0.47	-1.06	0.45	-0.11	-0.34
	6	SWU19656	CGR5124	14	HAU0883	CIR228	7.18	4.69	0.44	1.03	-0.44	0.16	0.28
	4	BNL1167	JESPR234	14	DPL0502	ICR00401	5.21	3.53	0.23	-0.90	0.32	-0.13	-0.18
	1	Gh529	SWU17434	14	HAU2482	NAU4045	6.23	3.64	0.56	-1.11	0.59	-0.23	-0.36
	9	MUSS139	PGML2830	14	HAU2482	NAU4045	5.27	2.51	0.87	0.92	-0.74	0.16	0.58
	12	DPL0400	HAU2173	14	HAU2482	NAU4045	5.97	3.22	0.62	1.05	-0.61	0.09	0.53
	8	HAU0810	TMB2904	14	ICR03943	ICR12281	5.66	2.99	0.82	-0.90	0.58	-0.03	-0.55
	14	ICR03943	ICR12281	14	ICR03943	ICR12281	5.78	3.45	0.43	1.06	-0.50	-0.03	0.53
	12	DPL0400	HAU2173	16	HAU3081	NAU747	10.8	6.66	0.94	1.23	-0.64	0.18	0.46

Stage	Chi	Flanking markers		Chj	Flanking markers		LOD	V(AA)	V(AAE)	AA	AAE1	AAE2	AAE3
	2	TMB1268	SWU11976	16	NAU747	HAU1129	8.29	4.55	1.12	1.02	-0.60	-0.04	0.64
	4	SWU16783	SWU18876	18	SWU0738	ICR02849	5.05	3.05	0.41	0.84	-0.42	0.10	0.32
	15	NAU3736	SWU11691	18	SWU0738	ICR02849	6.23	3.21	1.07	0.86	-0.53	-0.13	0.66
	17	HAU1413	CGR5576	19	NAU2816	PGML4342	5.12	3.64	0.16	0.90	-0.25	0.08	0.17
	10	SWU19932	HAU0635	20	HAU1314	SWU20035	6.75	4.46	0.47	-1.01	0.35	0.09	-0.44
	12	HAU3373	CGR6847	20	SWU20027	Gh187	5.31	2.84	0.65	-0.80	0.48	-0.01	-0.47
	13	CGR5331	SHIN1462	20	SWU20027	Gh187	5.50	2.98	0.53	0.83	-0.49	0.28	0.21
	6	SWU19656	CGR5124	21	CGR5148	SHIN0337	5.20	2.82	0.54	0.80	-0.44	0.03	0.41
	10	SWU19932	HAU0635	21	CGR5148	SHIN0337	5.06	2.91	0.49	0.82	-0.35	-0.10	0.45
	8	DC20094	Gh197	21	SWU16489	SWU16360	7.82	4.83	0.56	-1.06	0.50	-0.13	-0.37
	16	HAU3081	NAU747	21	SWU16361	SWU16408	5.43	2.27	1.26	0.72	-0.68	0.05	0.63
	12	DPL0400	HAU2173	22	BNL4030	NAU2026	7.46	2.89	1.24	0.82	-0.73	0.18	0.55
	16	HAU1129	C2 0011B	23	DC40286	PGML1434	5.71	3.51	0.56	0.89	-0.47	0.08	0.40
	2	DPL0217	CGR6695	23	PGML1434	MUSB994	5.68	2.70	1.00	0.78	-0.62	0.08	0.54
	1	SWU14616	SWU14077	23	HAU2648	SWU20501	6.23	3.79	0.66	0.93	-0.45	-0.05	0.50
	24	Gh54	Gh454	24	HAU3076	SWU13121	6.43	3.76	0.58	1.09	-0.61	0.03	0.58
	3	NAU2742	SWU12841	25	NAU4964	HAU1355	5.20	2.31	0.79	0.73	-0.59	0.17	0.42
	17	SWU12838a	HAU1413	25	NAU4964	HAU1355	14.0	6.92	1.51	1.27	-0.81	0.21	0.60
	12	HAU3373	CGR6847	26	HAU1571	CGR6477	5.80	2.76	1.17	0.79	-0.60	-0.06	0.66
	10	HAU0635	PGML4154	28	BNL3545	PGML3983	7.43	4.14	0.97	0.97	-0.55	-0.06	0.61
	26	CGR6477	PGML2562	29	DPL0171	Gh499	7.47	3.41	1.12	0.88	-0.57	-0.09	0.66
	6	BNL3650	TMB2940	29	Gh111	Gh27	5.59	3.03	0.67	0.83	-0.51	0.06	0.45
	25	NAU4964	HAU1355	30	TMB1638	CGR6812	5.28	3.24	0.32	-0.88	0.37	-0.05	-0.32
	1	CGR6129	ICR03295	31	SWU16780	SWU16735	5.25	3.32	0.57	0.87	-0.41	-0.06	0.47
	16	CGR5506	CGR5925	31	SWU16735	SWU16755	5.60	3.21	0.58	-0.86	0.50	-0.15	-0.35
	30	TMB1638	CGR6812	31	SWU16730	SWU16721	6.20	3.86	0.46	-0.95	0.35	0.10	-0.45
	13	NAU3398	CGR5331	33	BNL3661	PGML4891	5.34	2.78	0.67	-0.80	0.54	-0.15	-0.39
	21	SWU16493	SWU16489	33	BNL3661	PGML4891	8.01	3.40	1.33	0.89	-0.72	0.09	0.63
	25	BNL3098	HAU1224	34	JESPR297	ICR00647	5.14	3.08	0.45	-0.83	0.44	-0.18	-0.26
	10	SWU19932	HAU0635	35	NAU2139	TMB1152	5.33	2.71	0.66	-0.83	0.55	-0.30	-0.25
	1	Gh398	CGR6129	35	NAU2139	TMB1152	7.98	4.39	1.32	-1.01	0.54	0.21	-0.75
	12	CGR6847	SWU17197	35	NAU2139	TMB1152	6.36	2.66	1.11	0.79	-0.72	0.30	0.42
	7	CGR5372	C2_0046	36	CGR5548	SWU20700	6.35	3.60	0.94	0.93	-0.49	-0.15	0.65
	25	SWU19676	NAU2968	36	CGR5548	SWU20700	6.91	4.35	0.63	1.00	-0.43	-0.06	0.50
	21	BNL1552	CGR5148	36	CER0167	SWU20658	5.03	2.35	0.86	0.74	-0.54	-0.03	0.57
	32	TMB0071	HAU1000	36	CER0167	SWU20658	6.63	3.34	0.93	-0.88	0.62	-0.14	-0.49
	33	BNL3661	PGML4891	36	SWU20658	CGR6154	6.99	3.30	1.04	0.87	-0.66	0.13	0.53
	23	NAU5373b	HAU2648	37	DPL0131	DPL0777	10.2	5.74	1.22	-1.16	0.73	-0.19	-0.54
	25	NAU2968	DPL0377	37	DPL0131	DPL0777	5.52	2.61	1.37	0.78	-0.29	-0.50	0.79
	21	CGR5748	PGML2500	37	DPL0131	DPL0777	5.21	2.96	0.63	0.83	-0.47	0.00	0.46
	10	HAU0635	PGML4154	38	NAU2450	PGML1942	7.11	3.60	0.86	0.91	-0.57	0.06	0.51
	37	DPL0131	DPL0777	38	NAU2450	PGML1942	6.55	3.57	0.92	0.91	-0.52	-0.09	0.61
	31	CGR6772	HAU0355	38	NAU2450	PGML1942	5.44	2.76	0.56	0.79	-0.49	0.16	0.33
	1	SWU14514	Gh120	39	NAU5480	DPL0270	5.84	3.43	0.67	-0.88	0.42	0.09	-0.51
	21	CGR6521	Gh450	39	NAU5480	DPL0270	6.15	3.90	0.40	-0.95	0.42	-0.13	-0.28
	29	Gh111	Gh27	39	DPL0270	SWU16437	7.72	3.52	1.04	-0.90	0.65	-0.12	-0.53
	3	NAU2742	SWU12841	39	DPL0270	SWU16437	6.34	3.43	0.70	0.89	-0.53	0.08	0.45

Chi and Chj represent the linkage group number of the loci being tested in the analysis

Flanking markers in bold are those flanking M-QTLs identified by inclusive composite interval mapping in additional table S5

AA is the epistatic effect between loci i and j

AAE is the effect of the environmental interaction of epistasis

AAEl, AAE2 and AAE3 indicate the epistatic effects of QTL × environment interactions in E1, E2 and E3, respectively

V(AA)% and V(AAE)%, percentage of the total variation explained by the AA and AAE

Table S9 Epistatic effects and environmental interactions detected for boll number per plant in BCF_1 and $BCVF_1$ populations using two-locus analysis by inclusive composite interval mapping

Stage	Chi	Flanking markers		Chj	Flanking markers		LOD	V(AA)	V(AAE)	AA	AAE1	AAE2	AAE3
						BCF_1 population							
*t*1	1	BNL2827a	NAU6367	1	NAU3384	CGR5663	5.93	2.31	0.00	-0.18	-0.09	-0.03	0.12
	1	NAU3177	ICR03724	2	SWU11887	SWU11976	10.1	5.26	1.49	-0.18	-0.12	0.02	0.10
	1	SWU11191	BNL2827b	7	SWU10064	NAU3181	5.44	2.20	1.39	0.11	0.12	-0.04	-0.08
	4	ICR01729	SWU16781	9	NAU1282	CGR6771	5.07	2.68	0.71	0.13	0.06	0.03	-0.09
	1	BNL2827a	NAU6367	11	NAU3390	NAU2460	5.22	2.23	1.42	-0.12	-0.11	-0.01	0.12
	11	NAU3390	NAU2460	11	NAU1014	ICR10344	5.49	2.15	1.44	-0.11	-0.07	-0.06	0.13
	7	CER0036	PGML1916	14	SWU14545	SWU14543	5.80	0.93	2.67	0.07	0.12	0.05	-0.17
	9	NAU5474	Gh158	16	NAU862	CGR6802	5.82	1.97	1.58	0.11	0.10	0.03	-0.13
	12	DPL0303	COT107	17	NAU3765	SWU14627	5.28	1.72	1.24	-0.10	-0.04	-0.08	0.12
	14	SWU14545	SWU14543	18	SWU22281	SWU21800	5.35	3.06	0.23	-0.13	-0.03	-0.03	0.05
	10	HAU0635	NAU2139	18	CIR099	NAU748	5.51	1.97	1.20	0.11	0.04	0.08	-0.12
	9	HAU1618	NAU2873	18	NAU748	SWU22192	7.36	1.81	2.17	-0.10	-0.06	-0.10	0.16
	1	CGR6129	DPL0790	20	SWU20246	SWU20501a	5.57	3.32	0.36	0.14	0.04	0.03	-0.06
	14	SWU13909	TMB0071	20	SWU20035	DPL0319	5.27	3.36	0.53	0.14	0.07	-0.06	-0.01
	5	SWU20913	Gh260	21	SWU16651	SWU16645	5.55	2.05	2.17	-0.11	-0.15	0.02	0.13
	7	NAU1357	SWU10067	22	SWU21533	DPL0562	5.47	2.34	1.24	-0.12	-0.09	-0.03	0.12
	10	Gh320	HAU0635	24	Gh298	SWU13133	6.03	2.76	1.13	-0.13	-0.05	-0.07	0.12
	21	SWU16488	SWU16138	27	SWU11384	ICR11885	5.40	2.76	0.77	-0.13	-0.05	-0.05	0.10
	5	TMB1296	HAU1603	27	CGR6857	ICR11205	5.58	3.54	0.39	0.14	0.07	-0.04	-0.03
*t*2	1	NAU3177	ICR03724	2	PGML0700	SWU12016	9.65	3.89	1.71	-0.35	-0.33	0.15	0.18
	1	NAU2218	SWU11191	9	Gh27	SWU15194	5.68	2.95	0.38	-0.30	-0.15	0.04	0.11
	5	PGML1917	SWU17715	9	PGML2830	CGR6876	7.28	3.17	1.11	0.32	0.18	0.08	-0.26
	4	SWU21617	SWU11855	10	BNL2960	SWU20511	5.09	1.11	1.57	-0.19	-0.09	-0.21	0.31
	5	SWU20917	NAU6240	10	Gh320	HAU0635	5.28	1.94	1.49	-0.25	0.07	-0.29	0.22
	1	SWU10912	DPL0090	14	CIR228	BNL2485	5.32	1.83	1.12	0.24	0.19	0.07	-0.26
	13	DPL0894	SWU10800	14	CIR228	BNL2485	5.14	2.10	1.19	0.26	0.23	0.02	-0.24
	7	PGML1916	SWU10864	20	SWU20246	SWU20501a	6.35	3.29	0.44	0.37	0.16	0.06	-0.22
	15	DPL0182	SWU11691	21	CGR5217	BNL3442a	5.31	2.68	0.46	-0.29	-0.15	-0.01	0.16
	4	SWU18881	NAU2701	23	SWU14807	PGML4185	6.05	3.09	0.94	-0.32	-0.14	-0.10	0.24
	13	SWU22413	CGR5331	23	SWU14807	PGML4185	7.19	3.48	1.01	-0.34	-0.17	-0.08	0.25
	20	SWU20035	DPL0319	23	SWU14807	PGML4185	5.30	2.85	0.54	-0.30	-0.15	-0.01	0.17
	23	PGML4186	NAU3100	24	SWU13758	CGR5423	5.63	3.14	0.39	-0.31	-0.13	0.00	0.14
	17	ICR03391	SWU12838	26	Gh64	SWU17257	5.60	3.00	0.98	-0.31	-0.22	0.20	0.02
	1	NAU6367	MUSS422	26	SWU18672	SWU18681	6.00	2.37	0.99	-0.28	-0.02	-0.22	0.24
	5	PGML4350	SWU17781	27	ICR11883	CGR6356	5.98	2.71	0.92	0.29	0.23	-0.04	-0.19
	8	HAU3177	NAU4064	28	CGR5534	SHIN0219	5.08	2.23	0.87	0.27	0.09	0.15	-0.23
	13	NAU2893	Gh157	28	SWU12343	SWU14060	10.2	4.01	1.65	0.36	0.10	0.22	-0.32
	11	NAU3695	DPL0050b	28	NBRI0014	SWU12107	5.04	2.31	0.78	0.27	0.17	0.04	-0.21
	13	DPL0535	CER0165	29	DC20127	DPL0252	6.92	3.18	0.76	0.32	0.05	0.16	-0.21
	7	NAU1357	SWU10067	29	BNL3261	CGR5111	5.14	1.98	0.91	0.25	0.09	0.15	-0.24
	11	CER0098	CGR5421	30	CER0168	SWU21718	5.60	2.78	0.78	0.30	0.15	0.07	-0.22
*t*3	1	NAU3177	ICR03724	2	PGML0700	SWU12016	5.29	2.85	0.48	-0.39	-0.21	0.04	0.18
	2	PGML0700	SWU12016	8	NAU4064	CGR6508	5.45	3.11	0.41	-0.40	0.13	0.07	-0.20
	7	PGML1916	SWU10864	20	SWU20246	SWU20501a	5.91	3.54	0.38	0.49	0.23	-0.04	-0.19

Stage	Chi	Flanking markers		Chj	Flanking markers		LOD	V(AA)	V(AAE)	AA	AAE1	AAE2	AAE3
	17	SWU14627	CGR5871	21	SWU16651	SWU16645	5.29	1.69	1.68	0.30	-0.16	-0.27	0.42
	4	BNL530	SWU21485	26	SWU17432	SWU17395	5.05	3.07	0.50	0.40	-0.05	-0.17	0.22
	5	TMB1296	HAU1603	27	ICR11205	DPL0847a	6.15	4.41	0.01	0.48	0.00	0.00	0.00
*t*4	3	Gh663	CGR6528	8	HAU1470a	SHIN1341	6.61	3.58	0.71	-0.46	0.17	-0.29	0.11
	10	Gh320	HAU0635	14	ICR12037	CGR5675	5.02	2.93	0.39	-0.41	0.21	-0.14	-0.07
	2	PGML0700	SWU12016	18	CIR099	NAU748	5.18	1.98	1.52	0.34	-0.13	-0.28	0.41
	7	PGML1916	SWU10864	20	SWU20246	SWU20501a	6.06	3.90	0.54	0.53	0.15	0.15	-0.30
	16	SWU20341	DPL0897	24	HAU2504	SWU13736	5.95	1.42	2.97	0.29	-0.16	-0.42	0.57
	1	NAU2218	SWU11191	24	SWU13758	CGR5423	6.64	3.86	0.56	-0.47	0.07	0.18	-0.24
	10	ICR00093	ICR07050	29	BNL3261	CGR5111	6.06	4.16	0.06	-0.49	0.04	0.04	-0.08
BCVF1 population													
*t*1	6	DPL0590	NAU2971	10	HAU0635	PGML4154	5.17	1.89	1.35	0.10	0.11	-0.02	-0.10
	4	JESPR234	BNL530	12	Gh243	DPL0400	5.44	2.39	0.75	0.11	0.00	0.08	-0.08
	6	SWU19656	CGR5124	13	CGR5331	SHIN1462	7.44	2.78	1.24	0.12	0.11	-0.02	-0.09
	1	NAU2697	SWU0320	16	HAU3081	NAU747	5.52	1.99	1.33	0.10	-0.04	0.12	-0.08
	14	NAU4045	ICR03943	16	SWU18366	SWU18579	6.77	2.62	1.13	-0.12	-0.06	-0.05	0.12
	10	HAU0635	PGML4154	20	HAU1314	SWU20035	5.26	2.41	0.81	0.11	0.01	0.08	-0.09
	12	CGR6847	SWU17197	21	CGR5748	PGML2500	5.57	1.77	1.12	0.10	0.02	0.09	-0.10
	1	CGR6784	NAU3393	21	SWU16489	SWU16360	5.43	3.51	0.30	-0.14	-0.02	-0.03	0.06
	12	BNL3261	Gh568	23	HAU0244	Gh327	5.31	2.37	1.07	0.12	0.05	0.07	-0.12
	16	C2 0011B	SWU10211	23	NAU2140	DC40286	5.07	1.00	1.73	0.07	0.11	0.03	-0.13
	16	HAU1129	C2 0011B	24	Gh54	Gh454	5.84	1.87	1.58	-0.10	-0.11	0.00	0.12
	14	HAU2482	NAU4045	25	NAU2968	DPL0377	6.18	2.74	0.72	-0.15	-0.03	-0.08	0.10
	19	PGML4342	NAU2894	26	MGHES31	HAU1571	6.59	1.76	1.89	-0.10	-0.07	-0.07	0.14
	21	SWU16489	SWU16360	31	SWU16780	SWU16735	5.15	1.35	1.78	0.09	0.14	-0.06	-0.09
	31	DPL0057	NAU3109	31	SWU16730	SWU16721	6.24	2.06	1.51	0.11	0.04	0.08	-0.13
	2	CGR6695	SWU11013	32	HAU1000	TMB1931	6.37	2.29	1.31	-0.11	0.00	-0.10	0.11
	31	SWU16735	SWU16755	39	SWU16437	SWU16432	7.17	2.11	1.44	0.11	0.09	0.04	-0.12
*t*2	1	Gh120	Gh398	1	DPL0790	ICR03725	5.77	2.02	0.98	0.28	0.04	0.22	-0.26
	6	HAU2768	HAU0483	9	MUSS139	PGML2830	5.10	2.12	1.15	-0.28	0.05	-0.28	0.23
	6	DPL0847	CGR6749	10	SWU19932	HAU0635	6.23	2.98	1.00	0.33	-0.16	0.27	-0.11
	4	BNL1167	JESPR234	12	ICR03107	HAU3373	5.82	2.11	1.13	0.28	0.07	0.20	-0.28
	4	BNL530	SWU16781	15	NAU3736	SWU11691	5.56	2.03	0.92	0.27	0.05	0.19	-0.25
	18	SWU21718	SWU0738	21	SWU16493	SWU16489	5.24	1.11	1.66	-0.20	-0.12	-0.23	0.35
	1	CGR6784	NAU3393	21	SWU16489	SWU16360	7.12	3.47	0.98	-0.36	-0.06	-0.20	0.26
	5	Gh260	PGML0120	21	SWU16361	SWU16408	5.71	2.23	1.06	0.29	0.04	0.22	-0.26
	16	HAU1129	C2 0011B	24	Gh454	HAU3076	5.62	1.49	1.22	-0.23	-0.27	0.03	0.25
	4	SWU16783	SWU18876	24	HAU3076	SWU13121	5.51	2.41	1.06	0.30	0.02	0.23	-0.25
	17	SWU12838a	HAU1413	25	NAU4964	HAU1355	6.27	2.57	1.02	0.31	-0.11	0.27	-0.17
	21	SWU16361	SWU16408	26	MGHES31	HAU1571	5.02	2.99	0.35	0.33	-0.04	0.15	-0.12
	16	HAU1129	C2_0011B	29	DPL0171	Gh499	5.48	1.45	1.90	0.23	-0.13	0.37	-0.24
	29	Gh111	Gh27	31	DPL0057	NAU3109	5.43	2.11	0.74	0.28	0.10	0.13	-0.23
	23	NAU2140	DC40286	31	CGR6772	HAU0355	6.13	2.50	0.95	0.31	0.09	0.17	-0.26
	31	CGR6772	HAU0355	33	BNL3661	PGML4891	5.06	2.67	0.67	0.31	-0.07	0.22	-0.15
	12	HAU3373	CGR6847	35	NAU2139	TMB1152	7.13	3.35	0.59	0.35	-0.03	0.19	-0.17
	9	MUSS139	PGML2830	36	SWU20658	CGR6154	6.23	2.31	1.03	0.29	0.17	0.10	-0.27
	14	HAU0883	CIR228	37	HAU0423	JESPR154	5.48	2.53	1.02	0.30	-0.05	0.26	-0.21
	31	SWU16780	SWU16735	38	NAU2450	PGML1942	6.70	2.71	1.15	-0.32	-0.07	-0.21	0.28
*t*3	21	CGR6521	Gh450	32	HAU1000	TMB1931	5.79	1.31	2.16	-0.25	0.41	-0.38	-0.03

Stage	Chi	Flanking markers		Chj	Flanking markers		LOD	V(AA)	V(AAE)	AA	AAE1	AAE2	AAE3
*t*4	1	SWU10912	HAU2489	1	DPL0790	ICR03725	5.21	0.88	1.52	0.28	-0.49	0.49	0.00
	14	ICR03105	ICR01124	21	CGR5748	PGML2500	5.97	0.31	3.26	0.17	-0.70	0.58	0.12

See footnotes of additional table S8 for explanations

Table S10 Epistatic effects and environmental interactions detected for boll number per plant in two MPH data using two-locus analysis by inclusive composite interval mapping

Stage	Chi	Flanking markers		Chj	Flanking markers		LOD	V(AA)	V(AAE)	AA	AAE1	AAE2	AAE3
MPH (XZ hybrid)													
*t*1	10	ICR00093	ICR07050	10	Gh320	HAU0635	5.43	0.03	2.20	-0.01	-0.01	-0.13	0.14
*t*2	6	ICR00143	CGR5108	8	HAU3177	NAU4064	5.61	1.55	2.01	-0.20	0.01	-0.28	0.27
	1	CGR6129	DPL0790	14	PGML1368	PGML1568	5.02	1.56	1.43	0.20	0.23	0.01	-0.23
	13	HAU2558	NAU2893	16	PGML1709	SWU10627	5.47	2.28	1.51	-0.25	-0.24	0.00	0.24
	17	SWU12818	CGR5576	19	SWU17882	CAU0104	5.13	1.45	1.66	0.19	-0.06	0.28	-0.22
	4	SWU18881	NAU2701	20	SWU20246	SWU20501a	5.09	1.77	1.25	-0.21	-0.07	-0.18	0.24
	10	Gh320	HAU0635	23	PGML4186	NAU3100	5.78	0.55	3.20	-0.12	-0.35	0.01	0.34
	1	HAU1417	NAU2437	28	SWU12343	SWU14060	5.36	0.43	2.66	0.11	0.31	0.00	-0.32
	4	SWU12672	HAU1332	29	C2 0115	ICR03107	5.36	1.81	1.39	-0.21	-0.22	-0.01	0.24
*t*3	4	ICR01729	SWU16781	13	SWU22413	CGR5331	5.22	1.54	1.90	-0.25	0.02	-0.35	0.33
	14	SWU14224	DPL0565	23	PGML4186	NAU3100	5.62	2.95	1.01	-0.36	-0.17	-0.13	0.30
	6	ICR00143	CGR5108	25	SWU19411	SWU19412	5.30	2.57	1.61	0.33	-0.09	0.36	-0.27
	16	SWU10038	ICR00016	26	SWU18488	SWU18672	5.07	2.30	1.19	0.32	-0.09	0.33	-0.23
*t*4	3	SWU12732	SWU12783	5	CGR5025	NBRI0694	5.70	3.78	0.66	0.45	-0.03	-0.23	0.26
	19	TMB0107	NAU3217	24	SWU13745	Gh273	5.05	2.81	1.03	0.39	-0.20	-0.13	0.34
	5	SWU13378	SWU17846	25	DPL0282	SWU19763	5.32	3.40	0.49	-0.43	0.18	0.04	-0.21
MPH (XZV hybrid)													
*t*1	1	Gh529	SWU17434	1	ICR03725	ICR03724	5.02	0.94	1.78	-0.07	-0.01	-0.12	0.13
	1	HAU2489	DPL0790	9	PGML2830	DC30015	8.08	3.40	1.29	0.14	0.07	0.05	-0.12
	8	HAU0810	TMB2904	18	SWU21718	SWU0738	6.29	1.55	1.76	-0.09	-0.11	-0.01	0.13
	8	HAU0810	TMB2904	21	BNL3171	HAU2937	5.26	2.81	0.35	0.12	0.05	0.00	-0.06
	16	HAU1129	C2 0011B	24	Gh454	HAU3076	6.09	2.69	1.00	-0.12	-0.03	-0.07	0.11
	14	HAU2482	NAU4045	25	NAU2968	DPL0377	6.69	2.56	0.95	-0.15	-0.01	-0.10	0.11
	10	NAU4967	SWU19932	26	MGHES31	HAU1571	5.09	1.72	1.85	0.10	0.14	-0.03	-0.11
	10	NAU3395	CAU0234	28	BNL3545	PGML3983	6.00	2.03	1.85	-0.11	-0.06	-0.08	0.14
	26	MGHES31	HAU1571	28	BNL3545	PGML3983	5.34	2.26	1.36	0.11	0.03	0.09	-0.12
	21	SWU16493	SWU16489	31	SWU16735	SWU16755	5.06	1.60	1.26	0.10	0.12	-0.04	-0.08
	1	Gh398	CGR6129	35	NAU2139	TMB1152	5.51	2.18	1.30	0.11	0.08	0.04	-0.12
	8	HAU0810	TMB2904	37	BNL5602	JESPR251	5.60	3.10	0.63	-0.13	-0.07	-0.01	0.08
*t*2	1	CGR6784	NAU3393	4	JESPR234	BNL530	5.53	2.13	1.80	-0.28	0.05	-0.33	0.28
	1	SWU14514	Gh120	6	CGR6749	NAU3186	5.03	1.29	2.26	-0.21	0.13	-0.39	0.27
	2	SWU11889	JESPR304	16	HAU1129	C2 0011B	5.61	2.01	1.46	-0.27	-0.30	0.06	0.25

Stage	Chi	Flanking markers		Chj	Flanking markers		LOD	V(AA)	V(AAE)	AA	AAE1	AAE2	AAE3
	14	ICR03105	ICR01124	21	SWU16489	SWU16360	9.36	3.38	2.95	0.45	-0.16	0.57	-0.42
	8	HAU0810	TMB2904	25	HAU3012	SWU19676	5.15	2.78	0.55	0.32	0.02	0.16	-0.18
	17	HAU1413	CGR5576	29	DPL0171	Gh499	5.12	0.84	1.85	-0.17	-0.15	-0.22	0.36
	25	NAU2968	DPL0377	31	DPL0057	NAU3109	5.58	1.66	1.83	-0.24	-0.09	-0.25	0.35
	23	HAU0244	Gh327	31	CGR6772	HAU0355	5.77	2.02	1.84	0.29	-0.27	0.39	-0.11
*t*3	6	MUSB1144	BNL3650	8	DC20094	Gh197	5.58	3.12	0.51	0.49	-0.28	0.10	0.18
	1	NAU0748	NAU2697	12	CGR6847	SWU17197	5.04	2.11	0.97	0.40	-0.38	0.23	0.15
	1	Gh529	SWU17434	14	HAU2482	NAU4045	7.60	3.77	0.90	0.64	-0.43	0.33	0.10
	6	DC40417	MUSB116	14	HAU2482	NAU4045	5.47	2.90	0.51	-0.58	0.30	-0.02	-0.28
	13	SWU13032	HAU2850	15	NAU3736	SWU11691	5.80	2.46	0.61	0.43	-0.29	0.23	0.06
	14	HAU0883	CIR228	17	HAU1413	CGR5576	5.34	2.66	0.78	-0.45	0.24	-0.33	0.09
	1	PGML2498	SWU14490	21	CGR5748	PGML2500	5.10	3.04	0.46	-0.48	0.22	0.02	-0.23
	8	DC20094	Gh197	21	SWU16489	SWU16360	5.11	3.38	0.32	0.52	-0.09	-0.12	0.21
	17	HAU1413	CGR5576	21	BNL3171	HAU2937	5.68	2.02	1.22	0.39	-0.42	0.11	0.32
	1	CGR6129	ICR03295	23	HAU0244	Gh327	5.07	2.70	0.70	0.49	-0.19	-0.15	0.34
	6	BNL3650	TMB2940	23	NAU3588	NAU5373a	5.44	3.05	0.41	-0.48	0.25	-0.14	-0.11
	24	Gh54	Gh454	24	CGR6079	SWU13100	5.37	2.77	0.30	-0.52	0.35	-0.17	-0.19
	6	BNL3650	TMB2940	25	DPL0377	SWU19413	5.88	3.39	0.51	0.51	-0.26	0.07	0.20
	12	HAU3373	CGR6847	26	HAU1571	CGR6477	5.10	2.53	0.68	-0.44	0.30	-0.03	-0.27
	21	BNL3171	HAU2937	31	HAU0355	SWU16777	5.67	1.71	1.50	-0.36	0.48	-0.22	-0.26
	16	CGR5506	CGR5925	31	SWU16730	SWU16721	6.25	3.33	0.72	0.51	-0.33	0.18	0.15
	6	MUSB1144	BNL3650	34	JESPR297	ICR00647	5.03	2.98	0.27	0.48	-0.20	0.05	0.15
	16	C2 0011B	SWU10211	37	DPL0777	HAU0423	6.86	3.05	0.91	-0.48	0.37	-0.20	-0.17
	10	HAU0635	PGML4154	38	NAU2450	PGML1942	5.86	2.19	1.08	-0.41	0.39	-0.10	-0.29
	24	PGML1207	Gh54	39	HAU2022	BNL0827	5.00	2.78	0.50	0.46	-0.21	-0.06	0.27
	13	CER0165	SWU13032	39	NAU5480	DPL0270	7.19	3.63	1.90	-0.52	0.25	0.28	-0.53
*t*4	1	CGR6129	ICR03295	2	DPL0217	CGR6695	5.14	2.26	0.45	-0.44	0.27	-0.17	-0.10
	5	Gh260	PGML0120	5	Gh260	PGML0120	5.22	2.70	0.96	-0.51	0.12	0.30	-0.42
	2	TMB1268	SWU11976	6	DC40417	MUSB1164	6.44	3.57	0.57	-0.55	0.26	0.02	-0.28
	6	SWU19656	CGR5124	6	CGR5124	SWU19249	5.23	0.81	0.00	-0.73	0.62	-0.33	-0.29
	6	MUSB1144	BNL3650	8	DC20094	Gh197	5.13	2.49	0.85	0.46	-0.34	0.01	0.33
	1	Gh398	CGR6129	8	HAU0810	TMB2904	5.28	1.80	1.23	0.39	-0.43	0.08	0.35
	9	DC30015	SWU15413	12	ICR03107	HAU3373	5.16	1.76	0.79	-0.39	0.30	-0.34	0.04
	6	CGR6749	NAU3186	12	HAU3373	CGR6847	6.32	2.55	1.90	-0.46	0.29	0.28	-0.57
	8	DC20094	Gh197	14	ICR03943	ICR12281	5.79	2.74	0.52	0.51	-0.30	0.06	0.24
	14	HAU0883	CIR228	17	HAU1413	CGR5576	5.51	2.77	0.57	-0.49	0.30	-0.20	-0.10
	13	CER0165	SWU13032	18	SWU0738	ICR02849	5.83	2.95	0.52	-0.55	0.33	-0.14	-0.19
	1	NAU0748	NAU2697	21	CGR5748	PGML2500	5.70	2.23	1.07	-0.44	0.43	-0.23	-0.20
	15	NAU3736	SWU11691	21	CGR6521	Gh450	5.63	2.89	0.49	0.50	-0.05	-0.23	0.27
	1	SWU11632	SWU21958	23	Gh327	ICR06429	5.24	1.28	1.67	-0.36	0.46	0.09	-0.55
	24	Gh54	Gh454	24	CGR6079	SWU13100	6.35	2.81	0.85	-0.54	0.47	-0.05	-0.41
	6	BNL3650	TMB2940	25	NAU2968	DPL0377	5.25	3.00	0.32	0.50	-0.16	-0.06	0.22
	5	Gh260	PGML0120	25	SWU19434	SWU19412	7.36	3.38	1.01	0.56	-0.41	0.25	0.17
	12	HAU3373	CGR6847	26	CGR6477	PGML2562	5.82	2.53	1.01	-0.47	0.35	0.03	-0.38
	13	NAU3398	CGR5331	31	CGR6772	HAU0355	5.82	2.96	0.56	-0.50	0.24	-0.30	0.05
	9	CGR6876	BNL1317	31	SWU16730	SWU16721	5.09	2.83	0.28	-0.49	0.22	-0.12	-0.10
	6	MUSB1144	BNL3650	34	JESPR297	ICR00647	6.78	2.48	1.37	0.47	-0.48	0.15	0.33
	25	SWU19676	NAU2968	36	CER0167	SWU20658	5.24	2.08	1.31	-0.42	0.35	0.11	-0.46
	24	PGML1207	Gh54	39	HAU2022	BNL0827	5.73	2.64	0.99	0.48	-0.25	-0.17	0.42

Stage	Chi	Flanking markers		Chj	Flanking markers		LOD	V(AA)	V(AAE)	AA	AAE1	AAE2	AAE3
	13	CER0165	SWU13032	39	NAU5480	DPL0270	6.99	2.85	2.71	-0.49	0.25	0.42	-0.67

See footnotes of additional table S8 for explanations

Table S11 Correlation between yield and yield components in RIL(V) and BC(V) data sets

Population	Trait	Env.	BNP(*t*1)	BNP(*t*2)	BNP(*t*3)	BNP(*t*4)	SY	LY	BW
RIL	BNP(*t*2)	E1	0.65**						
		E2	0.70**						
		E3	0.74**						
	BNP(*t*3)	E1	0.47**	0.88**					
		E2	0.52**	0.81**					
		E3	0.59**	0.75**					
	BNP(*t*4)	E1	0.32**	0.81**	0.92**				
		E2	0.41**	0.72**	0.90**				
		E3	0.43**	0.54**	0.77**				
	SY	E1	0.33**	0.60**	0.63**	0.63**			
		E2	0.38**	0.54**	0.63**	0.70**			
		E3	0.49**	0.69**	0.76**	0.60**			
	LY	E1	0.37**	0.61**	0.66**	0.65**	0.96**		
		E2	0.41**	0.57**	0.67**	0.72**	0.96**		
		E3	0.50**	0.70**	0.81**	0.66**	0.97**		
	BW	E1	0.27**	0.07	-0.03	-0.1	0.38**	0.35**	
		E2	0.33**	0.18*	0	-0.03	0.48**	0.39**	
		E3	0.09	0.23**	0.07	-0.14	0.40**	0.31**	
	LP	E1	0.12	0.09	0.11	0.08	-0.14	0.13	-0.12
		E2	0.12	0.11	0.17*	0.11	-0.13	0.16*	-0.33**
		E3	0.19**	0.28**	0.44**	0.43**	0.23**	0.46**	-0.21**
RILV	BNP(*t*2)	E1	0.79**						
		E2	0.83**						
		E3	0.67**						
	BNP(*t*3)	E1	0.71**	0.92**					
		E2	0.78**	0.91**					
		E3	0.32**	0.80**					
	BNP(*t*4)	E1	0.53**	0.77**	0.88**				
		E2	0.71**	0.87**	0.96**				
		E3	0.28**	0.78**	0.95**				
	SY	E1	0.21**	0.39**	0.50**	0.59**			
		E2	0.66**	0.80**	0.86**	0.86**			
		E3	0.30**	0.80**	0.95**	0.95**			

Population	Trait	Env.	BNP($t1$)	BNP($t2$)	BNP($t3$)	BNP($t4$)	SY	LY	BW
	LY	E1	0.27**	0.47**	0.56**	0.62**	0.96**		
		E2	0.66**	0.79**	0.83**	0.83**	0.96**		
		E3	0.30**	0.80**	0.95**	0.95**	1.00**		
	BW	E1	0.16*	0.11	0.09	-0.01	0.37**	0.36**	
		E2	0.24**	0.19**	0.17*	0.15	0.42**	0.41**	
		E3	-0.05	-0.02	-0.04	-0.04	0.06	0.06	
	LP	E1	0.30**	0.40**	0.37**	0.33**	0.20**	0.45**	0.08
		E2	0.07	0.06	0	-0.01	-0.01	0.25**	0.04
		E3	0.09	0.18*	0.23**	0.25**	0.21**	0.29**	-0.08
BCF_1	BNP($t2$)	E1	0.54**						
		E2	0.60**						
		E3	0.69**						
	BNP($t3$)	E1	0.48**	0.81**					
		E2	0.38**	0.56**					
		E3	0.40**	0.55**					
	BNP($t4$)	E1	0.49**	0.79**	0.88**				
		E2	0.37**	0.50**	0.80**				
		E3	0.32**	0.52**	0.55**				
	SY	E1	0.31**	0.44**	0.48**	0.49**			
		E2	0.40**	0.43**	0.60**	0.65**			
		E3	0.38**	0.64**	0.66**	0.60**			
	LY	E1	0.30**	0.44**	0.50**	0.52**	0.94**		
		E2	0.44**	0.43**	0.59**	0.66**	0.95**		
		E3	0.39**	0.66**	0.67**	0.59**	0.98**		
	BW	E1	0.08	-0.06	-0.05	-0.04	0.20**	0.22**	
		E2	0.32**	0.21**	0.1	0.03	0.37**	0.37**	
		E3	0.1	0.15*	0.05	0.02	0.26**	0.20**	
	LP	E1	0.03	0.07	0.15*	0.18*	0.04	0.36**	0.11
		E2	0.19*	0.11	0.1	0.18*	0.03	0.34**	0.06
		E3	0.16*	0.29**	0.23**	0.1	0.16*	0.35**	-0.20**
$BCVF_1$	BNP($t2$)	E1	0.57**						
		E2	0.60**						
		E3	0.68**						
	BNP($t3$)	E1	0.52**	0.76**					
		E2	0.48**	0.61**					
		E3	0.40**	0.66**					
	BNP($t4$)	E1	0.42**	0.56**	0.80**				
		E2	0.43**	0.58**	0.86**				
		E3	0.29**	0.56**	0.73**				
	SY	E1	0.11	0.13	0.22**	0.27**			
		E2	0.38**	0.50**	0.74**	0.78**			
		E3	0.32**	0.59**	0.69**	0.72**			
	LY	E1	0.13	0.17*	0.24**	0.27**	0.98**		

Population	Trait	Env.	BNP(*t*1)	BNP(*t*2)	BNP(*t*3)	BNP(*t*4)	SY	LY	BW
		E2	0.37**	0.52**	0.73**	0.76**	0.95**		
		E3	0.30**	0.57**	0.67**	0.70**	0.98**		
	BW	E1	0.17*	0.06	0.01	0.01	0.14	0.16*	
		E2	0.19*	0.05	0.03	-0.06	0.19*	0.17*	
		E3	0.12	0.21**	0.04	-0.02	0.20**	0.19*	
	LP	E1	0.17*	0.25**	0.16*	0.07	0.15	0.34**	0.16*
		E2	0.09	0.23**	0.20**	0.21**	0.17*	0.46**	-0.01
		E3	-0.01	0.09	0.08	0.12	0.16*	0.34**	-0.01

*,** Significant at probability of 0.05 and 0.01 respectively. SY, seed cotton yield; LY, lint yield; BNP, bolls per plant; BW, boll weight; LP, lint percent

Table S12 Number of QTLs detected in MPH data sets, and number of QTLs overlapping among the three data sets

Stage	XZ hybrid			XZV hybrid		
	MPH	MPH/(BC or RIL)	Overlapping ratio (%)	MPH	MPH/(BCV or RILV)	Overlapping ratio (%)
*t*1	5	2	40.0	3	2	66.7
*t*2	3	0	0.0	5	1	20.0
*t*3	2	0	0.0	5	1	20.0
*t*4	2	2	100.0	1	1	100.0

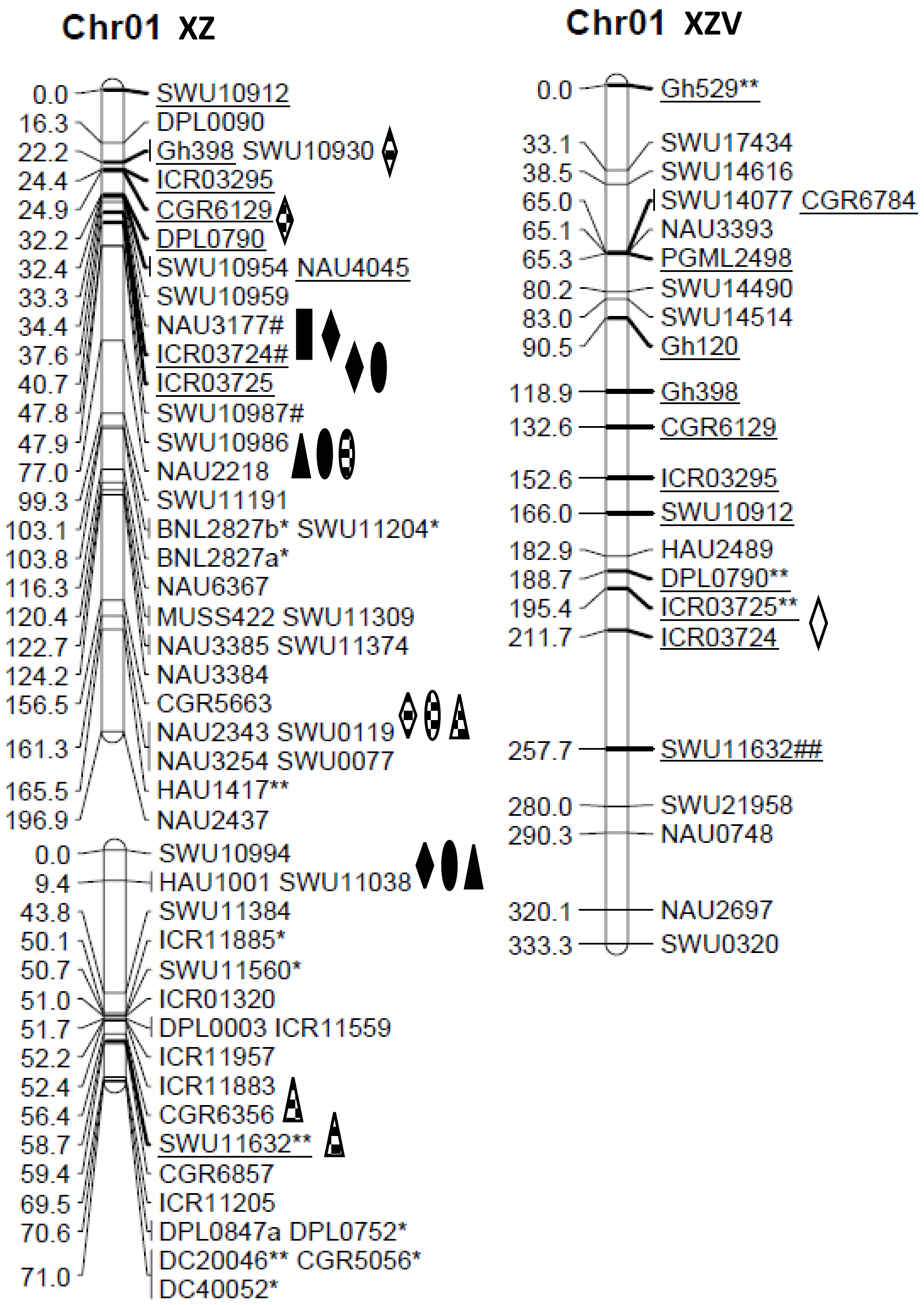

Chr01 XZ
0.0 SWU10912
16.3 DPL0090
22.2 Gh398 SWU10930
24.4 ICR03295
24.9 CGR6129
32.2 DPL0790
32.4 SWU10954 NAU4045
33.3 SWU10959
34.4 NAU3177#
37.6 ICR03724#
40.7 ICR03725
47.8 SWU10987#
47.9 SWU10986
77.0 NAU2218
99.3 SWU11191
103.1 BNL2827b* SWU11204*
103.8 BNL2827a*
116.3 NAU6367
120.4 MUSS422 SWU11309
122.7 NAU3385 SWU11374
124.2 NAU3384
156.5 CGR5663
161.3 NAU2343 SWU0119
NAU3254 SWU0077
165.5 HAU1417**
196.9 NAU2437
0.0 SWU10994
9.4 HAU1001 SWU11038
43.8 SWU11384
50.1 ICR11885*
50.7 SWU11560*
51.0 ICR01320
51.7 DPL0003 ICR11559
52.2 ICR11957
52.4 ICR11883
56.4 CGR6356
58.7 SWU11632**
59.4 CGR6857
69.5 ICR11205
70.6 DPL0847a DPL0752*
71.0 DC20046** CGR5056*
DC40052*
Chr01 XZV
0.0 Gh529**
33.1 SWU17434
38.5 SWU14616
65.0 SWU14077 CGR6784
65.1 NAU3393
65.3 PGML2498
80.2 SWU14490
83.0 SWU14514
90.5 Gh120
118.9 Gh398
132.6 CGR6129
152.6 ICR03295
166.0 SWU10912
182.9 HAU2489
188.7 DPL0790**
195.4 ICR03725**
211.7 ICR03724
257.7 SWU11632##
280.0 SWU21958
290.3 NAU0748
320.1 NAU2697
333.3 SWU0320

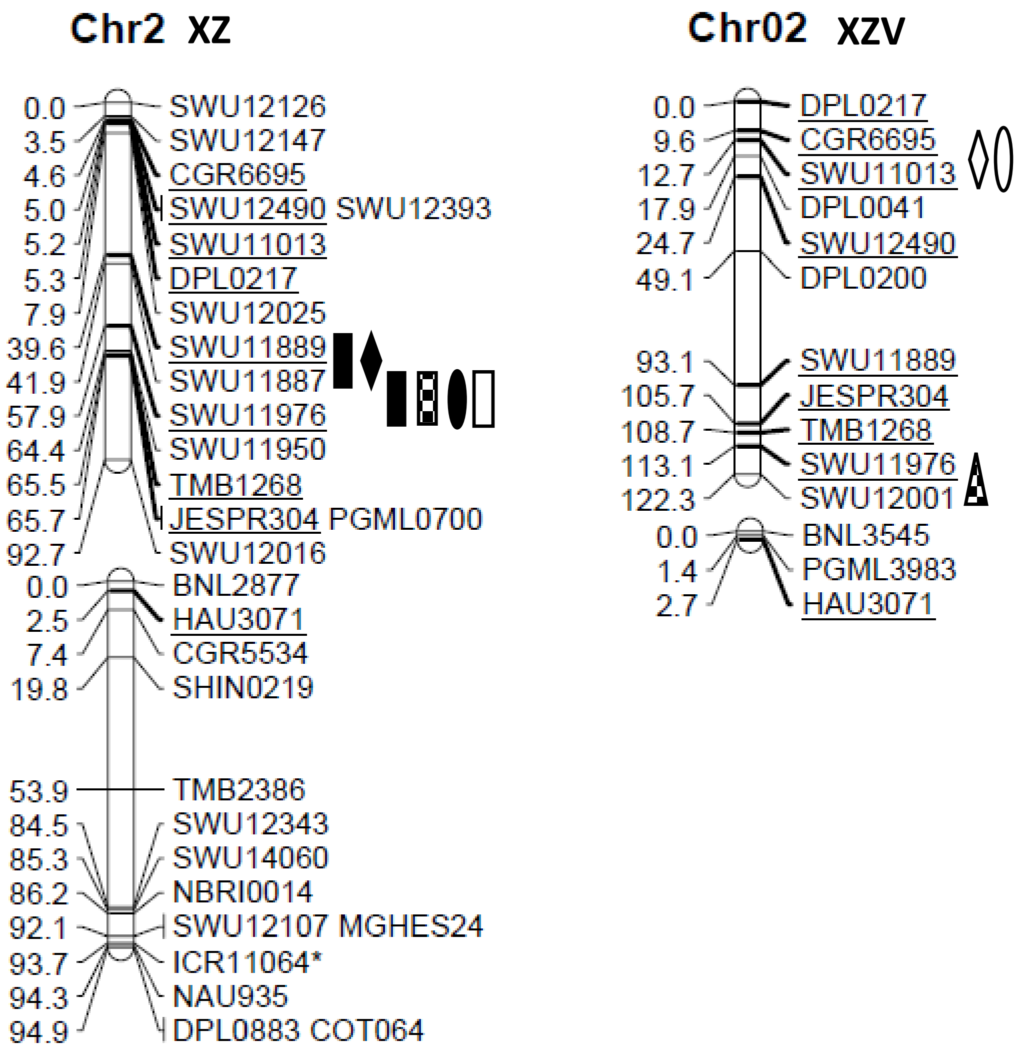
Chr2 XZ
0.0 SWU12126
3.5 SWU12147
4.6 CGR6695
5.0 SWU12490 SWU12393
5.2 SWU11013
5.3 DPL0217
7.9 SWU12025
39.6 SWU11889
41.9 SWU11887
57.9 SWU11976
64.4 SWU11950
65.5 TMB1268
65.7 JESPR304 PGML0700
92.7 SWU12016
0.0 BNL2877
2.5 HAU3071
7.4 CGR5534
19.8 SHIN0219
53.9 TMB2386
84.5 SWU12343
85.3 SWU14060
86.2 NBRI0014
92.1 SWU12107 MGHES24
93.7 ICR11064*
94.3 NAU935
94.9 DPL0883 COT064
Chr02 XZV
0.0 DPL0217
9.6 CGR6695
12.7 SWU11013
17.9 DPL0041
24.7 SWU12490
49.1 DPL0200
93.1 SWU11889
105.7 JESPR304
108.7 TMB1268
113.1 SWU11976
122.3 SWU12001
0.0 BNL3545
1.4 PGML3983
2.7 HAU3071

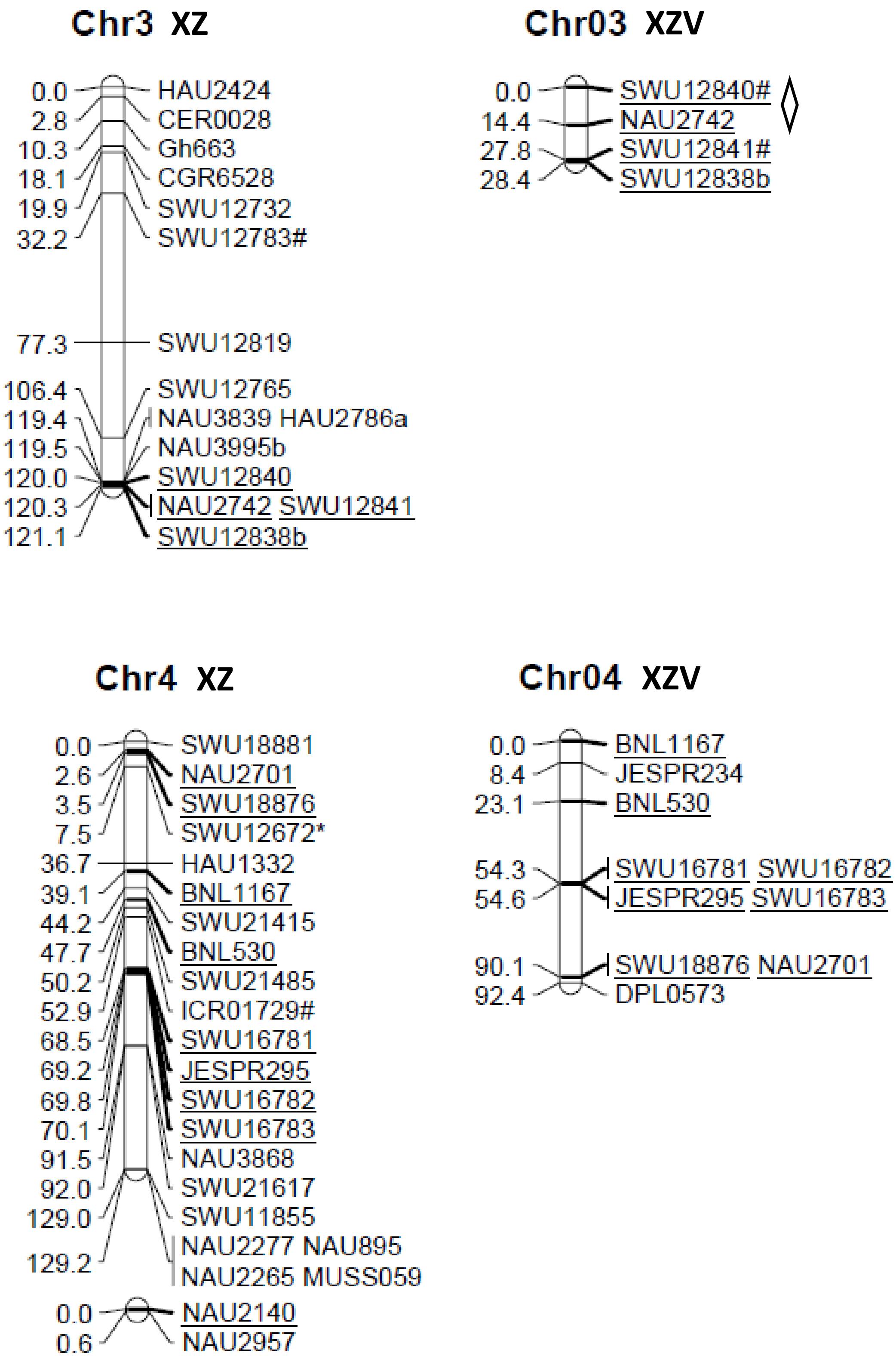

Chr3 XZ
0.0 HAU2424
2.8 CER0028
10.3 Gh663
18.1 CGR6528
19.9 SWU12732
32.2 SWU12783#
77.3 SWU12819
106.4 SWU12765
119.4 NAU3839 HAU2786a
119.5 NAU3995b
120.0 SWU12840
120.3 NAU2742 SWU12841
121.1 SWU12838b
Chr03 XZV
0.0 SWU12840#
14.4 NAU2742
27.8 SWU12841#
28.4 SWU12838b
Chr4 XZ
0.0 SWU18881
2.6 NAU2701
3.5 SWU18876
7.5 SWU12672*
36.7 HAU1332
39.1 BNL1167
44.2 SWU21415
47.7 BNL530
50.2 SWU21485
52.9 ICR01729#
68.5 SWU16781
69.2 JESPR295
69.8 SWU16782
70.1 SWU16783
91.5 NAU3868
92.0 SWU21617
129.0 SWU11855
129.2 NAU2277 NAU895
NAU2265 MUSS059
0.0 NAU2140
0.6 NAU2957
Chr04 XZV
0.0 BNL1167
8.4 JESPR234
23.1 BNL530
54.3 SWU16781 SWU16782
54.6 JESPR295 SWU16783
90.1 SWU18876 NAU2701
92.4 DPL0573

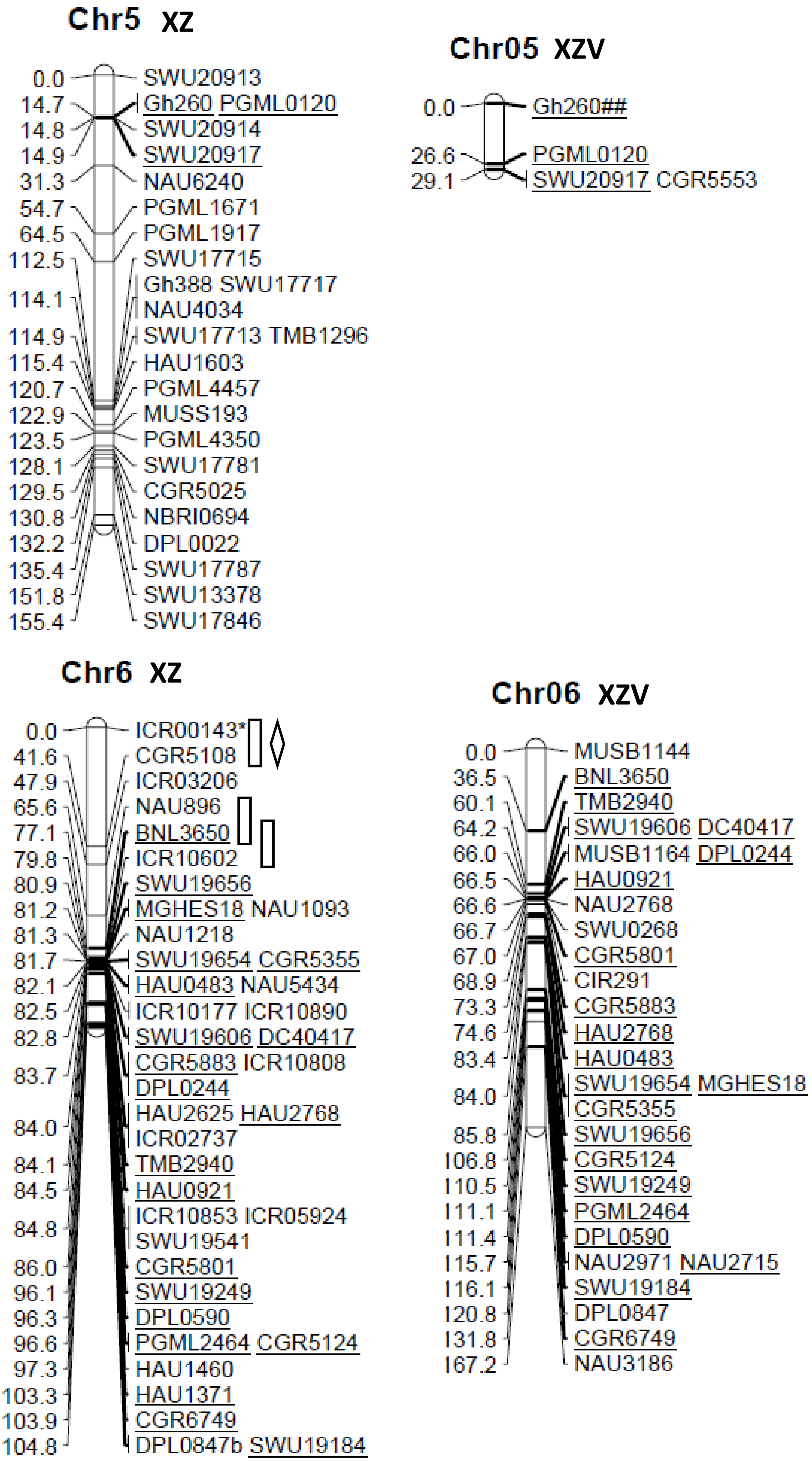
Chr5 XZ
0.0 SWU20913
14.7 Gh260 PGML0120
14.8 SWU20914
14.9 SWU20917
31.3 NAU6240
54.7 PGML1671
64.5 PGML1917
112.5 SWU17715
114.1 Gh388 SWU17717
NAU4034
114.9 SWU17713 TMB1296
115.4 HAU1603
120.7 PGML4457
122.9 MUSS193
123.5 PGML4350
128.1 SWU17781
129.5 CGR5025
130.8 NBRI0694
132.2 DPL0022
135.4 SWU17787
151.8 SWU13378
155.4 SWU17846
Chr05 XZV
0.0 Gh260##
26.6 PGML0120
29.1 SWU20917 CGR5553
Chr6 XZ
0.0 ICR00143*
41.6 CGR5108
47.9 ICR03206
65.6 NAU896
77.1 BNL3650
79.8 ICR10602
80.9 SWU19656
81.2 MGHES18 NAU1093
81.3 NAU1218
81.7 SWU19654 CGR5355
82.1 HAU0483 NAU5434
82.5 ICR10177 ICR10890
82.8 SWU19606 DC40417
83.7 CGR5883 ICR10808
DPL0244
84.0 HAU2625 HAU2768
ICR02737
84.1 TMB2940
84.5 HAU0921
84.8 ICR10853 ICR05924
SWU19541
86.0 CGR5801
96.1 SWU19249
96.3 DPL0590
96.6 PGML2464 CGR5124
97.3 HAU1460
103.3 HAU1371
103.9 CGR6749
104.8 DPL0847b SWU19184
Chr06 XZV
0.0 MUSB1144
36.5 BNL3650
60.1 TMB2940
64.2 SWU19606 DC40417
66.0 MUSB1164 DPL0244
66.5 HAU0921
66.6 NAU2768
66.7 SWU0268
67.0 CGR5801
68.9 CIR291
73.3 CGR5883
74.6 HAU2768
83.4 HAU0483
84.0 SWU19654 MGHES18
CGR5355
85.8 SWU19656
106.8 CGR5124
110.5 SWU19249
111.1 PGML2464
111.4 DPL0590
115.7 NAU2971 NAU2715
116.1 SWU19184
120.8 DPL0847
131.8 CGR6749
167.2 NAU3186

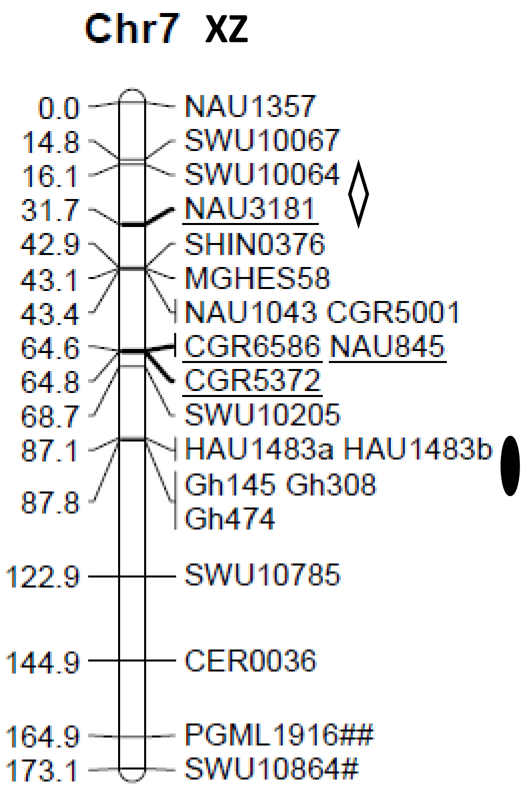

Chr7 XZ
0.0 NAU1357
14.8 SWU10067
16.1 SWU10064
31.7 NAU3181
42.9 SHIN0376
43.1 MGHES58
43.4 NAU1043 CGR5001
64.6 CGR6586 NAU845
64.8 CGR5372
68.7 SWU10205
87.1 HAU1483a HAU1483b
87.8 Gh145 Gh308
Gh474
122.9 SWU10785
144.9 CER0036
164.9 PGML1916##
173.1 SWU10864#

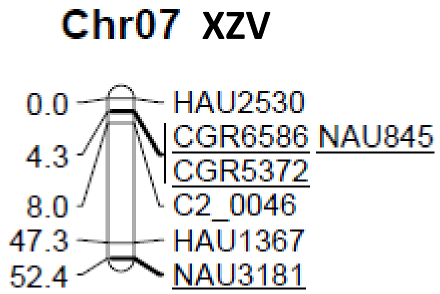

Chr07 XZV
0.0 HAU2530
4.3 CGR6586 NAU845
CGR5372
8.0 C2_0046
47.3 HAU1367
52.4 NAU3181

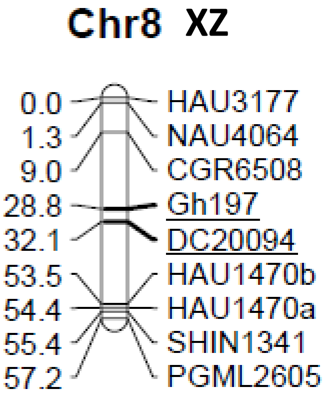

Chr8 XZ
0.0 HAU3177
1.3 NAU4064
9.0 CGR6508
28.8 Gh197
32.1 DC20094
53.5 HAU1470b
54.4 HAU1470a
55.4 SHIN1341
57.2 PGML2605

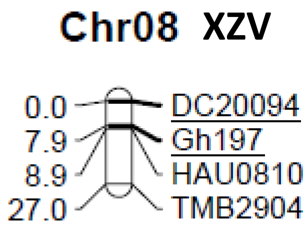

Chr08 XZV
0.0 DC20094
7.9 Gh197
8.9 HAU0810
27.0 TMB2904

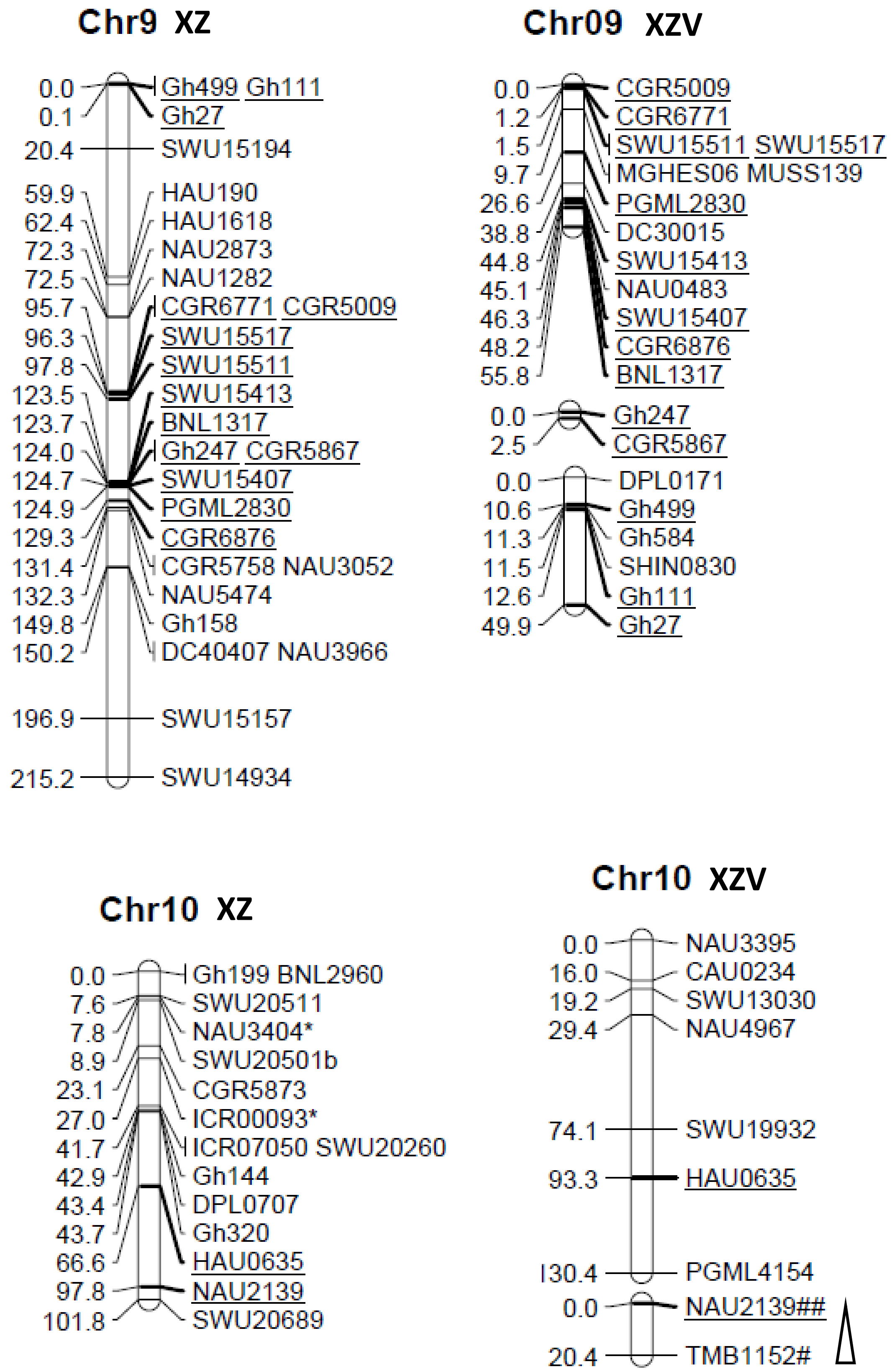

Chr9 XZ
0.0 Gh499 Gh111
0.1 Gh27
20.4 SWU15194
59.9 HAU190
62.4 HAU1618
72.3 NAU2873
72.5 NAU1282
95.7 CGR6771 CGR5009
96.3 SWU15517
97.8 SWU15511
123.5 SWU15413
123.7 BNL1317
124.0 Gh247 CGR5867
124.7 SWU15407
124.9 PGML2830
129.3 CGR6876
131.4 CGR5758 NAU3052
132.3 NAU5474
149.8 Gh158
150.2 DC40407 NAU3966
196.9 SWU15157
215.2 SWU14934
Chr09 XZV
0.0 CGR5009
1.2 CGR6771
1.5 SWU15511 SWU15517
9.7 MGHES06 MUSS139
26.6 PGML2830
38.8 DC30015
44.8 SWU15413
45.1 NAU0483
46.3 SWU15407
48.2 CGR6876
55.8 BNL1317
0.0 Gh247
2.5 CGR5867
0.0 DPL0171
10.6 Gh499
11.3 Gh584
11.5 SHIN0830
12.6 Gh111
49.9 Gh27
Chr10 XZ
0.0 Gh199 BNL2960
7.6 SWU20511
7.8 NAU3404*
8.9 SWU20501b
23.1 CGR5873
27.0 ICR00093*
41.7 ICR07050 SWU20260
42.9 Gh144
43.4 DPL0707
43.7 Gh320
66.6 HAU0635
97.8 NAU2139
101.8 SWU20689
Chr10 XZV
0.0 NAU3395
16.0 CAU0234
19.2 SWU13030
29.4 NAU4967
74.1 SWU19932
93.3 HAU0635
130.4 PGML4154
0.0 NAU2139##
20.4 TMB1152#

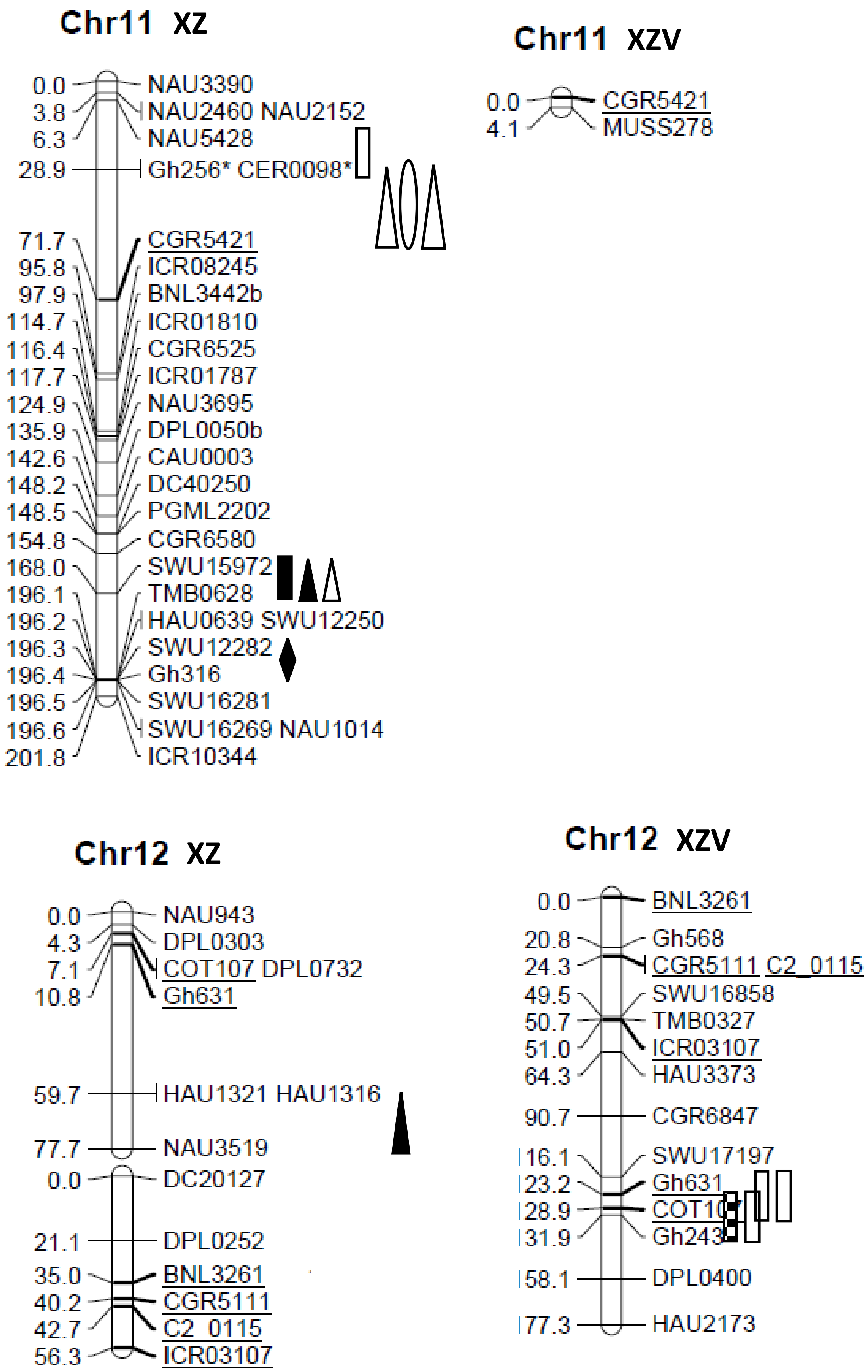
Chr11 XZ
0.0 NAU3390
3.8 NAU2460 NAU2152
6.3 NAU5428
28.9 Gh256* CER0098*
71.7 CGR5421
95.8 ICR08245
97.9 BNL3442b
114.7 ICR01810
116.4 CGR6525
117.7 ICR01787
124.9 NAU3695
135.9 DPL0050b
142.6 CAU0003
148.2 DC40250
148.5 PGML2202
154.8 CGR6580
168.0 SWU15972
196.1 TMB0628
196.2 HAU0639 SWU12250
196.3 SWU12282
196.4 Gh316
196.5 SWU16281
196.6 SWU16269 NAU1014
201.8 ICR10344
Chr11 XZV
0.0 CGR5421
4.1 MUSS278
Chr12 XZ
0.0 NAU943
4.3 DPL0303
7.1 COT107 DPL0732
10.8 Gh631
59.7 HAU1321 HAU1316
77.7 NAU3519
0.0 DC20127
21.1 DPL0252
35.0 BNL3261
40.2 CGR5111
42.7 C2_0115
56.3 ICR03107
Chr12 XZV
0.0 BNL3261
20.8 Gh568
24.3 CGR5111 C2_0115
49.5 SWU16858
50.7 TMB0327
51.0 ICR03107
64.3 HAU3373
90.7 CGR6847
116.1 SWU17197
123.2 Gh631
128.9 COT1
131.9 Gh243
158.1 DPL0400
177.3 HAU2173

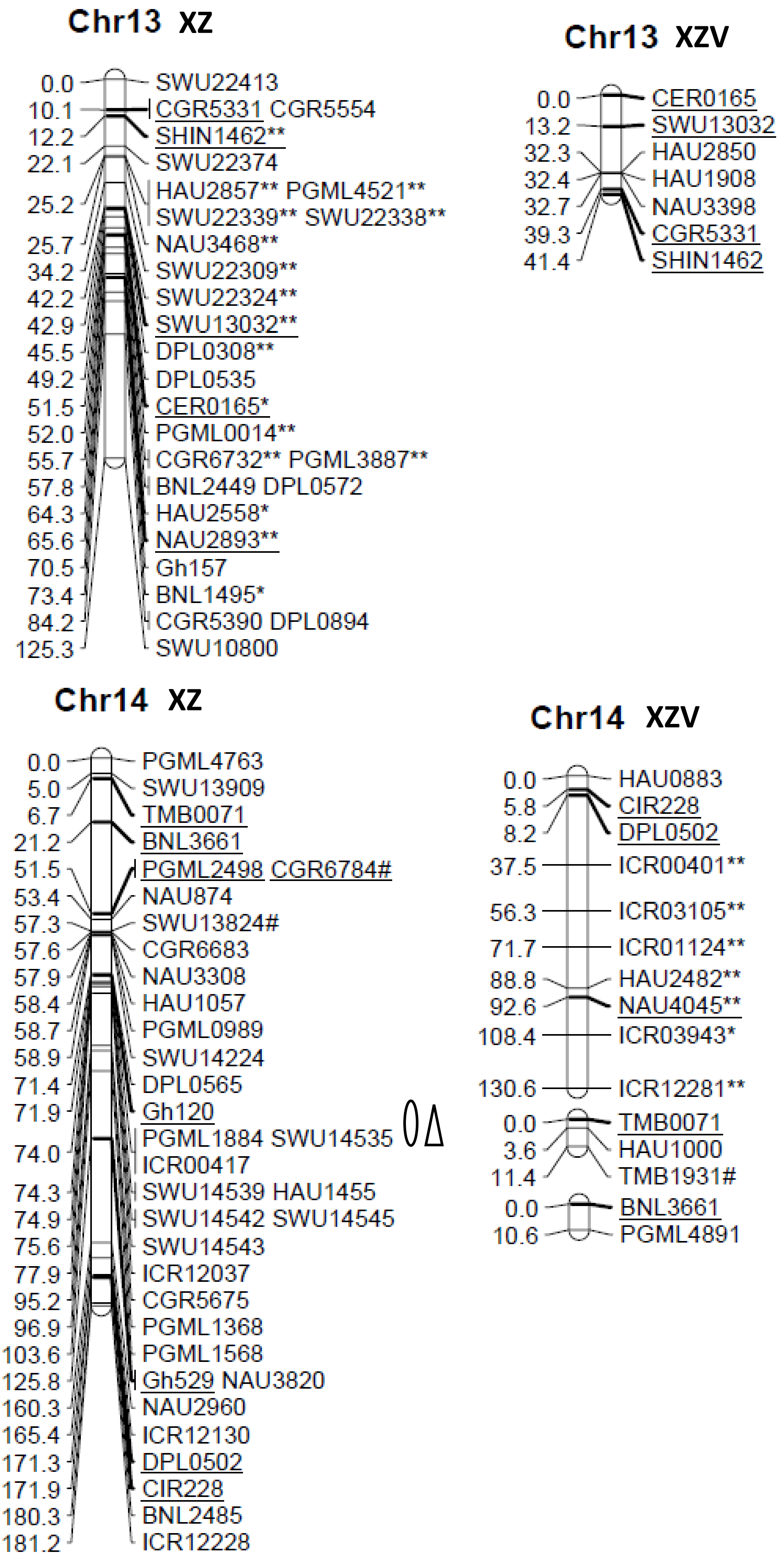

Chr13 XZ
0.0 SWU22413
10.1 CGR5331 CGR5554
12.2 SHIN1462**
22.1 SWU22374
25.2 HAU2857** PGML4521** SWU22339** SWU22338**
25.7 NAU3468**
34.2 SWU22309**
42.2 SWU22324**
42.9 SWU13032**
45.5 DPL0308**
49.2 DPL0535
51.5 CER0165*
52.0 PGML0014**
55.7 CGR6732** PGML3887**
57.8 BNL2449 DPL0572
64.3 HAU2558*
65.6 NAU2893**
70.5 Gh157
73.4 BNL1495*
84.2 CGR5390 DPL0894
125.3 SWU10800
Chr13 XZV
0.0 CER0165
13.2 SWU13032
32.3 HAU2850
32.4 HAU1908
32.7 NAU3398
39.3 CGR5331
41.4 SHIN1462
Chr14 XZ
0.0 PGML4763
5.0 SWU13909
6.7 TMB0071
21.2 BNL3661
51.5 PGML2498 CGR6784#
53.4 NAU874
57.3 SWU13824#
57.6 CGR6683
57.9 NAU3308
58.4 HAU1057
58.7 PGML0989
58.9 SWU14224
71.4 DPL0565
71.9 Gh120
74.0 PGML1884 SWU14535 ICR00417
74.3 SWU14539 HAU1455
74.9 SWU14542 SWU14545
75.6 SWU14543
77.9 ICR12037
95.2 CGR5675
96.9 PGML1368
103.6 PGML1568
125.8 Gh529 NAU3820
160.3 NAU2960
165.4 ICR12130
171.3 DPL0502
171.9 CIR228
180.3 BNL2485
181.2 ICR12228
Chr14 XZV
0.0 HAU0883
5.8 CIR228
8.2 DPL0502
37.5 ICR00401**
56.3 ICR03105**
71.7 ICR01124**
88.8 HAU2482**
92.6 NAU4045**
108.4 ICR03943*
130.6 ICR12281**
0.0 TMB0071
3.6 HAU1000
11.4 TMB1931#
0.0 BNL3661
10.6 PGML4891

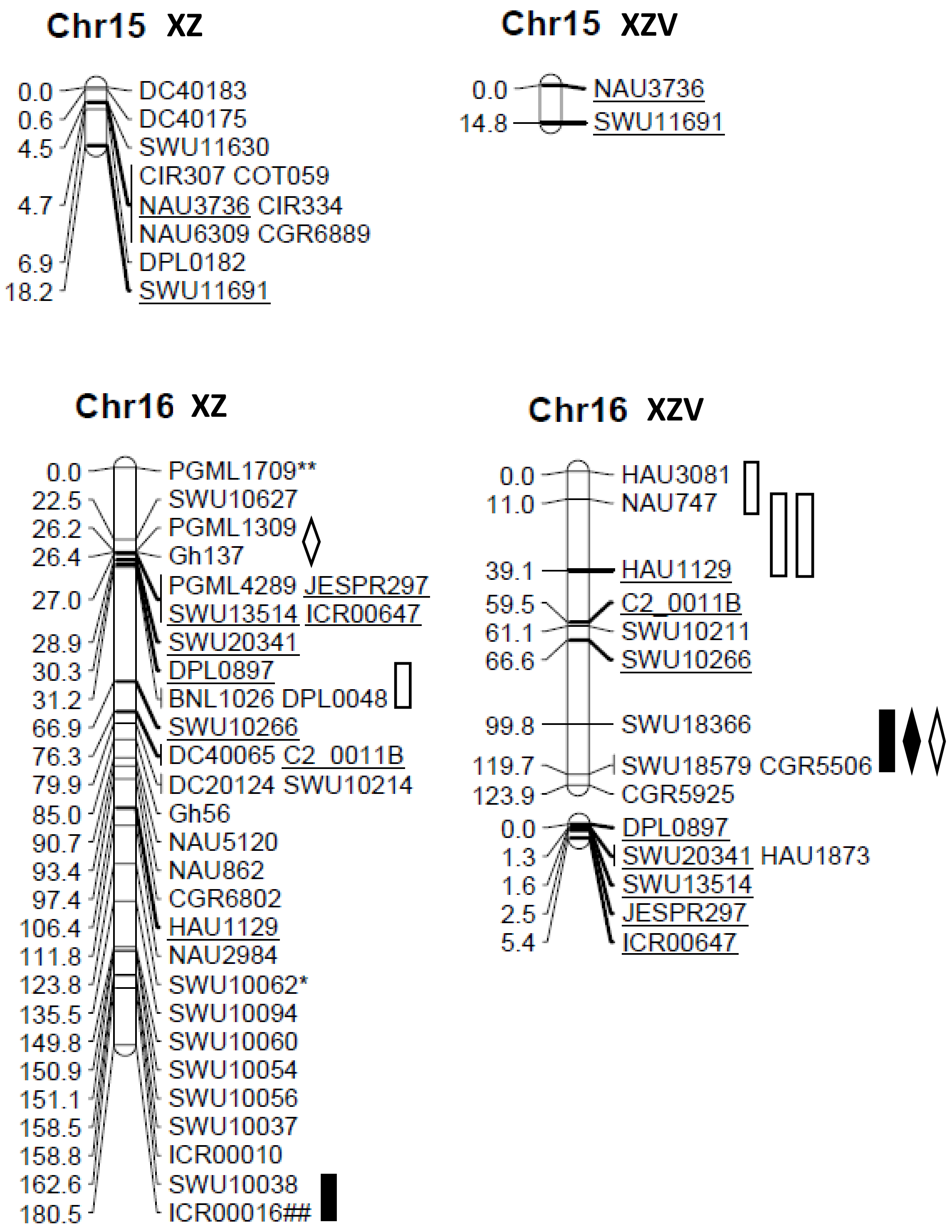

Chr15 XZ
0.0 DC40183
0.6 DC40175
4.5 SWU11630
CIR307 COT059
4.7 NAU3736 CIR334
NAU6309 CGR6889
6.9 DPL0182
18.2 SWU11691
Chr15 XZV
0.0 NAU3736
14.8 SWU11691
Chr16 XZ
0.0 PGML1709**
22.5 SWU10627
26.2 PGML1309
26.4 Gh137
PGML4289 JESPR297
27.0 SWU13514 ICR00647
28.9 SWU20341
30.3 DPL0897
31.2 BNL1026 DPL0048
66.9 SWU10266
76.3 DC40065 C2_0011B
79.9 DC20124 SWU10214
85.0 Gh56
90.7 NAU5120
93.4 NAU862
97.4 CGR6802
106.4 HAU1129
111.8 NAU2984
123.8 SWU10062*
135.5 SWU10094
149.8 SWU10060
150.9 SWU10054
151.1 SWU10056
158.5 SWU10037
158.8 ICR00010
162.6 SWU10038
180.5 ICR00016##
Chr16 XZV
0.0 HAU3081
11.0 NAU747
39.1 HAU1129
59.5 C2_0011B
61.1 SWU10211
66.6 SWU10266
99.8 SWU18366
119.7 SWU18579 CGR5506
123.9 CGR5925
0.0 DPL0897
1.3 SWU20341 HAU1873
1.6 SWU13514
2.5 JESPR297
5.4 ICR00647

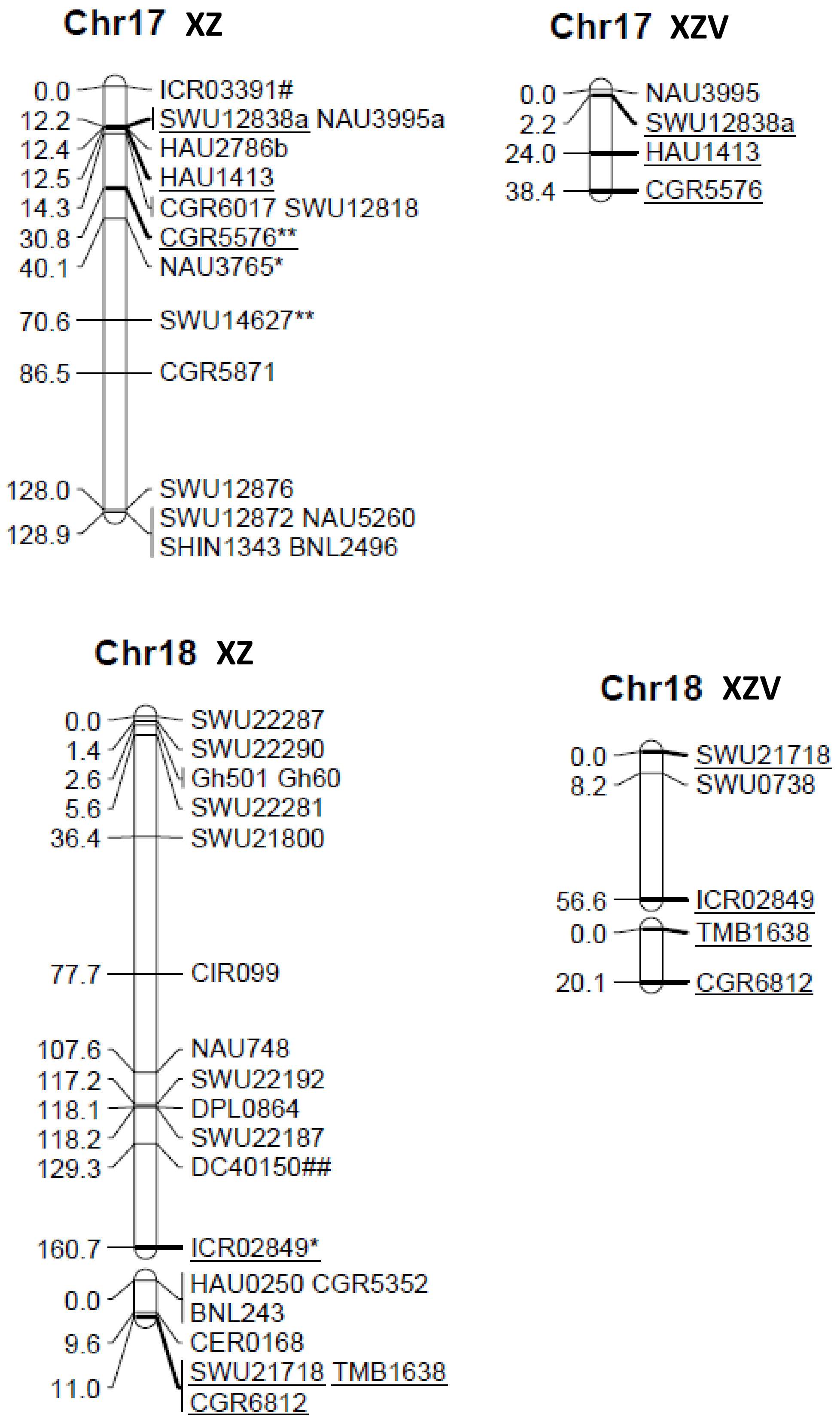
Chr17 XZ
0.0
12.2
12.4
12.5
14.3
30.8
40.1
70.6
86.5
128.0
128.9
ICR03391#
SWU12838a NAU3995a
HAU2786b
HAU1413
CGR6017 SWU12818
CGR5576**
NAU3765*
SWU14627**
CGR5871
SWU12876
SWU12872 NAU5260
SHIN1343 BNL2496
Chr17 XZV
0.0
2.2
24.0
38.4
NAU3995
SWU12838a
HAU1413
CGR5576
Chr18 XZ
0.0
1.4
2.6
5.6
36.4
77.7
107.6
117.2
118.1
118.2
129.3
160.7
SWU22287
SWU22290
Gh501 Gh60
SWU22281
SWU21800
CIR099
NAU748
SWU22192
DPL0864
SWU22187
DC40150##
ICR02849*
0.0
9.6
11.0
HAU0250 CGR5352
BNL243
CER0168
SWU21718 TMB1638
CGR6812
Chr18 XZV
0.0
8.2
56.6
SWU21718
SWU0738
ICR02849
0.0
20.1
TMB1638
CGR6812

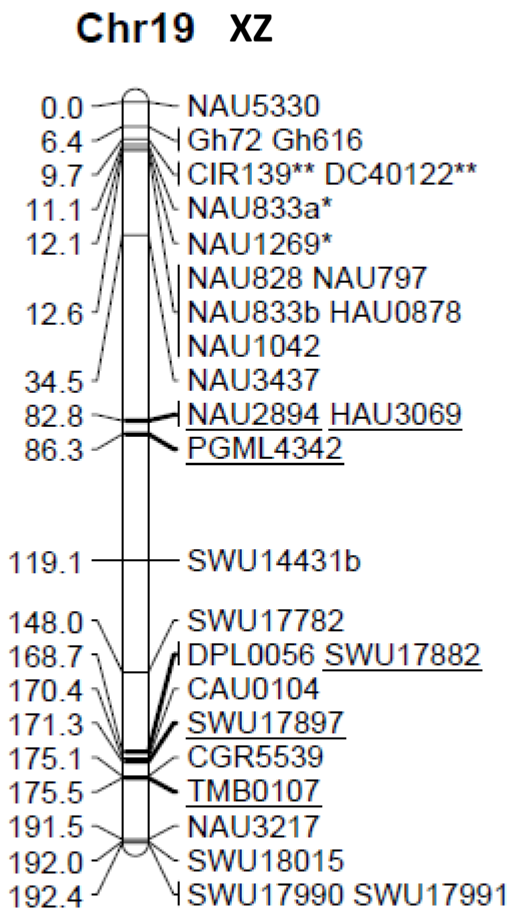
Chr19 XZ
0.0 NAU5330
6.4 Gh72 Gh616
9.7 CIR139** DC40122**
11.1 NAU833a*
12.1 NAU1269*
12.6 NAU828 NAU797
NAU833b HAU0878
NAU1042
34.5 NAU3437
82.8 NAU2894 HAU3069
86.3 PGML4342
119.1 SWU14431b
148.0 SWU17782
168.7 DPL0056 SWU17882
170.4 CAU0104
171.3 SWU17897
175.1 CGR5539
175.5 TMB0107
191.5 NAU3217
192.0 SWU18015
192.4 SWU17990 SWU17991

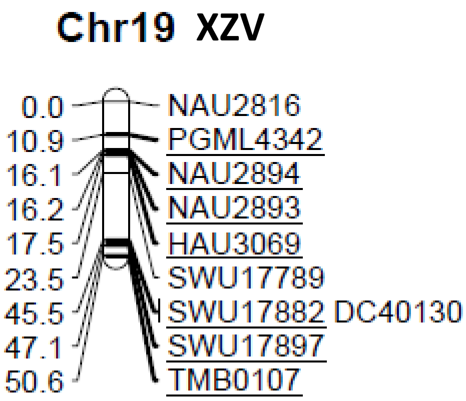
Chr19 XZV
0.0 NAU2816
10.9 PGML4342
16.1 NAU2894
16.2 NAU2893
17.5 HAU3069
23.5 SWU17789
45.5 SWU17882 DC40130
47.1 SWU17897
50.6 TMB0107

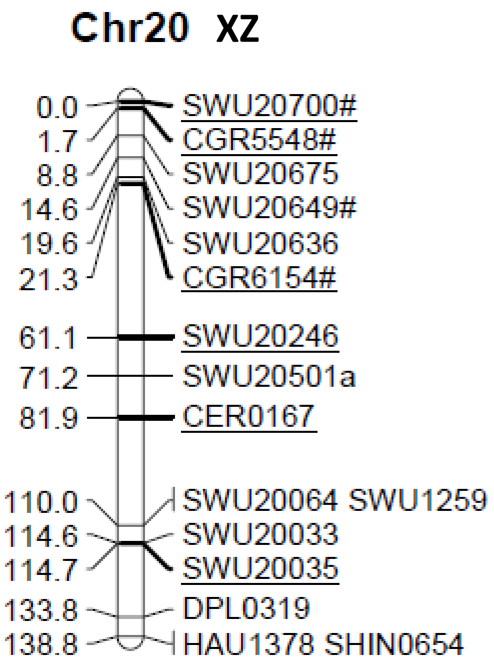
Chr20 XZ
0.0 SWU20700#
1.7 CGR5548#
8.8 SWU20675
14.6 SWU20649#
19.6 SWU20636
21.3 CGR6154#
61.1 SWU20246
71.2 SWU20501a
81.9 CER0167
110.0 SWU20064 SWU1259
114.6 SWU20033
114.7 SWU20035
133.8 DPL0319
138.8 HAU1378 SHIN0654

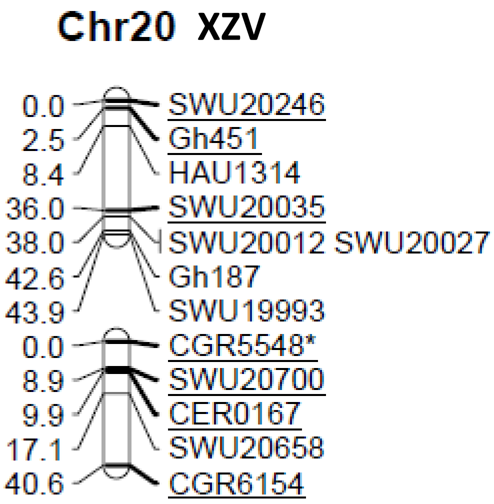
Chr20 XZV
0.0 SWU20246
2.5 Gh451
8.4 HAU1314
36.0 SWU20035
38.0 SWU20012 SWU20027
42.6 Gh187
43.9 SWU19993
0.0 CGR5548*
8.9 SWU20700
9.9 CER0167
17.1 SWU20658
40.6 CGR6154

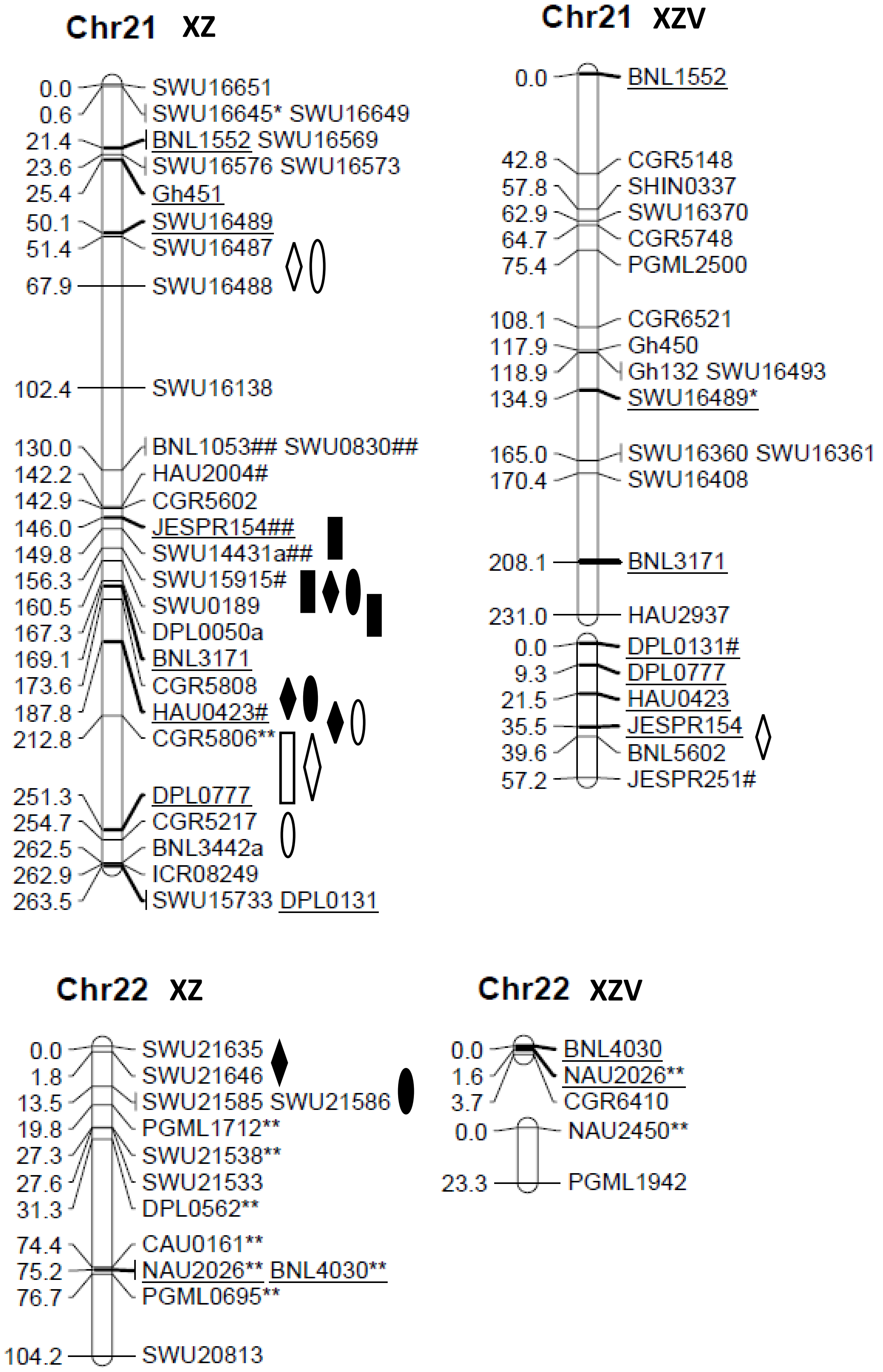

Chr21 XZ
0.0 SWU16651
0.6 SWU16645* SWU16649
21.4 BNL1552 SWU16569
23.6 SWU16576 SWU16573
25.4 Gh451
50.1 SWU16489
51.4 SWU16487
67.9 SWU16488
102.4 SWU16138
130.0 BNL1053## SWU0830##
142.2 HAU2004#
142.9 CGR5602
146.0 JESPR154##
149.8 SWU14431a##
156.3 SWU15915#
160.5 SWU0189
167.3 DPL0050a
169.1 BNL3171
173.6 CGR5808
187.8 HAU0423#
212.8 CGR5806**
251.3 DPL0777
254.7 CGR5217
262.5 BNL3442a
262.9 ICR08249
263.5 SWU15733 DPL0131
Chr21 XZV
0.0 BNL1552
42.8 CGR5148
57.8 SHIN0337
62.9 SWU16370
64.7 CGR5748
75.4 PGML2500
108.1 CGR6521
117.9 Gh450
118.9 Gh132 SWU16493
134.9 SWU16489*
165.0 SWU16360 SWU16361
170.4 SWU16408
208.1 BNL3171
231.0 HAU2937
0.0 DPL0131#
9.3 DPL0777
21.5 HAU0423
35.5 JESPR154
39.6 BNL5602
57.2 JESPR251#
Chr22 XZ
0.0 SWU21635
1.8 SWU21646
13.5 SWU21585 SWU21586
19.8 PGML1712**
27.3 SWU21538**
27.6 SWU21533
31.3 DPL0562**
74.4 CAU0161**
75.2 NAU2026** BNL4030**
76.7 PGML0695**
104.2 SWU20813
Chr22 XZV
0.0 BNL4030
1.6 NAU2026**
3.7 CGR6410
0.0 NAU2450**
23.3 PGML1942

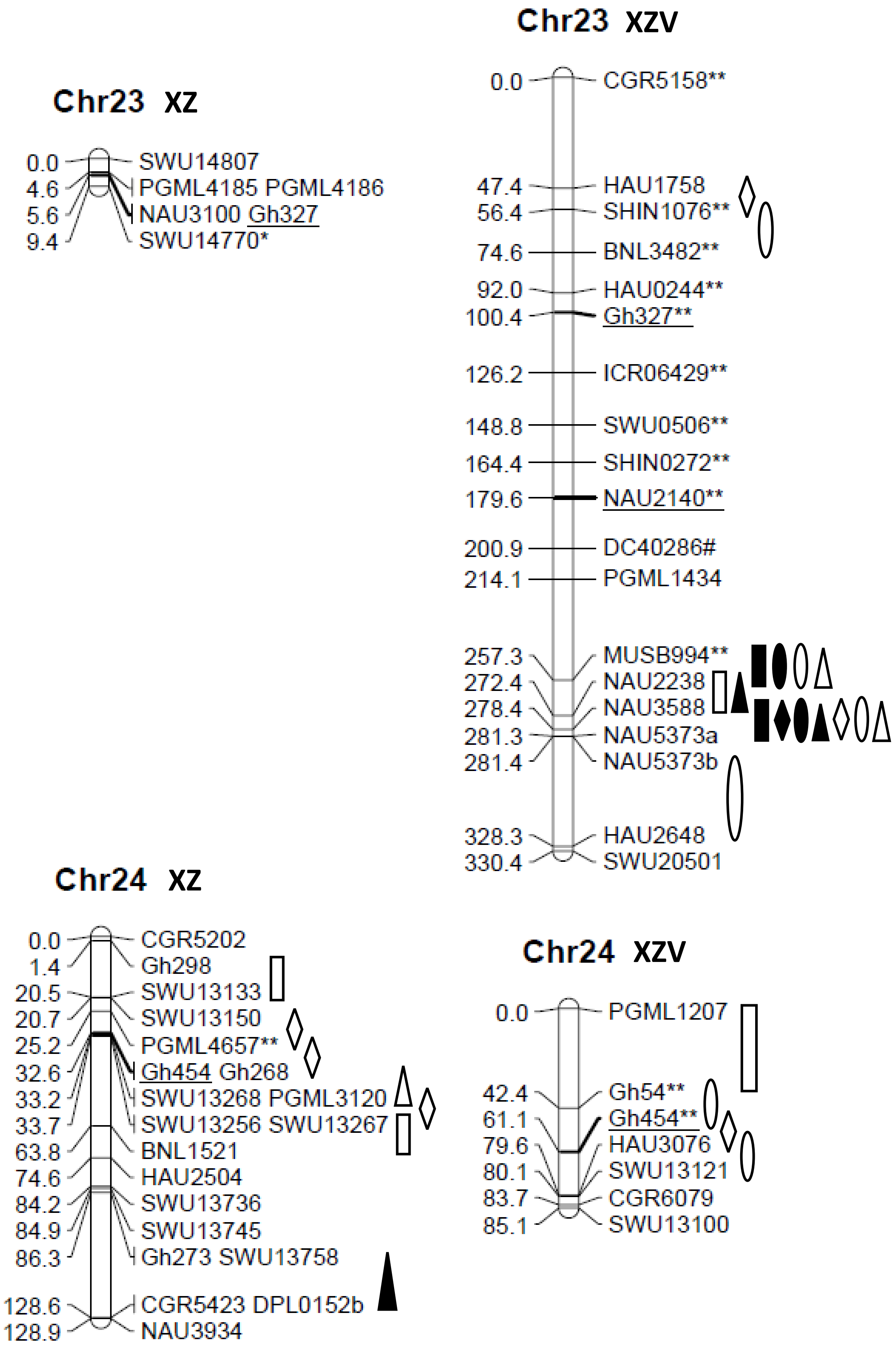

Chr23 XZ
0.0 SWU14807
4.6 PGML4185 PGML4186
5.6 NAU3100 Gh327
9.4 SWU14770*
Chr23 XZV
0.0 CGR5158**
47.4 HAU1758
56.4 SHIN1076**
74.6 BNL3482**
92.0 HAU0244**
100.4 Gh327**
126.2 ICR06429**
148.8 SWU0506**
164.4 SHIN0272**
179.6 NAU2140**
200.9 DC40286#
214.1 PGML1434
257.3 MUSB994**
272.4 NAU2238
278.4 NAU3588
281.3 NAU5373a
281.4 NAU5373b
328.3 HAU2648
330.4 SWU20501
Chr24 XZ
0.0 CGR5202
1.4 Gh298
20.5 SWU13133
20.7 SWU13150
25.2 PGML4657**
32.6 Gh454 Gh268
33.2 SWU13268 PGML3120
33.7 SWU13256 SWU13267
63.8 BNL1521
74.6 HAU2504
84.2 SWU13736
84.9 SWU13745
86.3 Gh273 SWU13758
128.6 CGR5423 DPL0152b
128.9 NAU3934
Chr24 XZV
0.0 PGML1207
42.4 Gh54**
61.1 Gh454**
79.6 HAU3076
80.1 SWU13121
83.7 CGR6079
85.1 SWU13100

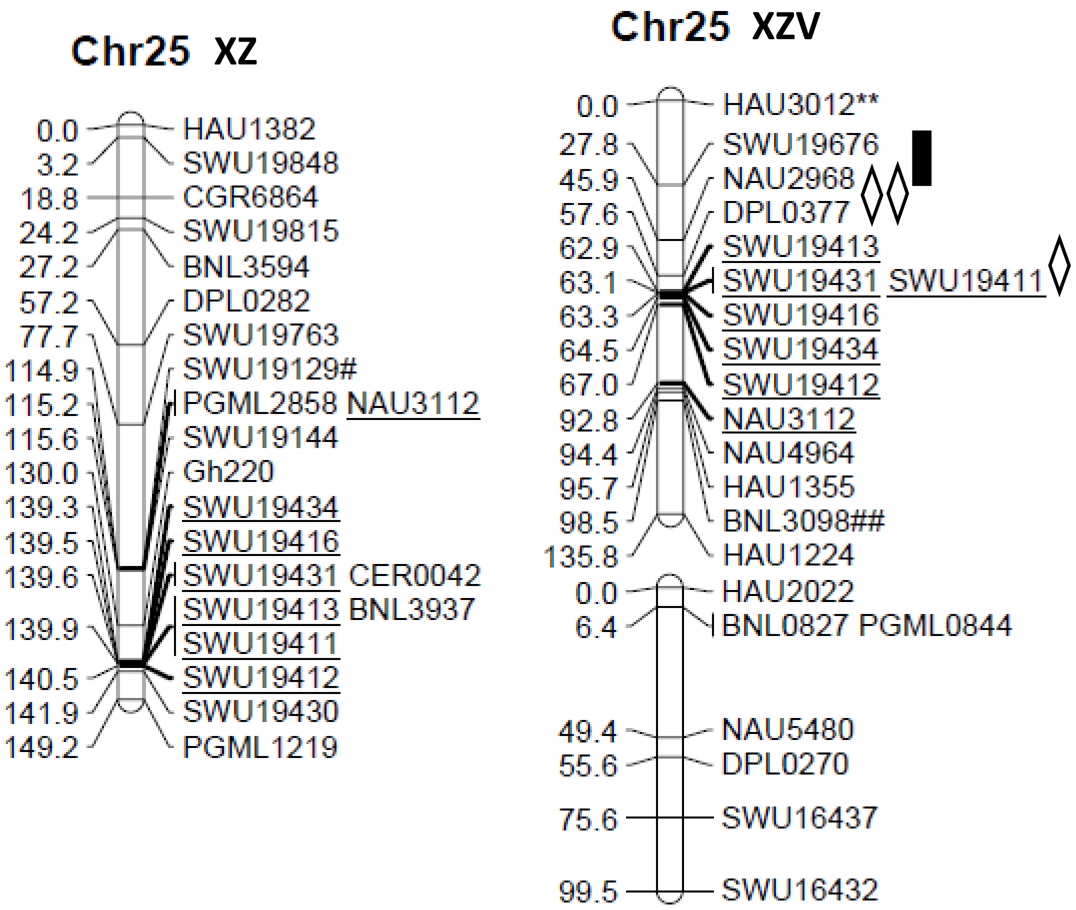

Chr25 XZ
0.0 HAU1382
3.2 SWU19848
18.8 CGR6864
24.2 SWU19815
27.2 BNL3594
57.2 DPL0282
77.7 SWU19763
114.9 SWU19129#
115.2 PGML2858 NAU3112
115.6 SWU19144
130.0 Gh220
139.3 SWU19434
139.5 SWU19416
139.6 SWU19431 CER0042
139.9 SWU19413 BNL3937
SWU19411
140.5 SWU19412
141.9 SWU19430
149.2 PGML1219
Chr25 XZV
0.0 HAU3012**
27.8 SWU19676
45.9 NAU2968
57.6 DPL0377
62.9 SWU19413
63.1 SWU19431 SWU19411
63.3 SWU19416
64.5 SWU19434
67.0 SWU19412
92.8 NAU3112
94.4 NAU4964
95.7 HAU1355
98.5 BNL3098##
135.8 HAU1224
0.0 HAU2022
6.4 BNL0827 PGML0844
49.4 NAU5480
55.6 DPL0270
75.6 SWU16437
99.5 SWU16432

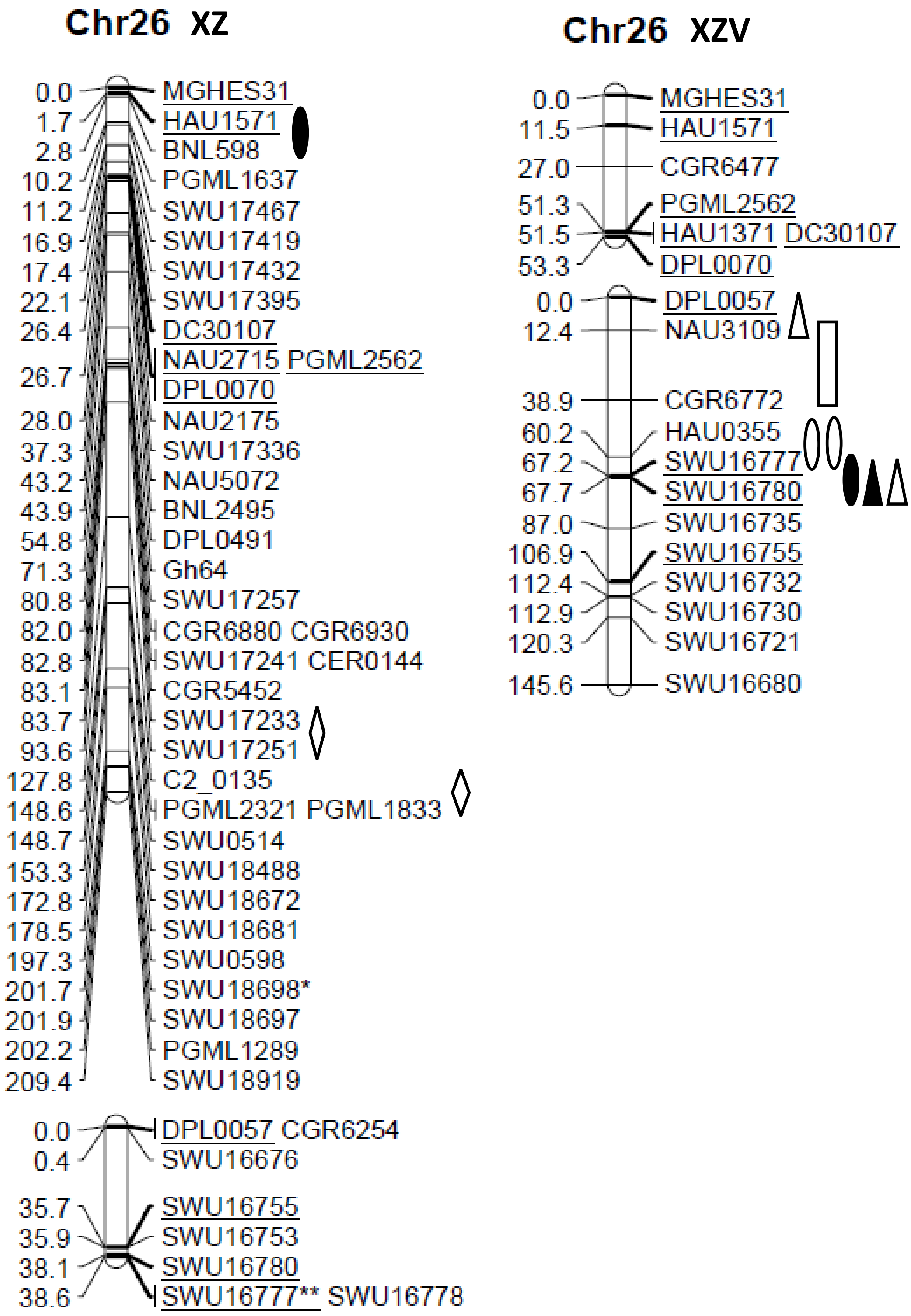
Chr26 XZ
Chr26 XZV
0.0 MGHES31
1.7 HAU1571
2.8 BNL598
10.2 PGML1637
11.2 SWU17467
16.9 SWU17419
17.4 SWU17432
22.1 SWU17395
26.4 DC30107
26.7 NAU2715 PGML2562
DPL0070
28.0 NAU2175
37.3 SWU17336
43.2 NAU5072
43.9 BNL2495
54.8 DPL0491
71.3 Gh64
80.8 SWU17257
82.0 CGR6880 CGR6930
82.8 SWU17241 CER0144
83.1 CGR5452
83.7 SWU17233
93.6 SWU17251
127.8 C2_0135
148.6 PGML2321 PGML1833
148.7 SWU0514
153.3 SWU18488
172.8 SWU18672
178.5 SWU18681
197.3 SWU0598
201.7 SWU18698*
201.9 SWU18697
202.2 PGML1289
209.4 SWU18919
0.0 DPL0057 CGR6254
0.4 SWU16676
35.7 SWU16755
35.9 SWU16753
38.1 SWU16780
38.6 SWU16777** SWU16778
0.0 MGHES31
11.5 HAU1571
27.0 CGR6477
51.3 PGML2562
51.5 HAU1371 DC30107
53.3 DPL0070
0.0 DPL0057
12.4 NAU3109
38.9 CGR6772
60.2 HAU0355
67.2 SWU16777
67.7 SWU16780
87.0 SWU16735
106.9 SWU16755
112.4 SWU16732
112.9 SWU16730
120.3 SWU16721
145.6 SWU16680

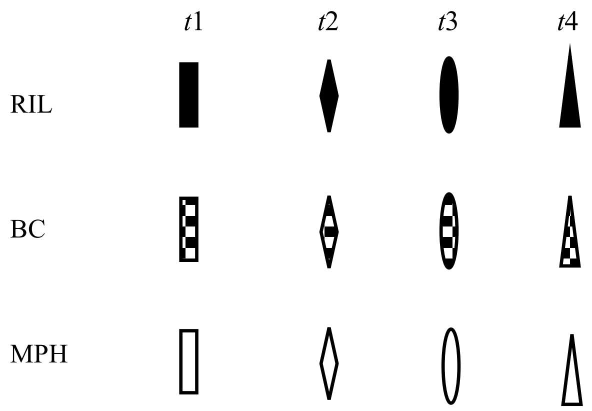

Figure S1 Locations of QTLs controlling boll numbers per plant identified in two hybrids

* and **, segregation distortion significant at P = 0.05 and 0.01 levels, respectively.
